# Cycloadditions as a Sweet Route to ‘Double *C*-Glycosylation’

**DOI:** 10.3390/biom15060905

**Published:** 2025-06-19

**Authors:** Kevin P. P. Mahoney, Rosemary Lynch, Rhea T. Bown, Sunil V. Sharma, Piyasiri Chueakwon, G. Richard Stephenson, David B. Cordes, Alexandra M. Z. Slawin, Rebecca J. M. Goss

**Affiliations:** 1 School of Chemistry, Integrated Institute of Engineering, and BSRC, University of St. Andrews, St. Andrews KY16 9ST, UK; rl96@st-andrews.ac.uk (R.L.); svs4@st-andrews.ac.uk (S.V.S.); pc228@st-andrews.ac.uk (P.C.); 2 TopChem Pharmaceuticals, Ballymote Business Park, Carrownanty, F56 RX08 Ballymote, Ireland; 3 School of Chemistry, Pharmacy and Pharmacology, University of East Anglia, Norwich NR4 7TJ, UK; g.r.stephenson@uea.ac.uk; 4 School of Chemistry, University of St. Andrews, St. Andrews KY16 9ST, UK; dbc21@st-andrews.ac.uk (D.B.C.); amzs@st-andrews.ac.uk (A.M.Z.S.)

**Keywords:** cycloaddition, biomimetic, granaticin, natural product analogue

## Abstract

Pharmaceuticals, such as the antibiotic erythromycin, and sodium-dependent glucose transporter (SGLT1 & SGTL2) inhibitors such as Bexagliflozin (diabetes) and Sotagliflozin (heart disease), are often sugar-decorated (glycosylated). Glycosylation is a key component of the binding motif in SGLT inhibitors and, in natural products, glycosylation often confers improved bioactivity and bioavailability. Whilst a single *C*-glycoside link between a sugar moiety and its aglycone core is a common feature in natural products isolated to date, only a small number, including the antibiotics granaticin and sarubicin, are covalently bonded twice to a single sugar moiety. The way in which this “double *C*-glycosylation” is naturally mediated is not yet known, yet has been speculated on. Here, we report the exploration and development of a potentially biomimetic procedure that utilises intermolecular cycloaddition chemistry to access new “double *C*-glycosylated” products and enables the creation of bridged polycyclic ethers from a common maltol-derived oxidopyrylium salt precursor.

## 1. Introduction

**Figure 1:**
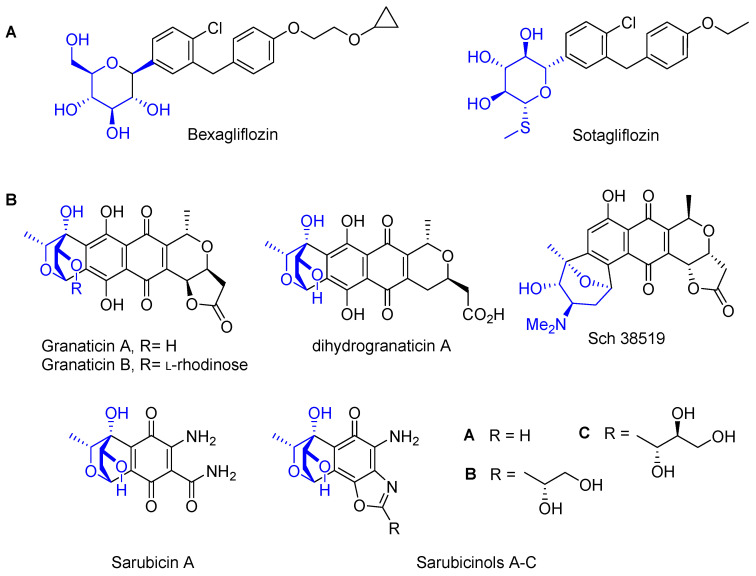
(**A**) Two examples of synthetic *C*-glycoside pharmaceuticals. (**B**) Structures of natural products; granaticin, dihydrogranaticin A, Sch 38519, sarubicin A and sarubicinols: *C*-glycoside and “double *C*-glycoside” groups are shown in blue.

Glycosides are well known for their potent biological activities (e.g., antibiotic natural product erythromycin). Synthetic *C*-glycosides with potent enzyme inhibitory properties are clinically used as antidiabetics and to treat cardiovascular disease (e.g., Bexagliflozin and Sotagliflozin (Figure 1)). *C*-Glycosides are important in medicine, providing improved stability against enzymatic hydrolysis and better bioavailability in comparison to standard *O*-glycosides.

Whilst glycosides and *C*-glycosides are common in nature, the doubly bonded motif “double *C*-glycoside” is rarer. Granaticin, Sch 38519, sarubicin and sarubicinols (Figure 1) are the only known examples of natural product “double *C*-glycosides” that have been isolated to date. They exhibit a rare structural motif of a carbohydrate moiety attached to an aglycone via two covalent C-C linkages (see Figure 1). Sarubicin A, isolated in 1980 from *Streptomyces* strain JA2861, is active against both Gram-positive and Gram-negative bacteria [1,2]. The more recently identified sarubicinols A–C were isolated from *Streptomyces* sp. Hu186 and showed moderate toxicity against four cancer cell lines (A549, HCT-116, HepG2, and 4T1) with IC_50_ values of 0.7−14.7 μM [3].

Granaticin was first isolated from *Streptomyces olivaceus* in 1957 [4], and has since been isolated from several other strains, including *S. vietnamensis* [5] and *S. violaceoruber* [6]. It is highly active against Gram-positive bacteria and protozoa [4]. It also exhibits activity against P-388 lymphocytic leukaemia in mice (T/C 166% at 1.5 mg/kg) and cytotoxicity against HeLa-KB (subline of Henrietta Lacks cell line) cells (ED_50_ 1.6 μg/mL) [6,7]. Sch 38519, isolated from a *Thermomonospora* species, is also reported to have activity against both Gram-positive and Gram-negative bacteria [8].

The structures of both granaticin and sarubicin A include the unusual motif in which a 2,6-dideoxyhexose sugar is attached to the aglycone via two covalent bonds, forming a structurally complex and intriguing 2-oxabicyclo[2.2.2]oct-5-ene moiety (a chiral bridged polycyclic ether). Sch 38519 contains an equally fascinating 8-oxabicyclo[3.2.1]oct-5-ene substructure in which an aminosugar is attached by two carbon–carbon linkages to the benzoisochromane quinone (BIQ) aglycone. The biosynthesis of the granaticin BIQ aglycone has been established as being via a type II polyketide synthase (PKS) involving an acetate and seven malonate units in an iterative chain extension process [6]. The PK chain is elaborated by a number of post-PKS enzymes that mediate the cyclisation and aromatisation to furnish the BIQ core. Attachment of the carbohydrate moiety, however, remains unclear. Only a single glycosyltransferase homologue has been identified within the biosynthetic gene cluster. As the gene cluster has been heterologously expressed, it is expected that all of the required genes for granaticin biosynthesis are present within the cluster [9].

**Scheme 1:**
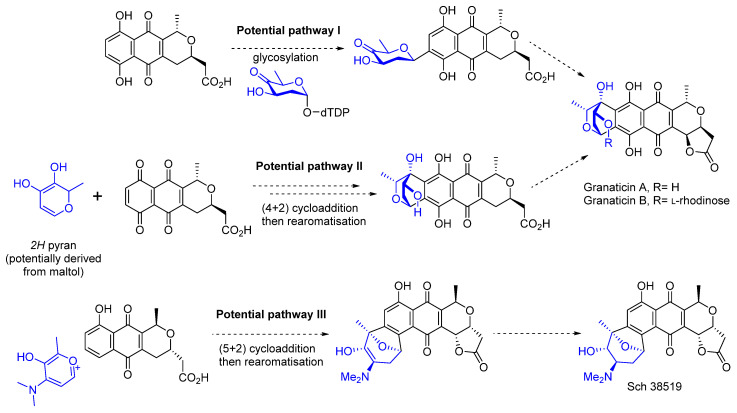
Potential routes to the “double *C*-glycosylation”: Pathway I glycosylation and aldol, Pathway II cycloaddition via Diels–Alder followed by re-aromatisation and reduction. Possible route to Sch 38519 via (5 + 2) cycloaddition.

There are two possibilities for the mechanism of attachment of the carbohydrate. The first involves glycosylation *ortho* to the post PKS C8 hydroxyl of the 8-hydroxy BIQ aglycone. This would then be followed by a *6-exo-trig* cyclisation (Scheme 1, Pathway I). In this first route, a single glycosyltransferase would need to be responsible for mediating the double *C*-linkage to the 2,6-dideoxyhexose sugar and then subsequent attachment of the L-rhodinose in granaticin B. Whilst glycosyltransferases are known to be fairly substrate flexible, there is currently no precedent in nature for a single glycosyltransferase mediating such a complex series of reactions. The alternative to these mechanisms in the generation of granaticin could be a (4 + 2) cycloaddition reaction (Diels–Alder), followed by re-aromatisation, between the BIQ aglycone and the carbohydrate moiety, forming both *C*-glycosidic bonds (Scheme 1, Pathway II). Another route is appealing as a (5 + 2) cycloaddition can potentially be invoked to rationalise the generation of Sch 38519 (Scheme 1, Pathway III).

**Scheme 2:**
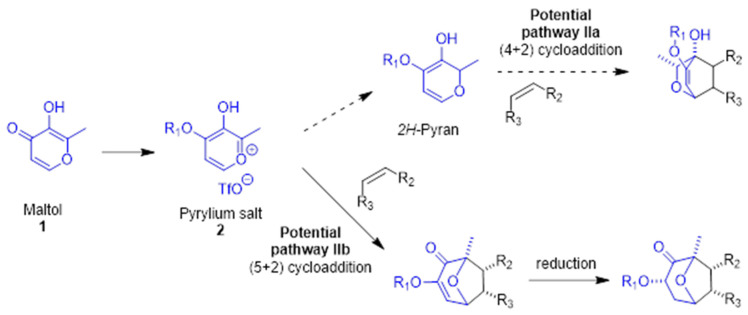
Exploration of the maltol-derived pyrylium salt (**2**) in cycloaddition reactions.

In this study, we explore the chemical feasibility, and potential utility, of such an alternative pathway that involves the spontaneous cycloaddition of a maltol-derived species with an aglycone core. We investigate the cycloaddition with series of readily available alkenes seeing whether such a process could be harnessed to afford one step access to series of new to nature “double *C*-glycosylated” products (Scheme 2). Maltol is known to be an elimination product of dTDP-4-keto-2,6-dideoxy-α-D-glucose [10], the putative precursor for the glycosyl transferase route (Scheme 1, Pathway I), and the potential precursor of the granaticin aglycone’s cycloaddition partner.

We determined that the pyrylium salt (**2**) itself might be utilised in accessing alternative “double *C*-glycosylation” patterns via a (5 + 2) cycloaddition (Scheme 1, Potential pathway III). To this end, we explored the chemical feasibility of access to double *C*-glycosides via a (5 + 2) cycloaddition from a maltol (**1**)-derived pyrylium salt (**2**). We demonstrate that not only is this transformation chemically feasible, but that it provides a useful approach to generating analogues in which a carbohydrate moiety is attached via two *C*-glycosidic bonds.

Maltol (**1**) is activated using methyl triflate, forming an oxidopyrylium salt (**2**). The (5 + 2) cycloaddition then takes place between this species and a chosen alkene (Scheme 2, Potential pathway IIb). A rhodium catalyst on alumina may be used to saturate the alkene of the carbohydrate, giving the “double *C*-glycoside” [11].

## 2. Materials and Methods

*General Experimental*

All solvents were purchased from Merck (Darmstadt, Germany) or Fisher Scientific (Loughborough, UK). All reagents and equipment were purchased from the following companies as indicated in the text: Merck (Darmstadt, Germany), Fisher Scientific (Loughborough, UK), VWR (part of Avantor Inc, Radnor, PA 19087), Fluorochem (Hadfield, UK), BOC (Woking, UK), Buchi (Newmarket, UK), Biotage (751 03 Uppsala, Sweden), Rigaku Corporation (Tokyo, Japan).

Characterisation of products, including NMR spectra are available in the Supplementary Materials.

### 2.1. Preparation of Pyrylium Salt (***2***)

To a solution of maltol (Merck) (5 g, 39.6 mmol, 1.0 equiv.) in dichloromethane (VWR) (10 mL) was added methyl trifluoromethanesulfonate (Fluorochem) (6.7 mL, 59.4 mmol, 1.5 equiv.). The reaction was stirred at reflux for 4 h, cooled to room temperature and then evaporated under reduced pressure to afford a white solid, which was recrystallised from ethyl acetate, 7.7 g, 64% yield.

### 2.2. General Procedure or Preparation of Cycloadducts

In an oven dried flask (Fisher Scientific), to a solution of the pyrylium salt (0.1 g, 0.34 mmol, 1 equiv.) in dry THF (Merck) (10 mL) under an atmosphere of argon (BOC), an alkene or alkyne (1.7 mmol, 5 equiv.) was added. DIPEA (Fisher Scientific) (0.09 mL, 0.51 mmol, 1.5 equiv.) was then added dropwise before heating to reflux. The reaction mixture was stirred under reflux for 16 h, before cooling to room temperature. The solvent was removed by rotary evaporation (Buchi) under reduced pressure. The crude mixture was then loaded onto a silica column (Biotage) and first washed with 20% diethyl ether (Merck) in hexane (Merck), before being eluted with 100% diethyl ether. The product was then purified using automated column chromatography (Biotage Isolera 4) utilising a gradient from 20% diethyl ether in hexane to 100% diethyl ether.

### 2.3. Single Crystal X-Ray Diffraction

X-ray diffraction data for compounds **6** and **9b** were collected at 173 K using a Rigaku MM-007HF High Brilliance RA generator/confocal optics [Cu Kα radiation (λ = 1.54187 Å)] with a XtaLAB P100 diffractometer (Rigaku Corporation, Tokyo, Japan). Intensity data were collected using both ω and φ steps accumulating area detector images spanning at least a hemisphere of reciprocal space. Data for both compounds analysed were collected using CrystalClear (v2.1)(Rigaku) [12] and processed (including correction for Lorentz, polarization and absorption) using either CrystalClear or CrysAlisPro (v1.171.42.83a)(Rigaku) [13]. Structures were solved by direct methods (SIR2011) [14] and refined by full-matrix least-squares against F^2^ (SHELXL-2019/3) [15]. Non-hydrogen atoms were refined anisotropically, and hydrogen atoms were refined using a riding model. All calculations were performed using the Olex2 (v1.5)(University of Durham, Durham, UK) [16] interface. Selected crystallographic data are presented in Table 1. CCDC 2447755-2447756 contains the supplementary crystallographic data for this paper. These data can be obtained free of charge from the Cambridge Crystallographic Data Centre via www.ccdc.cam.ac.uk/structures.

## 3. Results and Discussion

### 3.1. Initial Exploration of the Cycloaddition Reaction

A variety of oxidopyrylium ylides have previously been reported to undergo (5 + 2) cycloadditions with an appropriate alkene or alkyne [17,18,19,20]. Maltol (**1**), a cheap and readily available 4-pyrone, represents an attractive candidate for the exploration of cycloaddition chemistry as a means of accessing series of new to nature glycosylated natural products. Maltol (**1**) was converted into its corresponding pyrylium salt (**2**) by heating to 70 °C with methyl triflate for 4 h; in this reaction, the hydroxyl group at the 4 position is alkylated to give the positively charged oxocarbenium containing pyrylium salt (**2**). A similar approach to the formation of a pyrylium salt has been demonstrated by Wender and Murelli [21,22]. Whilst we found that this salt could be generated in situ and subsequently utilised directly in the cycloaddition reaction, thereby reducing the number of discrete steps, such an approach makes product purification more problematic, due to the presence of unreacted maltol (**1**) and methyl triflate. Instead, we found it preferable to first generate the pyrylium salt (**2**), purify it by recrystallisation, then utilise the purified material in the cycloaddition step.

### 3.2. Solvent Screening

Using dimethyl acetylenedicarboxylate (DMAD), initial explorations of the cycloaddition promisingly revealed that in the presence of a non-nucleophilic base, such as dimethyl aniline (DMA), the dipolar oxidopyrylium species could be generated and the reaction could proceed, albeit in a low yield (9%). Products could also be synthesised from diethyl maleate (Product **3a**, 9% yield), and cinnamyl acetate (Product **5**, 8% yield). Encouraged by the demonstrated feasibility of the reaction with the maltol derived pyrylium salt (**2**), in generating “double *C*-glycosylated” products, we sought to determine improved conditions for the reaction. To this end, we systematically screened a series of solvents prior to exploring which alkenes might be elaborated. Diethyl maleate was selected as an electron-poor alkene, and thus more challenging test substrate, for the screening process in order to maximise the optimisation. The solvent, under investigation, and diethyl maleate were added to the purified pyrylium salt (**2**). The reactions were refluxed for 16 h in the presence of 1.5 equivalents of DMA and purified by column chromatography. Isolated yields for the major stereoisomers were obtained for each reaction and are given in Table 2.

The oxyether containing solvents tetrahydrofuran (THF) and diethyl ether resulted in the highest yields, though the reaction yield in dioxane remained very low. It was decided that going forward THF would be used as the solvent of choice to enable the reaction to be refluxed at a higher temperature.

### 3.3. Base Screening

Once we had selected THF as the solvent, in efforts to try to elevate the yield, we explored the impact of the base on the reaction (1.5 and 3 equivalents in each case). The use of sterically hindered bases in promoting cycloaddition reactions of oxidopyrylium salts derived from kojic acid was reported to be beneficial [21,22]; hence, six sterically hindered bases were selected for screening. The p*K*a and aromaticity of bases were also taken into account. Upon initial analysis by thin layer chromatography (TLC), five of the reactions were discarded, and an isolated yield for a single stereoisomer was obtained for the remaining reactions. These results are given in Table 3.

**Scheme 3:**
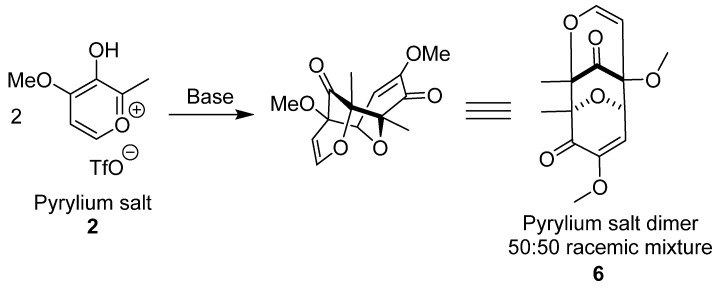
Formation of the pyrylium salt dimeric product (**6**) generated during microwave heating.

**Figure 2:**
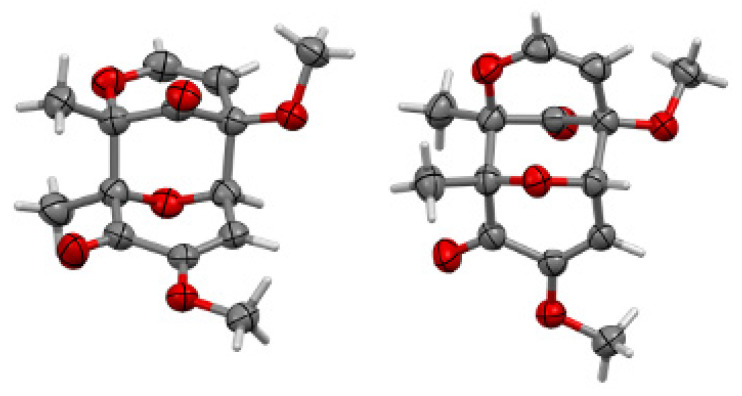
Thermal ellipsoid plot (50% probability ellipsoids) of the crystal structure of the two enantiomers in the structure of the racemic pyrylium salt dimer (**6**); grey = carbon; red = oxygen; white = hydrogen.

The use of 4-(dimethylamino)pyridine (DMAP) was shown to elevate the yields, though its employment also promoted ester hydrolysis. This was undesirable as many of the chosen alkenes were esters; for consistency, *N*,*N*-diisopropylethylamine (DIPEA) was chosen as the most suitable base going forward, using 1.5 equivalents. A single diastereoisomer could be isolated in a 25% yield from this reaction, a modest increase in yield of 17% over the first two steps compared to the initial experiments conducted. The use of microwave heating, in order to reduce reaction times, was also explored. This resulted in increased dimerization of the pyrylium salt (**2**) and poorer yields. Potentially, the dimerization derives from attack of the enolate of one pyrylium salt molecule, onto the oxocarbenium carbon of a second molecule [23] (see Scheme 3). The structure of the pyrylium salt dimer (**6**) was confirmed by single crystal X-ray diffraction (Figure 2).

Pyrylium dimers are well known and can themselves be used to form (5 + 2) cycloaddition products [23,24,25]. Bejcek noted that the pyrylium dimer of maltol requires higher energy and is formed in a concerted process compared to the dimer of allomaltol which forms more easily in a stepwise process [23]. Schiavone showed that pyrylium dimers react well with alkynes under microwave conditions to form (5 + 2) cycloaddition products [24]. In our work, we observed the pyrylium dimer as a byproduct when we used microwave conditions; this is likely due to the lower reactivity of alkenes compared to alkynes for cycloaddition reactions.

### 3.4. Exploring Substrate Scope

**Figure 3:**
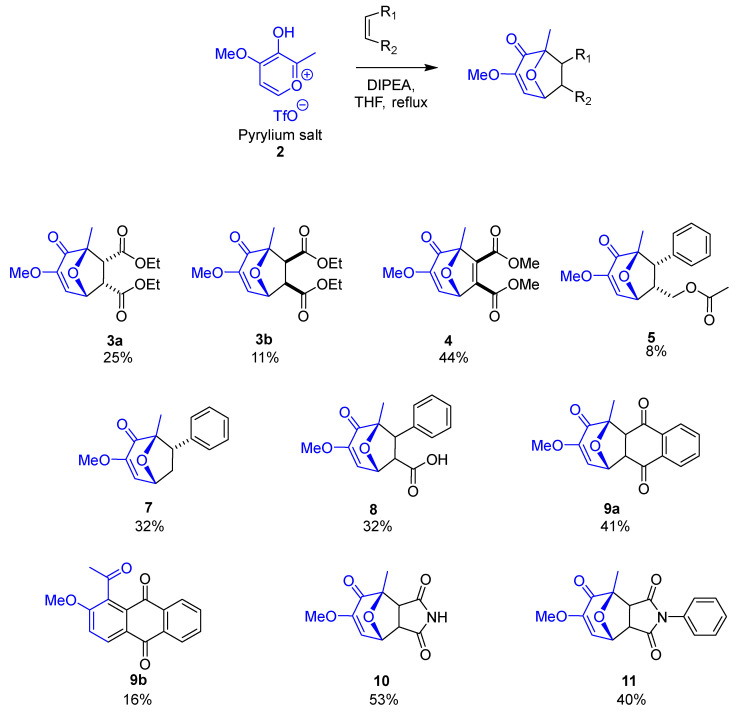
Isolated yields for a series of analogues accessed through applying the cycloaddition of the maltol-derived pyrylium salt (**2**). Reaction with 1,4-naphthoquinone yields both the cycloaddition product **9a** and the rearranged and aromatised anthraquinone **9b**.

With a series of conditions established that could potentially be applied to a range of substrates, without risk of hydrolysis, a series of alkenes and DMAD were selected to explore the scope of this reaction. All reactions were refluxed with the pre-prepared and purified pyrylium salt (**2**), in the presence of 1.5 equivalents of DIPEA, in THF, for 16 h. Each cycloadduct analogue was purified by column chromatography to obtain an isolated yield. These results are shown in Figure 3. The pyrylium salt dimer (**6**) was also isolated from a number of these reactions. The cycloaddition with diethyl maleate proceeded to give cycloadduct **3** as two diastereoisomers in an overall 36% yield. More electron-rich dimethyl acetylenedicarboxylate resulted in a higher yielding cycloaddition to afford **4** in a 44% overall yield. Though reaction with styrene proceeded to afford **7** in a 32% yield, cycloaddition reaction with the more sterically demanding cinnamyl acetate to afford **5** was poorly yielding at 8%, and reactions with both cinnamaldehyde and cinnamyl alcohol proved unsuccessful. Methyl *trans*-cinnamate underwent a reaction but suffered hydrolysis to give the carboxylic acid product **8** in a 32% isolated yield.

The cycloaddition of the pyridinium salt (**2**) with the structurally constrained maleimide, and *N*-phenylmaleimide both proceed with a reasonable yield to form **10** (53%) and **11** (40%), respectively. Whilst maltol-derived ylides have been shown to be more reactive than their kojic acid-derived allomaltol counterparts [23], their reaction paths are finely balanced with changes in conditions resulting in variable yields of desired products and by-products such as the pyrylium dimers. Comparing our work with that of Wender [22], we see that whilst we isolated only one diastereoisomer from the reaction with *N*-phenylmaleimide (**11**), Wender reported isolating a mixture of both the *exo* and *endo* diastereoisomers in a ratio of 1.8:1. By using dimethylaniline as the base and extending reaction times over 10 h, Wender achieved a combined yield of 98% for the two diastereoisomers. These results may be attributed to differences in the chemical reactivity of maltol- vs. kojic acid-derived pyrylium salts.

**Figure 4:**
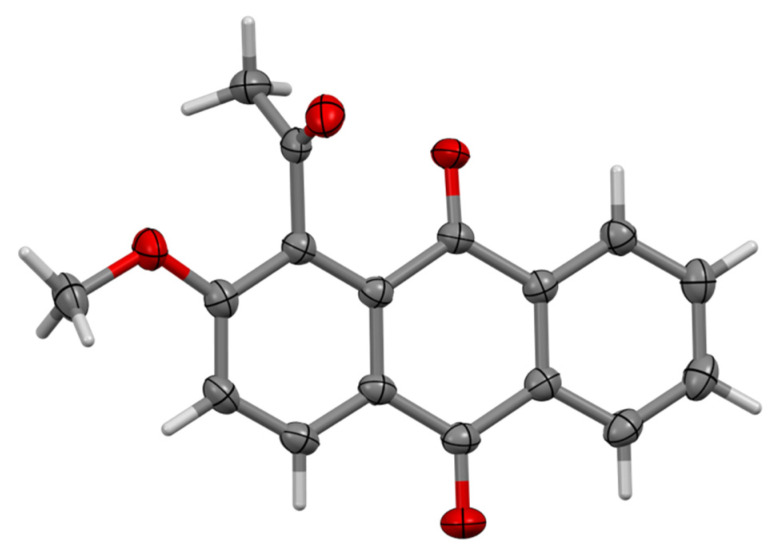
Thermal ellipsoid plot (50% probability ellipsoids) of the crystal structure of the elimination product (**9b**).

**Figure 5:**
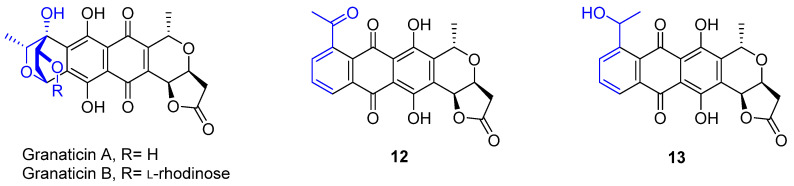
Two anthraquinones (**12**, **13**) are reported to occur as co-metabolites with granaticin [6,27].

Intriguingly, the cycloaddition with 1,4-naphthoquinone resulted not only in the expected product **9a** but also its elimination product, 1-acetyl-2-methoxyanthracene-9,10-dione **9b**, previously reported by Shah and Sethna [26] (characterized spectroscopically and by single crystal X-ray diffraction (Figure 4)). It is noteworthy that the analogous anthraquinones (**12**, **13**) have been isolated as co-metabolites of granaticin, showing that this rearrangement also occurs in natural fermentations (Figure 5) [6,27].

**Scheme 4:**
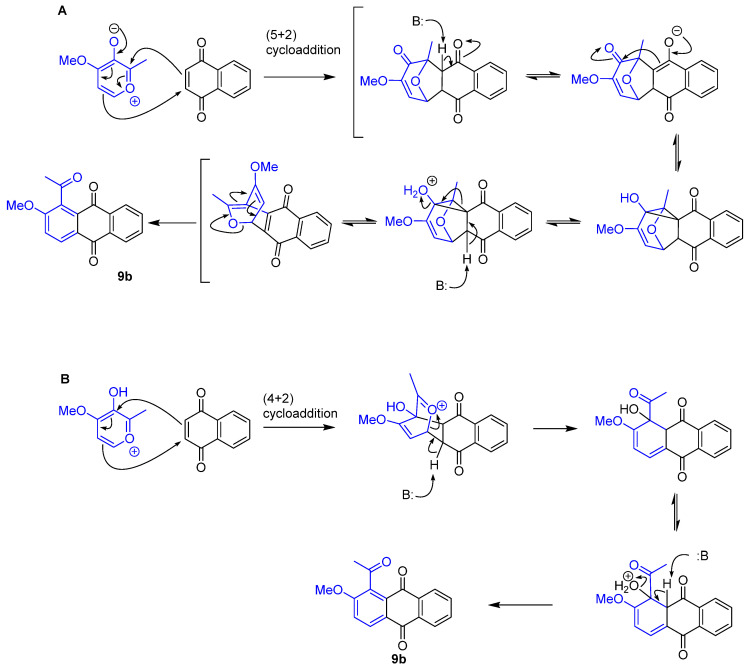
Potential routes for the formation of 1-acetyl-2-methoxyanthracene (**9b**) via (**A**), a (5 + 2) cycloaddition or via (**B**), a (4 + 2) cycloaddition.

We suggest two potential pathways via which **9b** could be formed. The first (Scheme 4A) involves the transient formation of the (5 + 2) cycloadduct. Under basic conditions, the enolization of a quinone carbonyl would occur. This, followed by attack of the carbohydrate carbonyl by the enolate, forms a spirocyclopropyl intermediate. Base-catalysed ring-opening of this group results in the reformation of the quinone, followed by elimination of water to give a 2H-pyran. An electrocyclic rearrangement then results in formation of the product [28]. The second pathway via which formation of this product could occur, involves attachment of the pyrylium salt to the naphthoquinone by a (4 + 2) cycloaddition (Scheme 4B). Base-catalysed ring-opening followed by protonation of the hydroxyl and loss of water, could potentially lead to formation of the product.

Interestingly, diethyl maleate was the only substrate for which we isolated two diastereoisomers. In some cases, such as styrene and cinnamyl acetate, sterics may disfavour one isomer; in other cases, it may be that yields of the second isomer were too low for it to be isolated during purification. A complication of these reactions is the poor stability of some of the products, which makes identification and isolation difficult. Bejcek showed that maltol-derived oxidopyrylium cycloadducts can undergo ring contraction under acidic conditions [29], indicating the reactive nature of these species. The diastereomers of diethyl maleate we isolated may be identified as *syn* configurations by consideration of the NOEs (see SI). This, in conjunction with the coupling constants and comparison with literature precedents [28], can be used to conclude that **3a** is the *endo* form and **3b** the *exo* form.

### 3.5. Assessment of Antibiotic Activity

Having explored a potential biogenesis via the cycloaddition of a maltol-derived dipolarophile, we set out to establish whether such a motif, and its derivatives, might confer antibiotic activity. Sch 38519 is an active antibiotic against Gram-positive and Gram-negative strains [8]. Many of the compounds generated contain a motif analogous to the motif resulting from “double *C*-glycosylation” in Sch 38519. To investigate whether this motif could potentially be an interesting pharmacophore, preliminary investigations into the bioactivity of these compounds were carried out. Disc diffusion assays were initially used to obtain a first assessment as to whether each analogue exhibited any activity against two important pathogenic bacteria *Staphylococcus aureus* and *Pseudomonas aeruginosa*. Compounds **3a**, **4**, **7**, **8**, **9b** and **11** showed no activity. Compound **10**, derived from maleimide, showed a low level of activity against both *S. aureus* and *P. aeruginosa*.

## 4. Conclusions

“Double *C*-glycosylation” is a rare biosynthetic event and very few natural products exhibiting this motif have been isolated to date; all of these, however, have interesting biological activity, and the curious biogenesis of this motif remains unknown. Aiming to expand the “Double *C*-glycoside” portfolio, enabling investigation of this motif as an interesting pharmacophore, we employed the cycloaddition of a maltol derivative to generate relevant cycloaddition products. We demonstrate a simple synthetic approach enabling easy access to a number of natural product-like analogues.

The cycloaddition is shown to be an effective approach to accessing such compounds; whether this is indeed the route utilised biosynthetically, remains to be determined, and is the focus of further study in our lab. A number of “double *C*-glycoside” analogues and their derivatives have been synthesised with one of these compounds showing very low-level antibacterial activity against Gram-negative species *P. aeruginosa*, providing a potentially interesting starting point for the design of novel antibiotics.

## Figures and Tables

**Table 1:** Selected crystallographic data.

	6	9b
formula	C_14_H_16_O_6_	C_17_H_12_O_4_
fw	280.27	280.27
crystal description	Colourless plate	Yellow prism
crystal size [mm^3^]	0.24 × 0.18 × 0.02	0.12 × 0.09 × 0.05
space group	*P* $\bar{1}$	*P* $\bar{1}$
*a* [Å]	9.2341 (3)	7.884 (3)
*b* [Å]	10.6447 (5)	8.055 (3)
*c* [Å]	14.4081 (6)	10.842 (3)
*α* [°]	77.390 (4)	92.509 (17)
*β* [°]	74.942 (4)	108.668 (19)
*γ* [°]	89.491 (3)	95.313 (13)
vol [Å]^3^	1332.89 (10)	647.5 (4)
*Z*	4	2
*ρ* (calc) [g/cm^3^]	1.397	1.437
*μ* [mm^−1^]	0.928	0.850
F(000)	592	292
reflections collected	12704	6785
independent reflections (*R*_int_)	4770 (0.0308)	2308 (0.0385)
parameters, restraints	369, 0	193, 0
GoF on *F*^2^	1.072	1.129
*R*_1_ [*I* > 2*σ*(*I*)]	0.0428	0.0644
*wR*_2_ (all data)	0.1270	0.1358
largest diff. peak/hole [e/Å^3^]	0.220, −0.241	0.512, −0.6620

**Table 2:** Isolated yields of diastereoisomers of diethyl maleate cycloadduct from solvent screen reactions.

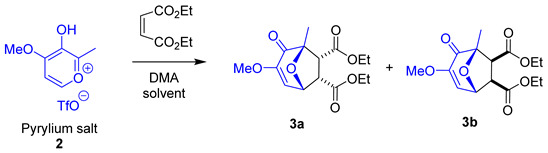			
Entry	Solvent	Isolated Yield of Diastereoisomer 3a (%)	Isolated Yield of Diastereoisomer 3b (%)
1	Neat	7	-
2	Dichloromethane	10	-
3	Toluene	6	-
4	Chloroform	14	-
5	1,4-Dioxane	7	-
6	Methanol	5	-
7	Acetonitrile	12	-
8	Tetrahydrofuran	17	7
9	Ethyl Acetate	9	-
10	Hexane	9	-
11	Diethylether	17	-

**Table 3:** Isolated yield of major diastereoisomer of diethyl maleate cycloadduct **3a** from base screen reactions.

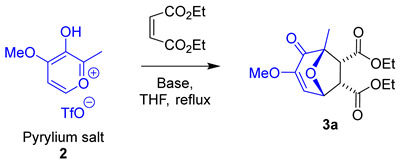				
Entry	Base	Equivalents	p*K*a	Yield 3a (%)
1	*N*,*N*-Dimethylaniline	1.5	5.2	17
2		3		14
3	2,6-Lutidine	1.5	6.7	-
4		3		-
5	Pyridine	1.5	5.3	-
6		3		23
7	*N*,*N*-Diisopropylethylamine	1.5	10.6	25
8		3		13
9	1,8-Diazabicyclo[5.4.0]undec-7-ene	1.5	12.5	-
10		3		-
11	4-(Dimethylamino)pyridine	1.5	9.7	34 ^†^
12		3		37 ^†^

^†^ Denotes ester hydrolysis

## Data Availability

The original contributions presented in this study are included in the article/Supplementary Materials. Further inquiries can be directed to the corresponding author. The X-ray structural data can be obtained from The Cambridge Crystallographic Data Centre (www.ccdc.cam.ac.uk/structures) as deposition numbers 2447755-2447756.

## Supplementary Materials

The following supporting information can be downloaded at: https://www.mdpi.com/article/10.3390/biom15060905/s1, General Experimental Information, Compound synthesis, Biological Studies, X-Ray Crystallography, NMR Images.
