# Supplementary material for: Cycloadditions as a Sweet Route to ‘Double C-Glycosylation’
Source: Biomolecules. 2025 Jun 19;15(6):905. doi: 10.3390/biom15060905 (PMC12190653; doi:10.3390/biom15060905)
Supplement: Supplementary file 1 [file biomolecules-15-00905-s001.zip › biomolecules-3671963-supplementary.pdf]

# Cycloadditions as a Sweet Route to 'Double *C*-Glycosylation'

**Kevin P. P. Mahoney*[1,2,3], Rosemary Lynch,[1] Rhea T. Bown[1], Sunil V. Sharma[1], Piyasiri Chueakwon[1], G. Richard Stephenson[3], David B. Cordes[4], Alexandra M. Z. Slawin[4] and Rebecca J. M. Goss*[1]**

**Contents**


| | |
|---|---|
| General Experimental Information | 1-2 |
| Compound Synthesis | 3-9 |
| Biological Studies | 9-11 |
| X-ray crystallography | 11 |
| References | 12 |
| NMR Images | 13-44 |


**Experimental Information**

## General Experimental Information

All reagents were purchased from commercial suppliers and were used without further purification unless otherwise stated. Dry solvents (dichloromethane, THF, toluene) were dried and deoxygenated with an MBraun SPS-800 solvent purification system and the moisture content of the solvent was analysed using a Karl Fischer coulometer (Metler Toledo DL32).

Biological reagents and components for media, buffers and stock solutions were purchased from commercial suppliers, used without further purification and stored according to the supplier's instructions. Microorganisms were stored at -80 °C for long and short-term storage. Microorganisms were cultured under sterile conditions using a Faster BH-EN class II vertical laminar airflow cabinet and fermentation media was sterilised at 121 °C for 20 minutes at 1.3 bar in a Boxer Benchtop Denley autoclave prior to use. Alternatively, aqueous solutions of heat labile components were sterilised by passage through a 0.2 µm membrane. Culturing apparatus was sterilised by autoclaving as described above or alternatively, disposable pre-sterilised apparatus was used.

General apparatus: Pipetting of solutions and samples was done using LABNET BioPette autoclavable pipettes. Microbial cultures were incubated in a New Brunswick Scientific Innova 4300 incubator shaker, a New Brunswick Scientific I26 incubator shaker series, or Genlab incubator (static). pH measurements were taken using a Fisherbrand Hydrus 300 pH meter. Centrifugation was carried out using a Thermo Scientific IEC CL30R centrifuge.

Proton NMR ($^{1}H$) and carbon NMR ($^{13}C$), were recorded on a Bruker Ascend 500 (500 MHz), Bruker 500 UltraShield (500 MHz), Bruker Ascend 700 (700 MHz), Bruker 400 UltraShield (400 MHz) or a Bruker UltraShield (300 MHz) spectrometer. Using an HSQC experiment with

multiplicity editing, the $^{13}C$ NMR signals were assigned to $CH_3$, $CH_2$, CH and C. The NMR experiments were carried out in deuterated chloroform ($CDCl_3$), deuterated acetonitrile ($CD_3CN$), deuterated water ($D_2O$), or deuterated DMSO (DMSO-$d_6$). The chemical shifts (δ) are quoted in parts per million (ppm). Multiplicities are abbreviated as s, singlet; d, doublet; t, triplet; q, quartet; m, multiplet; b, broad for the $^{1}H$ NMR and $^{13}C$ NMR spectra. Coupling constants are reported in Hertz (Hz).

Flash chromatography was performed using Davisil silica gel LC60A (40-63 micron). Thin layer chromatography (TLC) was performed using aluminium sheets of silica gel 60 F254 and was visualised under a Mineralight model UVGL-58 lamp (254 nm). The plates were developed with acidic methanolic vanillin solutions, ethanolic phosphomolybdic acid solutions or basic potassium permanganate solutions.

Microwave reactions were carried out in sealed vials using a Biotage Initiator-Plus.

Purification was carried out on a Biotage Isolera Four using SNAP KP-Sil 10 g column cartridges. The collection wavelength was set at 254 nm.

HPLC purification was carried out on an XBridge Prep Phenyl 5 µm column (10 x 250 mm) on a Gilson HPLC system (Gilson 322 pump, Gilson UV/Vis-151 detector, Gilson 402 syringe pump).

UPLC analysis was carried out on a Waters Acquity H-Class UPLC system, using a Waters ACQUITY UPLC BEH C18 1.7µm 2.1x50mm column and a gradient 10%-95% acetonitrile in 0.1% TFA in water.

High and low resolution mass spectra were recorded at the University of St Andrews on a Waters Micromass LCT time of flight mass spectrometer coupled to a Waters 2975 HPLC system or on an Orbitrap ELOS pro.

**Abbreviations**

THF – Tetrahydrofuran
DIPEA – Diisopropylethylamine
DMAD - Dimethyl acetylenedicarboxylate
TFA – Trifluoroacetic acid
NMR – Nuclear Magnetic Resonance

**Compound synthesis**

**General Protocol for Preparation of Cycloadducts**

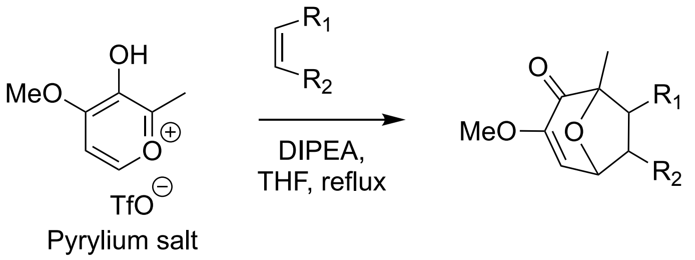


In an oven dried flask, to a solution of the pyrylium salt (0.1 g, 0.34 mmol, 1 equiv.) in dry THF (10 mL) under an atmosphere of argon, a dipolarophile (1.7 mmol, 5 equiv.) was added. DIPEA (0.09 mL, 0.51 mmol, 1.5 equiv.) was then added dropwise before heating to reflux. The reaction mixture was stirred under reflux for 24 hours, before cooling to room temperature. The solvent was removed by rotary evaporation under reduced pressure. The crude mixture was then loaded onto a silica column and first washed with 20% diethyl ether in hexane, before being eluted with 100% ether. The product was then purified using automated column chromatography using a gradient from 20% diethyl ether in hexane to 100% diethyl ether. Where diastereomers were identified these were separated by semi-prep reverse-phase HPLC.

Preparation of pyrylium salt, 3-hydroxy-4-methoxy-2-methylpyrylium trifluoromethanesulfonate (**2**)

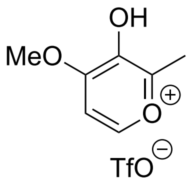


To a solution of maltol (5g, 39.6 mmol, 1.0 equiv.) in dichloromethane (10 mL) was added methyl trifluoromethanesulfonate (6.5 mL, 59 mmol, 1.5 equiv.). The reaction was stirred at reflux for 4 h, cooled to room temperature and then evaporated under reduced pressure to afford a white solid, which was recrystallised from ethyl acetate, 7.7g, 64% yield. NMR data was in agreement with literature[1]

$^{1}$H NMR (500 MHz, $CD_3CN$) δ 8.82 (d, $J$ = 5.2 Hz, 1H), 7.62 (d, $J$ = 5.2 Hz, 1H), 4.33 (s, 3H), 2.70 (s, 3H).

$^{13}$C NMR (126 MHz, $CD_3CN$) δ 168.9 (C), 166.5 (C), 161.2 (C), 143.1 (CH), 118.3 ($CF_3$), 108.6 (CH), 60.8 ($CH_3$), 16.4 ($CH_3$).

$^{19}$F NMR (471 MHz, $CD_3CN$) δ -79.4.

Diethyl 3-methoxy-5-methyl-4-oxo-8-oxabicyclo[3.2.1]oct-2-ene-6,7-dicarboxylate (**3a**)

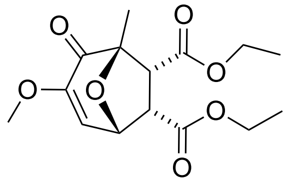

The above product was prepared following the General Protocol for Preparation of Cycloadducts using diethyl maleate (0.28 mL, 1.7 mmol, 5 equiv.). The major diastereomer of the reaction was obtained in 25% isolated yield (27 mg).

$^{1}$H NMR (400 MHz, $CDCl_3$) δ 6.32 (d, *J* = 5.2 Hz, 1H), 5.00 (dd, *J* = 6.1, 5.2 Hz, 1H), 4.14 – 4.04 (m, 2H), 3.99 (dd, *J* = 7.2, 4.8 Hz, 2H), 3.70 (dd, *J* = 11.2, 6.1 Hz, 1H), 3.64 (s, 3H), 3.42 (d, *J* = 11.2 Hz, 1H), 1.59 (s, 3H), 1.18 (t, *J* = 7.2 Hz, 3H), 1.16 (t, *J* = 7.1 Hz, 3H).

$^{13}$C NMR (101 MHz, $CDCl_3$) δ 191.4 (C=O), 169.4 (C=O), 169.1 (C=O), 150.8 (C), 115.9 (CH), 89.0 (C), 74.2 (CH), 61.8 ($CH_2$), 61.1 ($CH_2$), 55.4 ($OCH_3$), 54.1 (CH), 52.5 (CH), 20.5 ($CH_3$), 14.2 ($CH_3$), 14.0 ($CH_3$).

HRMS (ES+) m/z 313.1285 ([M + H]$^+$, 100%) calculated for $C_{15}H_{20}O_7$ [M + H]$^+$ 313.1282;

IR $v_{max}$/cm$^{-1}$ 1748, 1734, 1699, 1684, 1558, 1506.

Diethyl 3-methoxy-5-methyl-4-oxo-8-oxabicyclo[3.2.1]oct-2-ene-6,7-dicarboxylate (second isomer) (**3b**)

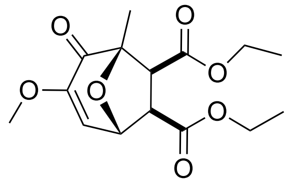

The above product was prepared following the General Protocol for Preparation of Cycloadducts using diethyl maleate (0.28 mL, 1.7 mmol, 5 equiv.). The minor diastereomer of the reaction was obtained in 11% isolated yield (12 mg).

$^{1}$H NMR (400 MHz, $CDCl_3$) δ 6.20 (d, *J* = 5.6 Hz, 1H), 5.44 (d, *J* = 5.6 Hz, 1H), 4.23 – 4.07 (m, 4H), 3.62 (s, 3H), 3.28 (s, 2H), 1.51 (s, 3H), 1.28 (t, *J* = 7.1 Hz, 3H), 1.23 (t, *J* = 7.2 Hz, 3H).

$^{13}$C NMR (101 MHz, $CDCl_3$) δ 192.1 (C=O), 170.2 (C=O), 169.5 (C=O), 149.7 (C), 118.2 (CH), 89.2 (C), 73.9 (CH), 62.2 ($CH_2$), 61.5 ($CH_2$), 55.3 ($OCH_3$), 53.7 (CH), 49.7 (CH), 16.5 ($CH_3$), 14.2 ($CH_3$), 14.1 ($CH_3$). * taken from 2D spectra

HRMS (ES+) m/z 313.1285 ([M + H]$^+$, 100%) calculated for $C_{15}H_{20}O_7$ [M + H]$^+$ 313.1287;

Dimethyl 3-methoxy-5-methyl-4-oxo-8-oxabicyclo[3.2.1]octa-2,6-diene-6,7-dicarboxylate (**4**)

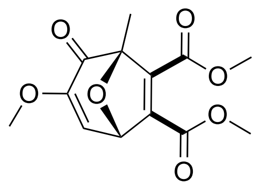

The above product was prepared following the General Protocol for Preparation of Cycloadducts using DMAD (0.42 mL, 1.7 mmol, 5 equiv.). A single product was isolated in 44% isolated yield (42 mg).

$^{1}$H NMR (500 MHz, DMSO-$d_6$) δ 6.48 (d, $J$ = 5.0 Hz, 1H), 5.56 (d, $J$ = 5.0 Hz, 1H), 3.76 (s, 3H), 3.74 (s, 3H), 3.51 (s, 3H), 1.48 (s, 3H).

$^{13}$C NMR* (126 MHz, DMSO-$d_6$) δ 187.9 (C=O), 162.7 (C=O), 161.7 (C=O), 145.2 (C), 141.4 (C), 115.9 (CH), 92.9 (C), 77.6 (C), 77.4 (CH), 54.8 ($OCH_3$), 52.6 ($OCH_3$), 52.3 ($OCH_3$), 15.8 ($CH_3$).
* taken from 2D (HSQC and HMBC) spectra

HRMS (ES+) m/z 283.0811 ([M + H]$^+$, 100%) calculated for $C_{13}H_{15}O_7$ [M + H]$^+$ 283.0818;

$v_{max}$/cm$^{-1}$ 1717, 1558, 1506, 1068.

(3-Methoxy-1-methyl-2-oxo-7-phenyl-8-oxabicyclo[3.2.1]oct-3-en-6-yl)methyl acetate (**5**)

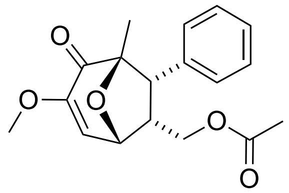

The above product was prepared as follows: Cinnamyl acetate (0.7 mL; 4 mmol, 1.4 equiv.) and *N,N*-dimethylaniline (1 mL; 8 mmol, 2.8 equiv.) were heated with stirring to 50 °C. To this mixture was added pyrylium salt (0.867 g; 2.9 mmol, 1 equiv.) portion wise over 15 minutes. The reaction was then heated to 105 °C for 18 hours and the product then purified by column chromatography with 25:1 dichloromethane : ethyl acetate to yield product 3 as an orange oil 8% yield (73 mg).

$^{1}$H NMR (500 MHz, $CDCl_3$) δ 7.29 – 7.21 (m, 3H), 7.08 – 7.00 (m, 2H), 6.32 (d, $J$ = 5.5 Hz, 1H), 4.79 (d, $J$ = 5.5 Hz, 1H), 4.26 (dd, $J$ = 10.8, 6.0 Hz, 1H), 4.19 (dd, $J$ = 10.8, 9.0 Hz, 1H), 3.70 (s, 3H), 2.95 (d, 5.0 Hz, 1H), 2.86 (m, 1H), 2.02 (s, 3H), 1.54 (s, 3H).

$^{13}$C NMR* (126 MHz, $CDCl_3$) δ 191.9 (C=O), 170.8 (C=O), 151.3 (C), 135.6 (C), 128.7 (CH), 128.2 (CH), 127.7 (CH), 117.7 (CH), 91.1 (C), 74.7 (CH), 66.4 ($CH_2$), 56.3 (CH), 55.3 ($OCH_3$), 51.3 (CH), 20.8 ($CH_3$), 20.0 ($CH_3$).
* taken from 2D (HSQC and HMBC) spectra

TOF MS (ESI+): m/z 339.11 ([M+Na]+, 100%) calculated for $C_{18}H_{20}O_5$ [M + $Na^+$] 339.1208.

(1*R*,2*S*,6*S*,7*R*)-6,9-Dimethoxy-1,2-dimethyl-3,11-dioxatricyclo[5.3.1.12,6]dodeca-4,8-diene-10,12-dione (**6**)

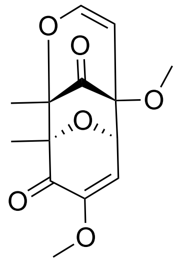

Following the general procedure, pyrylium salt dimer was isolated as a by-product from several cycloaddition reactions. NMR data, and our obtained crystal structure (CCDC 2447755) was in agreement with literature [1].

$^{1}$H NMR (500 MHz, $CDCl_3$) δ 6.66 (d, *J* = 5.9 Hz, 1H), 6.00 (d, *J* = 5.1 Hz, 1H), 5.01 (d, *J* = 5.9 Hz, 1H), 4.48 (d, *J* = 5.1 Hz, 1H), 3.62 (s, 3H), 3.48 (s, 3H), 1.55 (s, 3H), 1.24 (s, 3H).

$^{13}$C NMR (126 MHz, $CDCl_3$) δ 199.5 (C=O), 188.8 (C=O), 149.9 (C), 148.2 (C), 113.0 (C), 100.5 (C), 92.4 (CH), 87.6 (CH), 86.3 (CH), 77.4 (CH), 55.5 ($OCH_3$), 54.1 ($OCH_3$), 17.3 ($CH_3$), 14.7 ($CH_3$).

HRMS (ES+) m/z 281.1021 ($[M + H]^+$, 100%) calculated for $C_{14}H_{17}O_6$ $[M + H]^+$ 281.1020.

3-Methoxy-1-methyl-6-phenyl-8-oxabicyclo[3.2.1]oct-3-en-2-one (**7**)

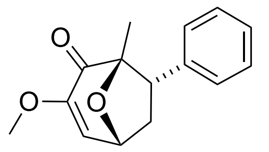

The above product was prepared following the General Protocol for Preparation of Cycloadducts using styrene (0.2 mL, 1.7 mmol, 5 equiv.). A single product was isolated in 32% isolated yield (28 mg).

$^{1}$H NMR (500 MHz, DMSO-*d*$_6$) δ 7.26 – 7.20 (m, 2H), 7.20 – 7.15 (m, 1H), 7.08 – 7.04 (m, 2H), 6.54 (d, *J* = 5.3 Hz, 1H), 5.00 (dd, *J* = 6.8, 5.3 Hz, 1H), 3.56 (s, 3H), 3.46 (dd, *J* = 11.0, 4.8 Hz, 1H), 2.75 (ddd, *J* = 12.4, 11.0, 6.8 Hz, 1H), 2.10 (dd, *J* = 12.4, 4.8 Hz, 1H), 1.37 (s, 3H).

$^{13}$C NMR (176 MHz, $CDCl_3$) δ 193.9 (C=O), 150.1 (C), 138.3 (C), 128.6 (CH), 128.4 (CH), 127.3 (CH), 115.8 (CH), 86.7 (C), 77.8 (CH), 55.4 ($OCH_3$), 48.9 (CH), 37.7 ($CH_2$), 19.9 ($CH_3$).

HRMS (ES+) m/z 245.1182 ($[M + H]^+$, 100%) calculated for $C_{15}H_{17}O_3$ $[M + H]^+$ 245.1178;

$v_{max}/cm^{-1}$ 2365, 1699, 1559, 1506, 1069.

3-Methoxy-1-methyl-2-oxo-7-phenyl-8-oxabicyclo[3.2.1]oct-3-ene-6-carboxylic acid (**8**)

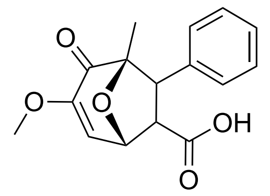

The above product was prepared following the General Protocol for Preparation of Cycloadducts using methyl cinnamate (0.28g, 1.7 mmol, 5 equiv.), followed by purification by column chromatography using a gradient from 20% ethyl acetate in hexane to 100% ethyl acetate. Instead of expected methyl ester, the isolated product was identified as the hydrolysis product, carboxylic acid. Red oil in 32% yield (22.2 mg).

$^{1}$H NMR (500 MHz, DMSO-$d_6$) δ 7.53 – 7.44 (m, 2H), 7.45 – 7.38 (m, 1H), 7.02 – 6.95 (m, 2H), 6.33 (d, $J$ = 5.3 Hz, 1H), 5.37 (dd, $J$ = 7.3, 5.3 Hz, 1H), 4.17 (dd, $J$ = 8.9, 7.3 Hz, 1H), 3.85 (d, $J$ = 8.9 Hz, 1H), 3.51 (s, 3H), 1.56 (s, 3H).

$^{13}$C NMR (126 MHz, DMSO-$d_6$) δ 189.1 (C=O), 173.3 (C=O), 150.3 (C), 131.6 (C), 129.2 (CH), 128.7 (CH), 126.5 (CH), 115.4 (CH), 87.7 (C), 73.2 (CH), 55.3 ($CH_3$), 52.4 (CH), 51.0(CH), 20.3 ($CH_3$).

8-Methoxy-6-methyl-5a,6,10,10a-tetrahydro-5*H*-6,10-epoxycyclohepta[*b*]naphthalene-5,7,11-trione (**9a**)

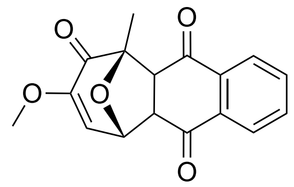

The above product was prepared following the General Protocol for Preparation of Cycloadducts using 1,4-naphthoquinone (0.27 g, 1.7 mmol, 5 equiv.), followed by twice column purification on silica using a gradient from 20% ethyl acetate in hexane to 100% ethyl acetate, giving the product in 41% yield (41 mg).

$^{1}$H NMR (500 MHz, $CDCl_3$) δ 8.21 – 7.95 (m, 2H), 7.84 – 7.76 (m, 2H), 6.14 (d, $J$ = 5.3 Hz, 1H), 5.48 (d, $J$ = 5.3 Hz, 1H), 3.67 (s, 3H), 3.50 (d, $J$ = 8.4 Hz, 1H), 3.47 (d, $J$ = 8.4 Hz, 1H), 1.30 (s, 3H).

$^{13}$C NMR (126 MHz, $CDCl_3$) δ 194.3 (C=O), 193.7 (C=O), 191.0 (C=O), 149.3 (C), 136.1 (C), 135.3 (CH), 135.0 (CH), 134.5 (C), 127.6 (CH), 127.0 (CH), 115.0 (CH), 89.0 (C), 79.5 (CH), 55.5 ($CH_3$), 54.2 (CH), 51.4 (CH), 16.6 ($CH_3$).

HRMS (ES+) m/z 299.0912 ($[M + H]^+$, 100%) calculated for $C_{17}H_{15}O_5$ $[M + H]^+$ 299.0919;

$\nu_{max}/cm^{-1}$ 1664, 1663, 1591, 1288, 1259.

1-Acetyl-2-methoxyanthracene-9,10-dione (**9b**)

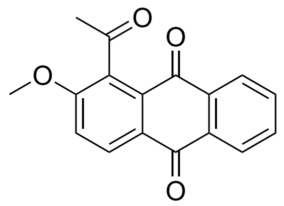

The above product was prepared as follows: 1,4-Naphthoquinone (0.401 g; 3 mmol, 3.75 equiv.) and *N,N*-dimethylaniline (0.46 mL; 4 mmol, 5 equiv.) were heated with stirring to 50 °C. To this mixture was added pyrylium salt (0.250 g; 0.8 mmol, 1 equiv.) portion wise over 15 minutes. The reaction was then heated to 70 °C for 18 hours and the product then purified by column chromatography with 25:1 dichloromethane : ethyl acetate to yield a yellow crystalline solid in 16% yield (38 mg). The structure was confirmed by x-ray crystallography (CCDC 2447756)

$^{1}$H NMR (500 MHz, $CDCl_3$) δ 8.38 (d, *J* = 8.7 Hz, 1H), 8.30 (dd, *J* = 7.6, 1.5 Hz, 1H), 8.22 (dd, *J* = 7.6, 1.5 Hz, 1H), 7.86 – 7.73 (m, 2H), 7.33 (d, *J* = 8.7 Hz, 1H), 3.98 (s, 3H), 2.64 (s, 3H).

$^{13}$C NMR (126 MHz, $CDCl_3$) δ 203.1 (C=O), 183.3 (C=O), 181.9 (C=O), 160.0 (C), 134.5 (C), 134.3 (CH), 133.8 (C), 132.4 (C), 131.6 (C), 130.5 (CH), 127.4 (CH), 127.2 (CH), 126.7 (C), 116.1 (CH), 56.4 ($CH_3$), 31.1 ($CH_3$).

HRMS (ES+) m/z 281.0808 ($[M + H]^+$, 100%) calculated for $C_{17}H_{13}O_4$ $[M + H]^+$ 281.0814.

These data are consistent, with the data previously reported for this compound[2].

6-Methoxy-4-methyl-3a,4,8,8a-tetrahydro-4,8-epoxycyclohepta[c]pyrrole-1,3,5(2H)-trione (**10**)

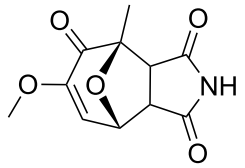

The above product was prepared following the General Protocol for Preparation of Cycloadducts using maleimide (0.17 g, 1.7 mmol, 5 equiv.), but using DMAP (0.06 g, 0.51 mmol, 1.5 equiv.) as base and purification by silica chromatography using a gradient of 20% ethyl acetate in hexane to 100% ethyl acetate to afford a white solid 53% yield (42.8 mg).

$^{1}$H NMR (500 MHz, DMSO-$d_6$) δ 11.55 (s, 1H), 6.50 (d, *J* = 5.4 Hz, 1H), 5.06 (d, *J* = 5.4 Hz, 1H), 3.56 (d, *J* = 7.2 Hz, 4H), 3.53 (s, 3H), 3.08 (d, *J* = 7.2 Hz, 1H), 1.33 (s, 3H).

$^{13}$C NMR (176 MHz, DMSO-$d_6$) δ 191.4 (C=O), 177.5 (C=O), 175.2 (C=O), 148.3 (C), 118.9 (CH), 87.1 (C), 74.6 (CH), 54.9 ($CH_3$), 54.6 (CH), 49.0 (CH), 16.8 ($CH_3$).

HRMS (ES+) m/z 238.0711 ($[M + H]^+$, 100%) calculated for $C_{11}H_{12}NO_5$ $[M + H]^+$ 238.0715;

$\nu_{max}/cm^{-1}$ 3294, 1717, 1636, 1362, 1157.

6-Methoxy-4-methyl-2-phenyl-3a,4,8,8a-tetrahydro-4,8-epoxycyclohepta[*c*]pyrrole-1,3,5(2*H*)-trione (**11**)

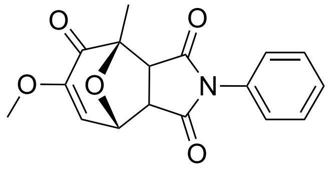


The above product was prepared following the General Protocol for Preparation of Cycloadducts using *N*-phenylmaleimide (0.29 g, 1.7 mmol, 5 equiv.), followed by purification by column chromatography using a gradient from 20% ethyl acetate in hexane to 100% ethyl acetate to afford the product as a white solid in 40% yield (42 mg)

$^{1}$H NMR (700 MHz, DMSO-$d_6$) δ 7.49 (dd, *J* = 8.5, 7.1 Hz, 2H), 7.45 – 7.39 (m, 1H), 7.01 – 6.97 (m, 2H), 6.33 (d, *J* = 5.2 Hz, 1H), 5.37 (dd, *J* = 7.3, 5.2 Hz, 1H), 4.17 (dd, *J* = 8.9, 7.3 Hz, 1H), 3.85 (d, *J* = 8.9 Hz, 1H), 3.51 (s, 3H), 1.56 (s, 3H).

$^{13}$C NMR (176 MHz, DMSO-$d_6$) δ 189.6 (C=O), 173.8 (C=O), 173.5 (C=O), 150.7 (C), 132.1 (C), 129.6 (CH), 129.2 (CH), 127.0 (CH), 115.8 (CH), 88.2 (C), 73.7 (CH), 55.8 ($CH_3$), 52.9 (CH), 51.5 (CH), 20.7 ($CH_3$).

HRMS (ES+) m/z 314.1024 ([M + H]$^+$, 100%) calculated for $C_{17}H_{16}NO_5$ [M + H]$^+$ 314.1028;

$\nu_{max}$/cm$^{-1}$ 1705, 1506, 1381, 1143.

**Biological studies**

**Antibacterial activity tests**

*E. coli* ATCC 25922, *P. aeruginosa* ATCC 27853, and *S. aureus* ATCC 25923 were cultured in Mueller-Hinton broth (MHB) at 37 °C, 200 rpm overnight. The bacterial cells were harvested and washed three times with 0.85% NaCl to remove residual medium. The cell suspensions were then adjusted with 0.85% NaCl to a final concentration of 1 × $10^6$ to 1 × $10^7$ CFU/mL. A volume of 20 µL of each cell suspension was added to a 96-well plate containing 160 µL of MHB and treated with 20 µL of **10** at final concentrations of 0, 2, 4, 8, 16, 32, 64, 128, 256, 512, and 1024 µM. The final concentration of DMSO in all treatments was maintained at 1%. The treated cells were incubated at 37 °C overnight. Cell viability was assessed by measuring the optical density at 600 nm ($OD_{600}$). The minimum inhibitory concentration (MIC) was defined as the lowest compound concentration that inhibited visible bacterial growth. To determine the minimum bactericidal concentration (MBC), 10 µL from wells at the MIC and higher concentrations were spotted onto Mueller-Hinton agar (MHA) plates and incubated at 37 °C for 18 h. The MBC was defined as the lowest concentration at which no bacterial growth was observed.

**Compound 10**

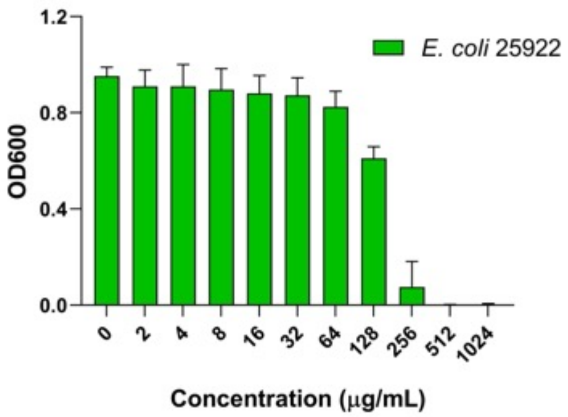


**Compound 10**

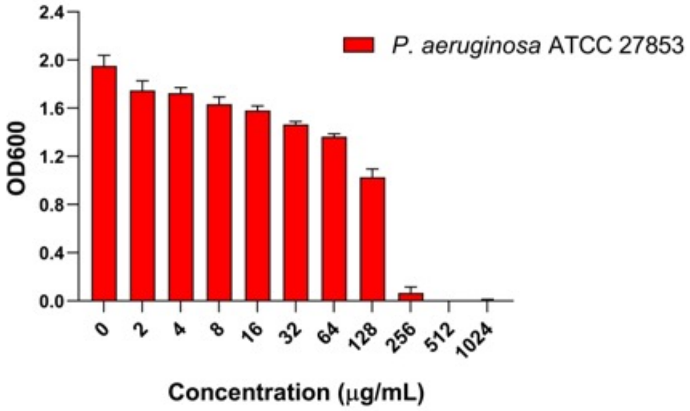


**Compound 10**

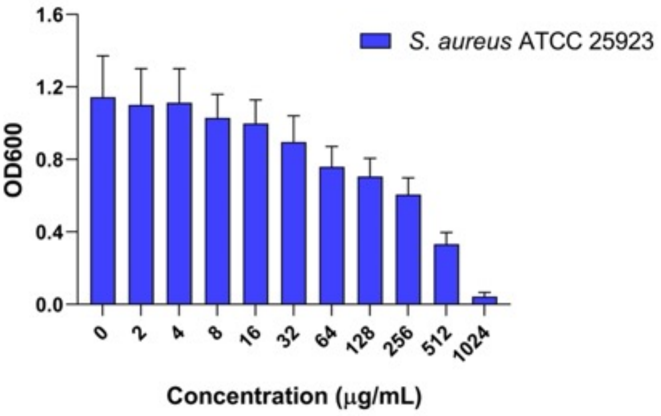

| Bacterial Isolates | **10** (µg/mL) | | Ampicillin (µg/mL) | |
|---|---|---|---|---|
| | MIC | MBC | MIC | MBC |
| *E. coli* 25922 | 512 | 1024 | 4 | 8 |
| *S. aureus* ATCC 25923 | >1024 | >1024 | 2 | 2 |
| *P. aeruginosa* ATCC 27853 | 512 | 1024 | 512 | >1024 |

* Data are expressed as mean ± SD from three independent biological experiments.

## X-ray Experimental data

| | **6** | **9b** |
|---|---|---|
| formula | $C_{14}H_{16}O_6$ | $C_{17}H_{12}O_4$ |
| fw | 280.27 | 280.27 |
| crystal description | Colourless plate | Yellow prism |
| crystal size [mm$^3$] | 0.24×0.18×0.02 | 0.12×0.09×0.05 |
| space group | $P\bar{1}$ | $P\bar{1}$ |
| $a$ [Å] | 9.2341(3) | 7.884(3) |
| $b$ [Å] | 10.6447(5) | 8.055(3) |
| $c$ [Å] | 14.4081(6) | 10.842(3) |
| $\alpha$ [°] | 77.390(4) | 92.509(17) |
| $\beta$ [°] | 74.942(4) | 108.668(19) |
| $\gamma$ [°] | 89.491(3) | 95.313(13) |
| vol [Å]$^3$ | 1332.89(10) | 647.5(4) |
| $Z$ | 4 | 2 |
| $\rho$ (calc) [g/cm$^3$] | 1.397 | 1.437 |
| $\mu$ [mm$^{-1}$] | 0.928 | 0.850 |
| F(000) | 592 | 292 |
| reflections collected | 12704 | 6785 |
| independent reflections ($R_{int}$) | 4770 (0.0308) | 2308 (0.0385) |
| parameters, restraints | 369, 0 | 193, 0 |
| GoF on $F^2$ | 1.072 | 1.129 |
| $R_1$ [$I > 2\sigma(I)$] | 0.0428 | 0.0644 |
| $wR_2$ (all data) | 0.1270 | 0.1358 |
| largest diff. peak/hole [e/Å$^3$] | 0.220, -0.241 | 0.512, -0.6620 |

Further details can be found with reference CCDC 2447755-2447756, free of charge, from The Cambridge Crystallographic Data Centre via www.ccdc.cam.ac.uk/structures.

References

1. Bejcek, L. P.; Garimallaprabhakaran, A. K.; Suyabatmaz, D. M.; Greer, A.; Hersh, W. H.; Greer, E. M.; Murelli, R. P., Maltol- and Allomaltol-Derived Oxidopyrylium Ylides: Methyl Substitution Pattern Kinetically Influences [5 + 3] Dimerization versus [5 + 2] Cycloaddition Reactions. *The Journal of Organic Chemistry* **2019,** 84, (22), 14670-14678.
2. Shah, N. H.; Sethna, S., Hydroxyanthracene Series Part IV. Synthesis of Some Anthraquinone Derivatives. *Journal of the Chemical Society* **1961**, 4682-4684.

(**2**)

3-Hydroxy-4-methoxy-2-methylpyrylium trifluoromethanesulfonate (**2**)

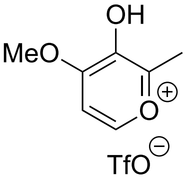


2205101546-0-24-svs4.10.fid — svs-4x2 Pyryrlium-OTf crystals crop-1 || 1H Observe

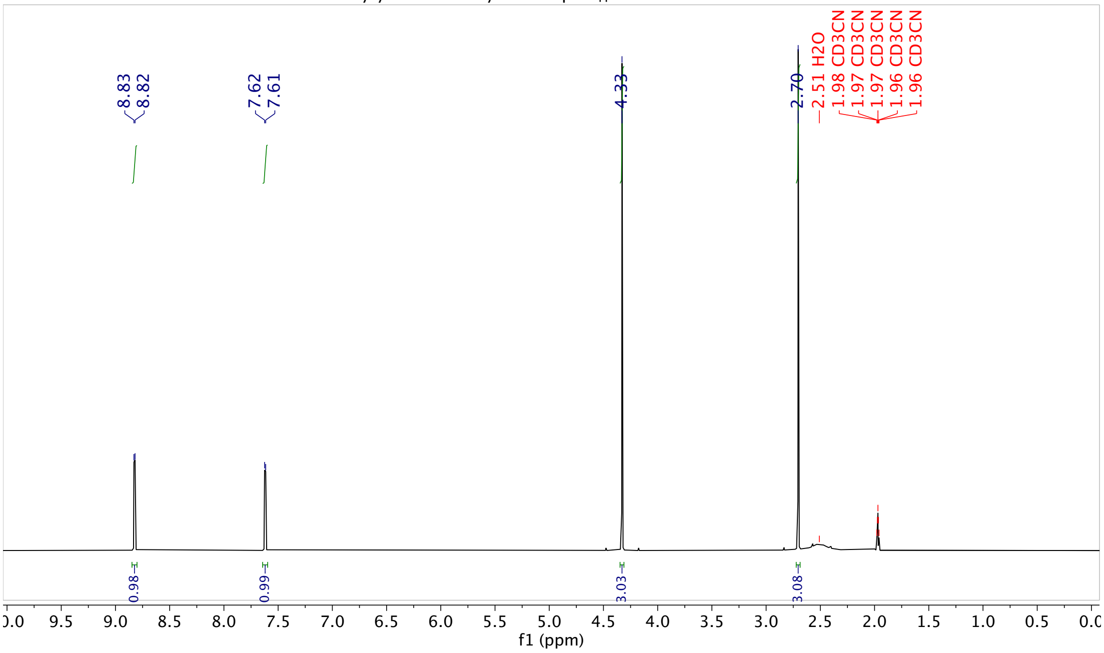


2205101546-0-24-svs4.11.fid — svs-4x2 Pyryrlium-OTf crystals crop-1 || 13C Observe with multiplicity editing - DEPTQ

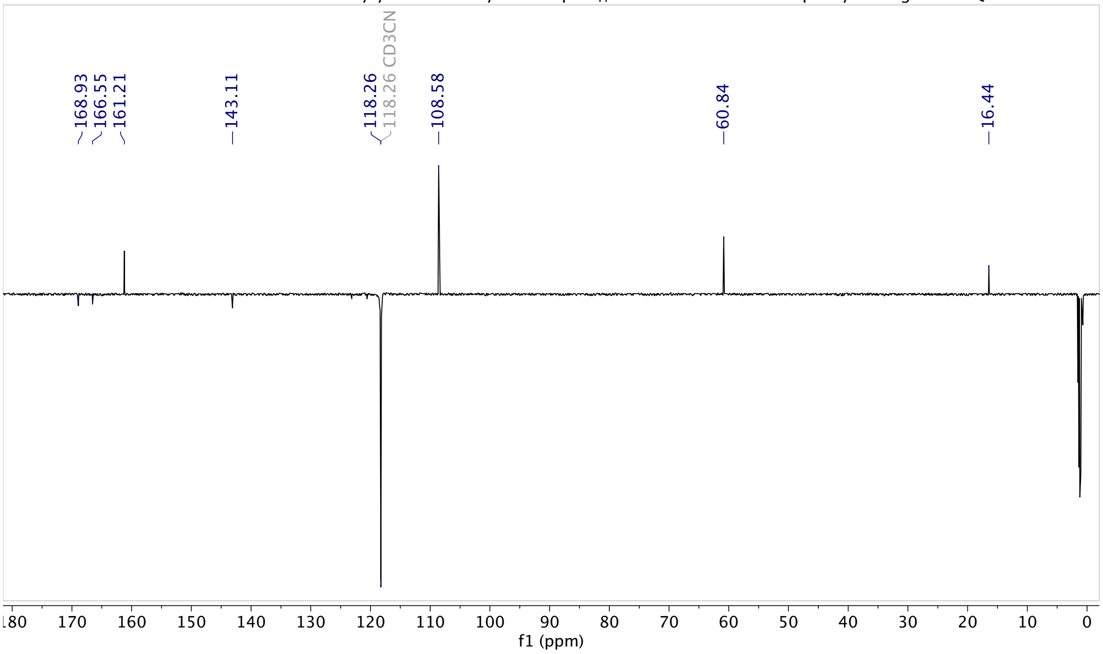

**(2, & 3a)**

2205101546-0-24-svs4.12.fid — svs-4x2 Pyrylium-OTf crystals crop-1 || 19F Observe without 1H decoupling - Full Range SW

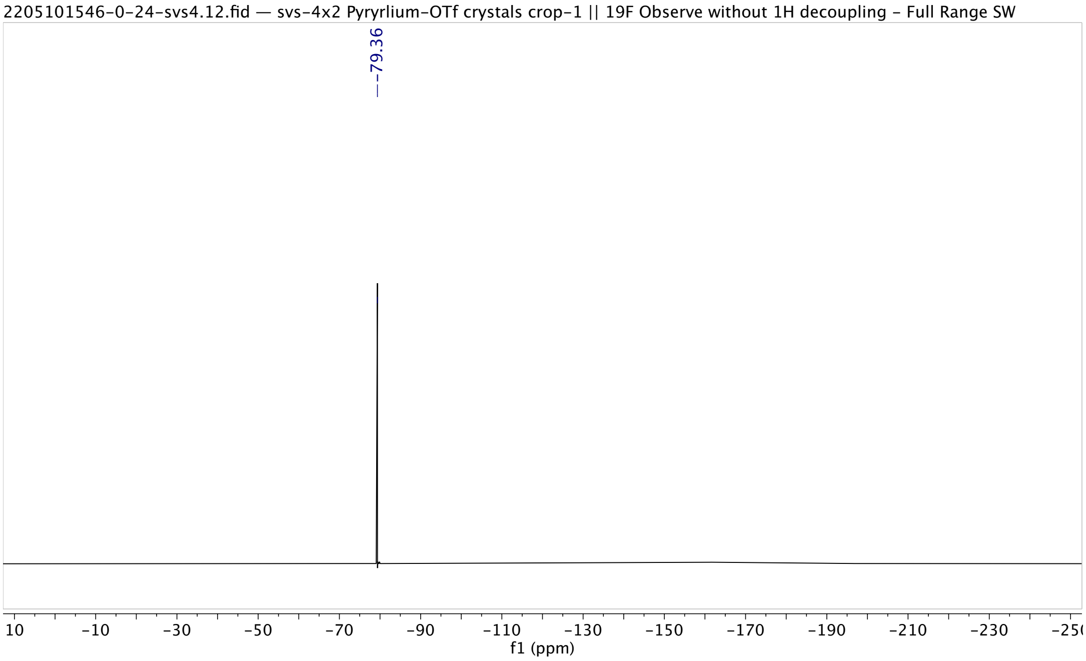


Diethyl 3-methoxy-5-methyl-4-oxo-8-oxabicyclo[3.2.1]oct-2-ene-6,7-dicarboxylate (**3a**)

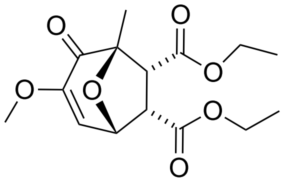

10202014-6-rjmg-rtb4-N.10.fid — 1H Observe — RTB10

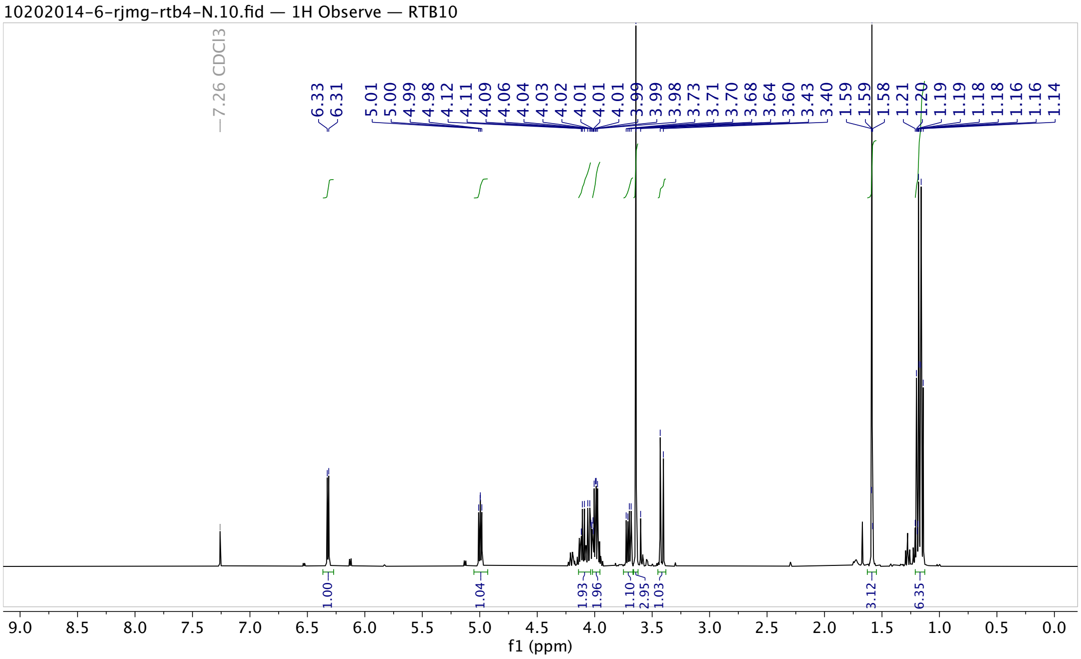

**(3a)**

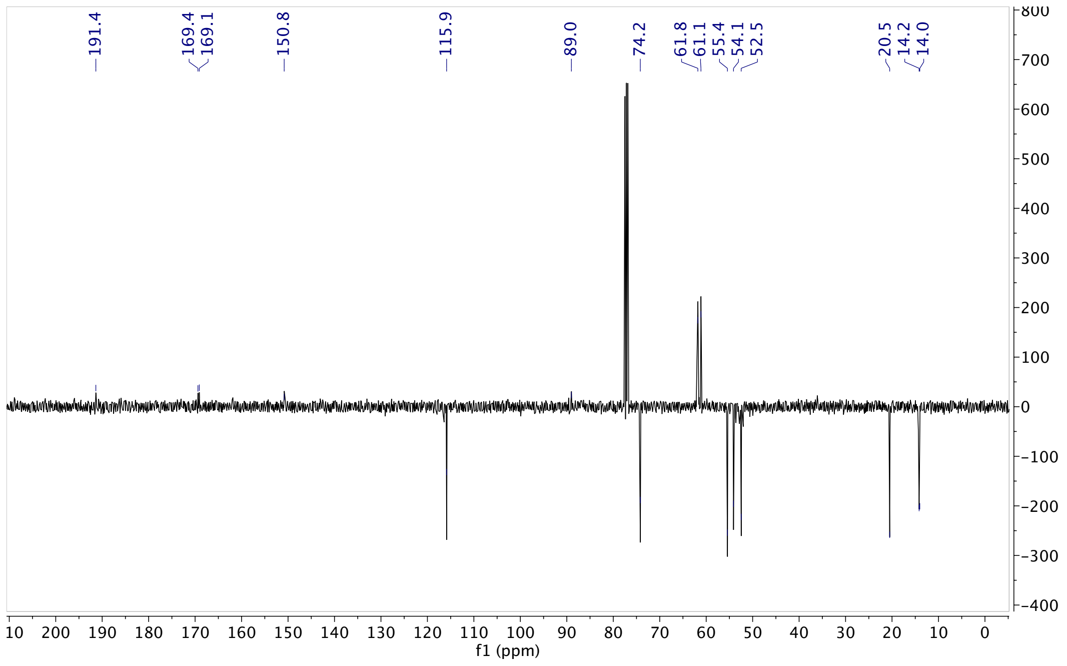

191.4
169.4
169.1
150.8
115.9
89.0
74.2
61.8
61.1
55.4
54.1
52.5
20.5
14.2
14.0
f1 (ppm)


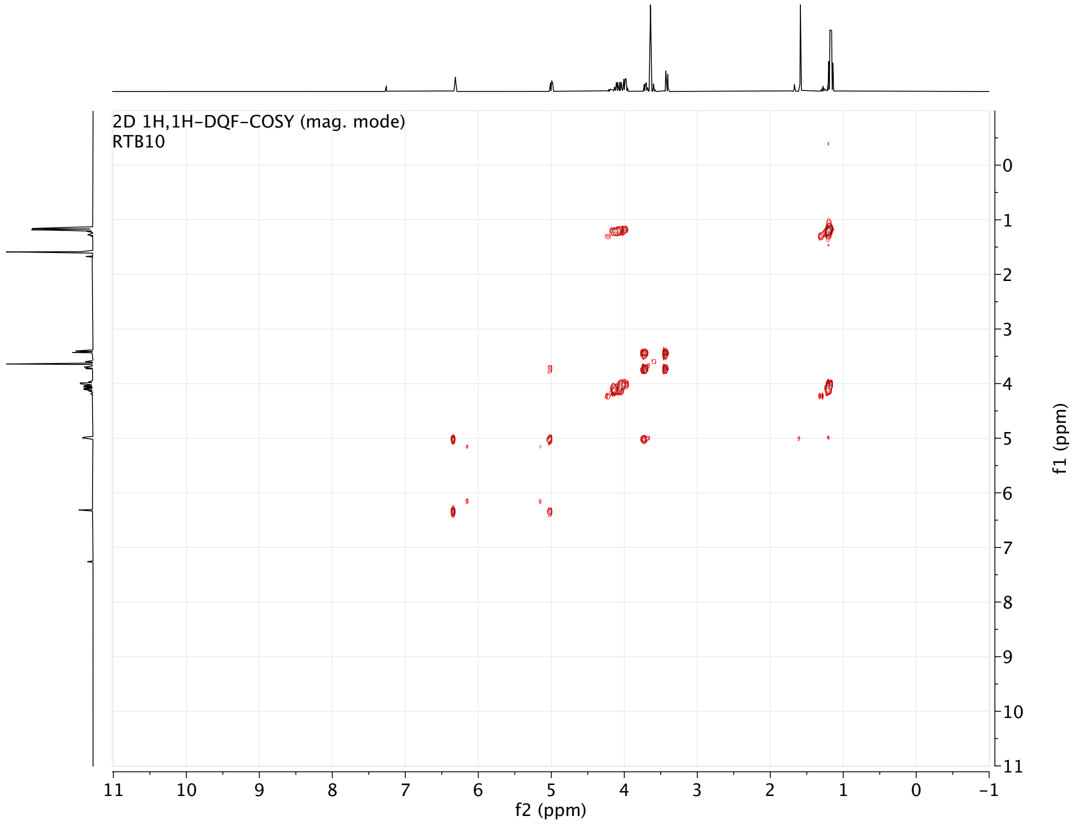

2D 1H,1H-DQF-COSY (mag. mode)
RTB10
f2 (ppm)
f1 (ppm)

**(3a)**

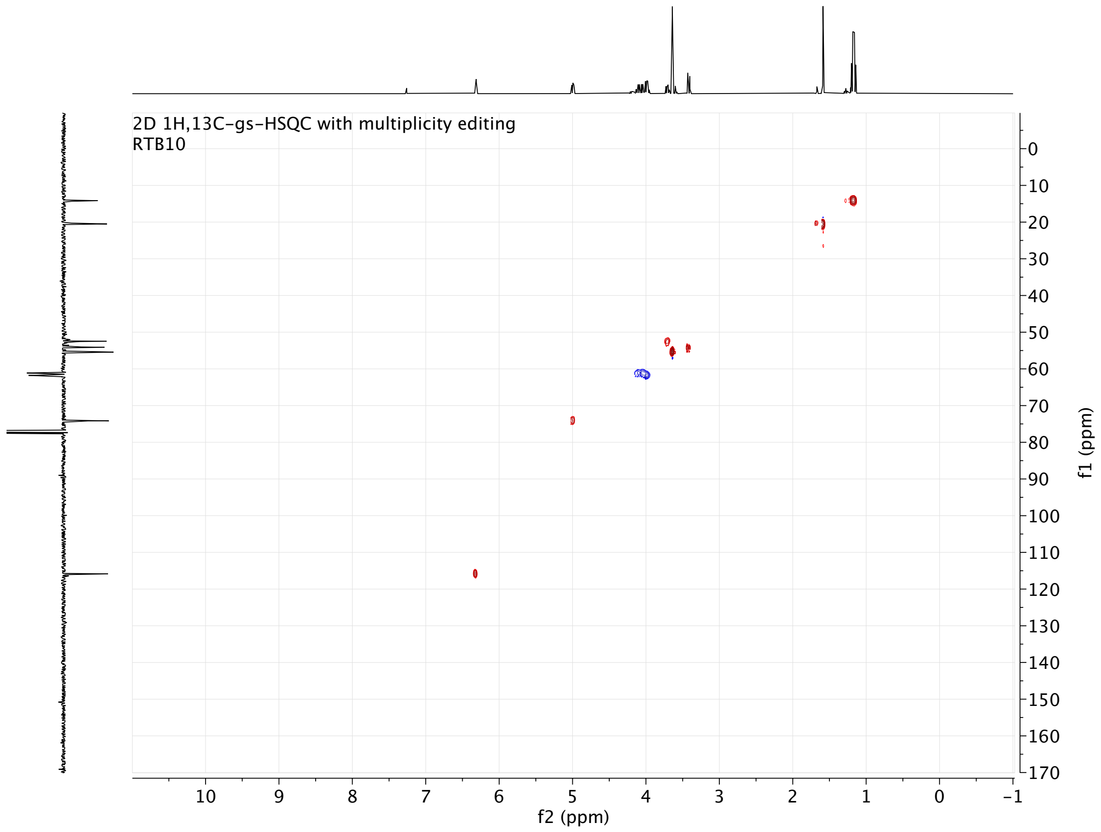
2D 1H,13C-gs-HSQC with multiplicity editing
RTB10
f2 (ppm)
f1 (ppm)

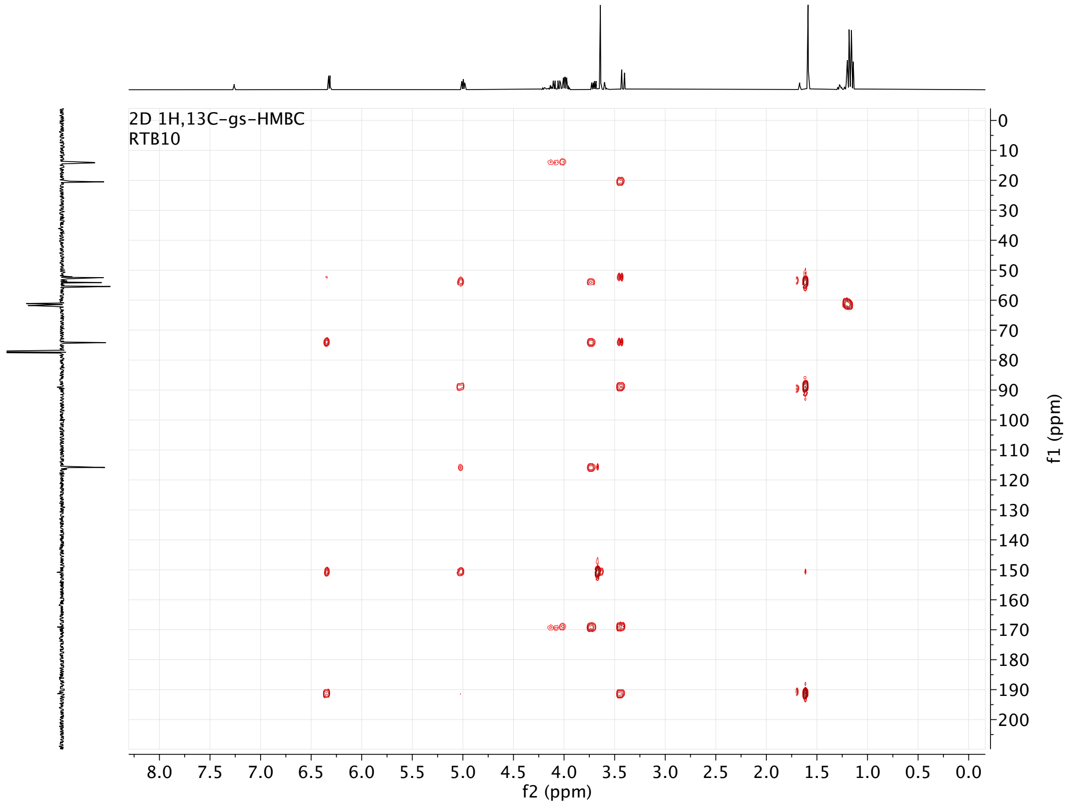
2D 1H,13C-gs-HMBC
RTB10
f2 (ppm)
f1 (ppm)

**(3a)**

NOE relationships confirm syn structure

$H_1$ – 6.32 ppm
$H_2$ – 5.00 ppm
$H_3$ – 3.70 ppm
$H_4$ – 3.42 ppm

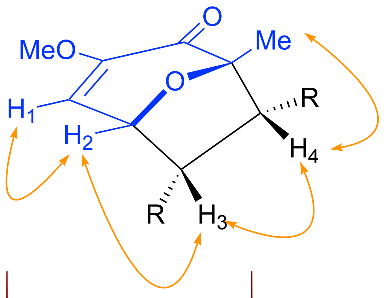


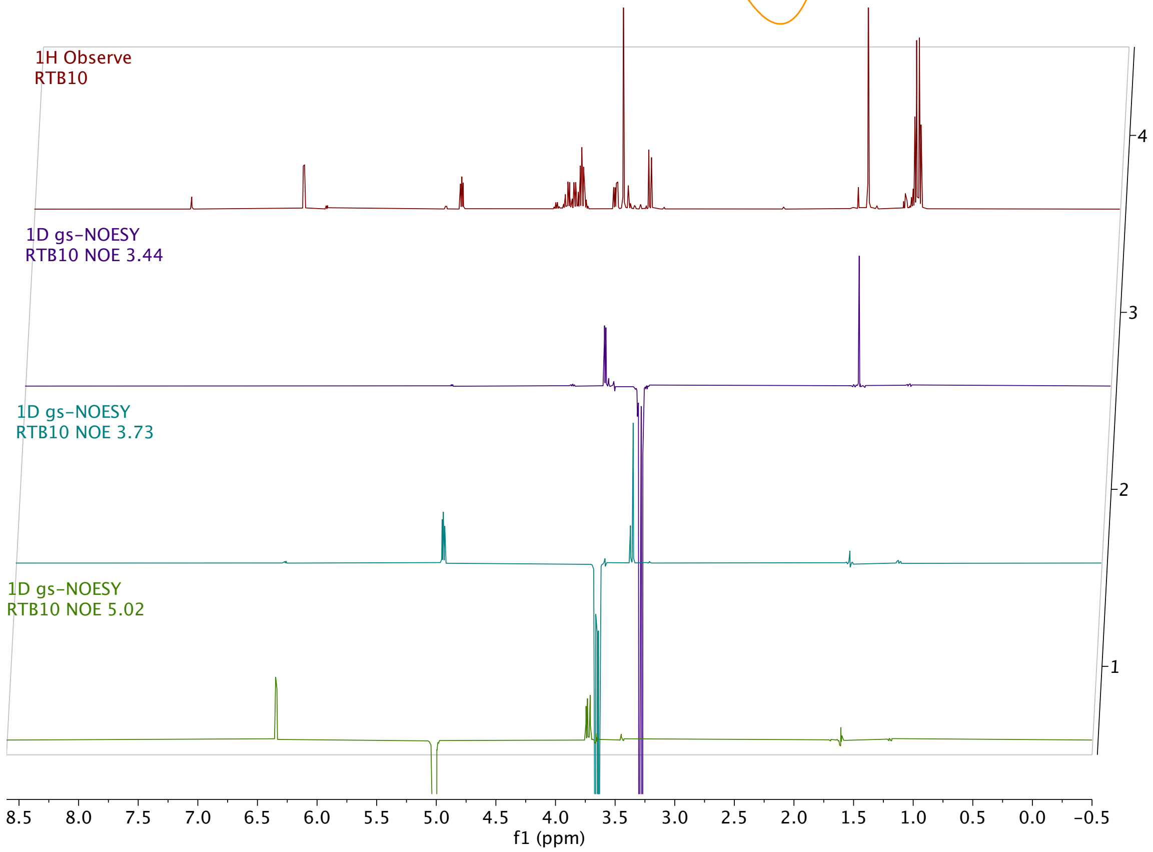

**(3b)**

**(3b)**

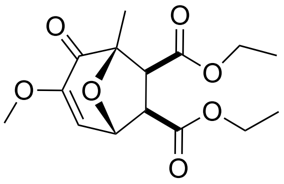

10202014-7-rjmg-rtb4-N.10.fid — 1H Observe — RTB11

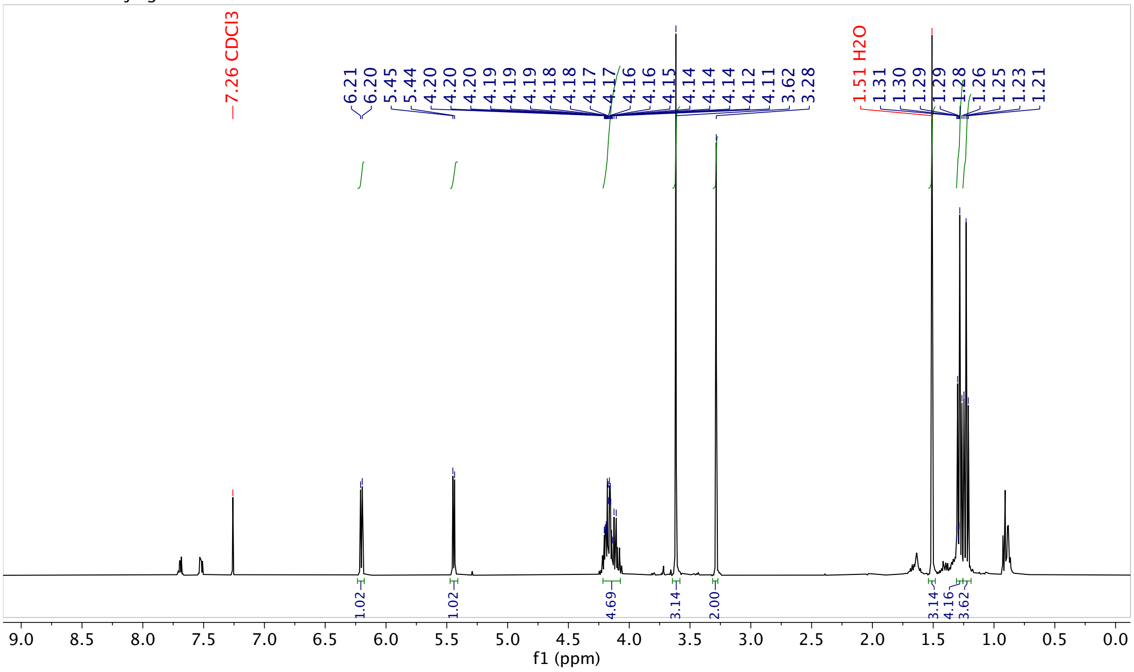


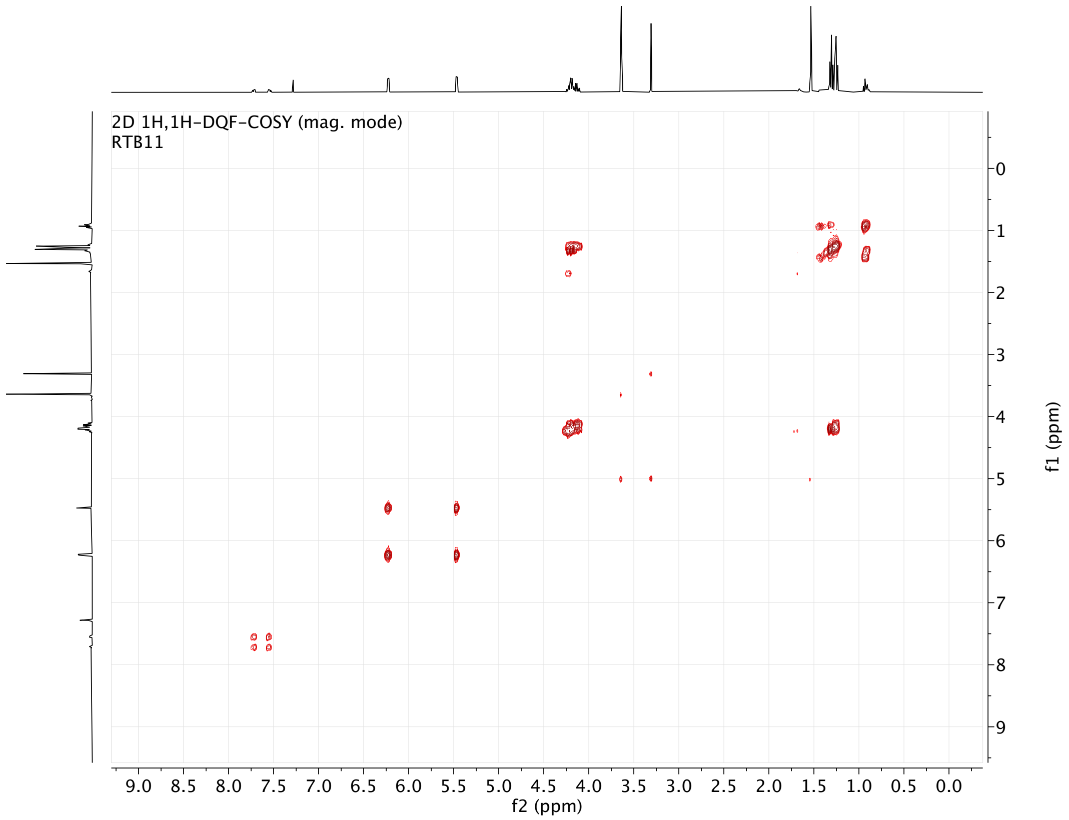

**(3b)**

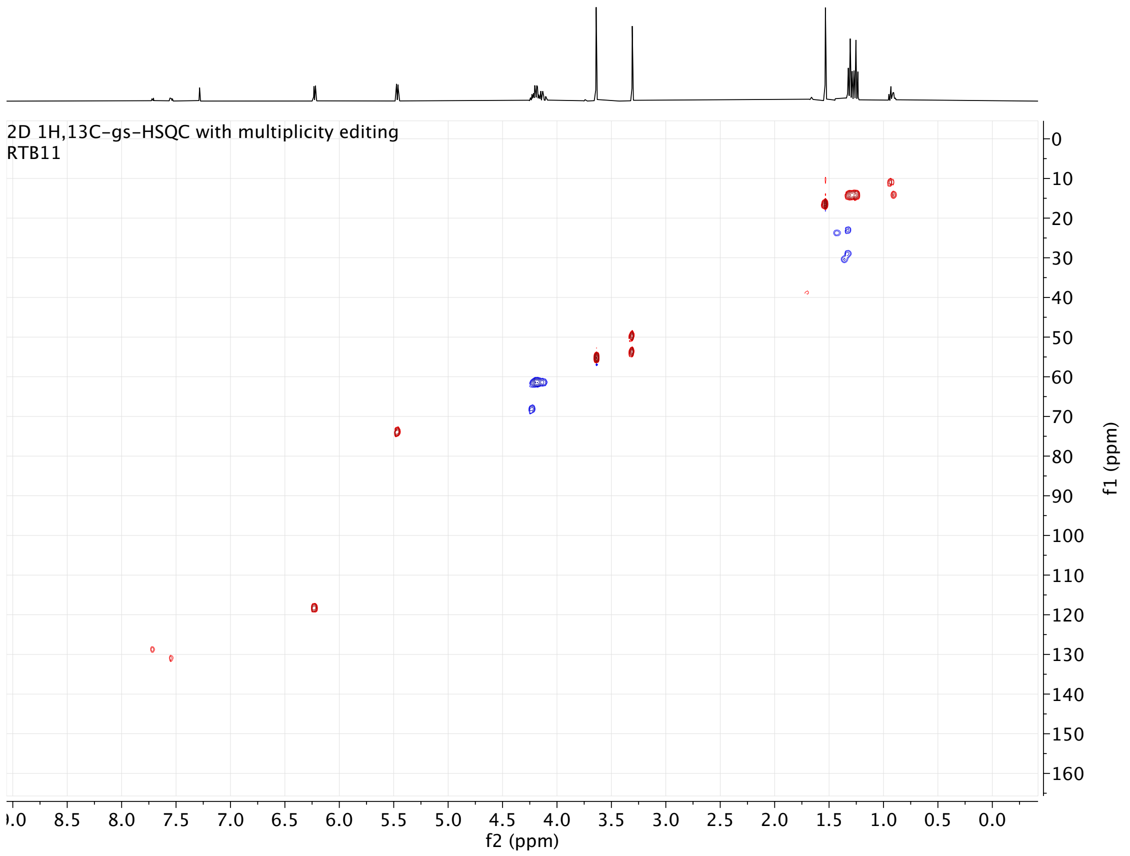
2D 1H,13C-gs-HSQC with multiplicity editing
RTB11
f2 (ppm)
f1 (ppm)

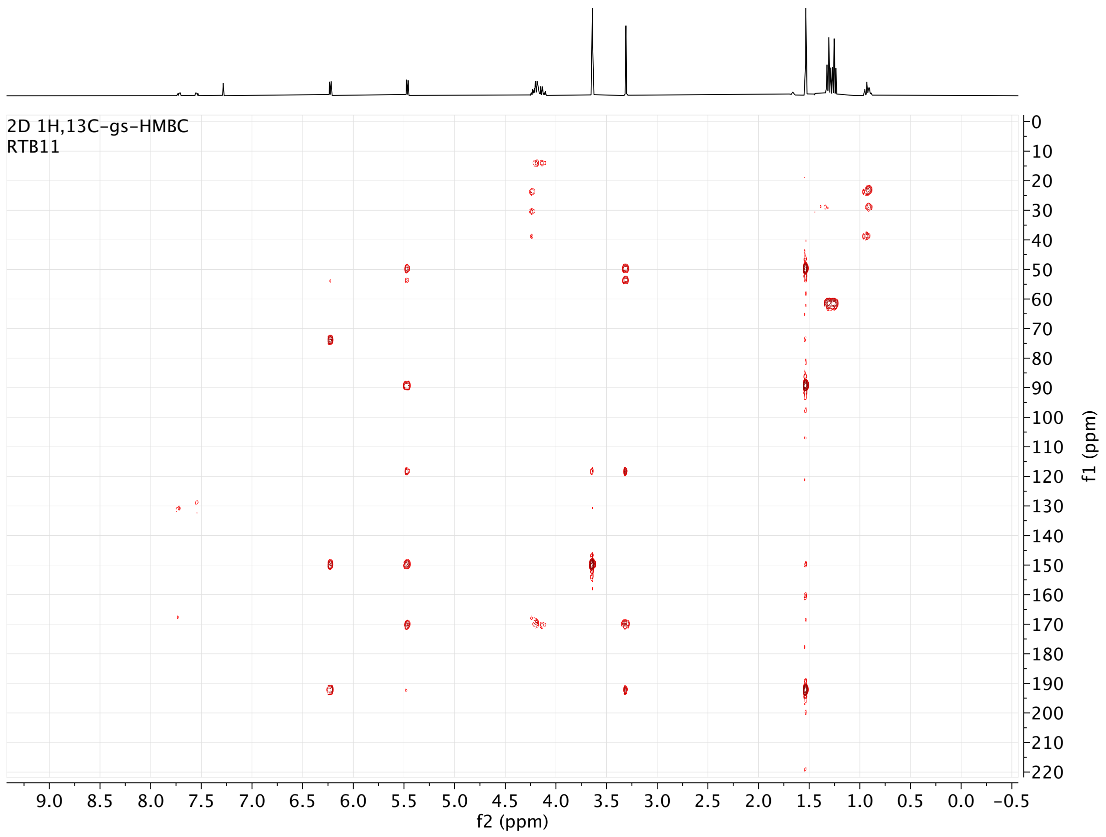
2D 1H,13C-gs-HMBC
RTB11
f2 (ppm)
f1 (ppm)

**(3b)**

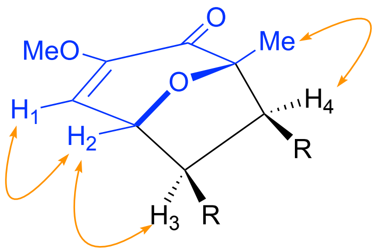
MeO
O
O
Me
H1
H2
H3
H4
R
R

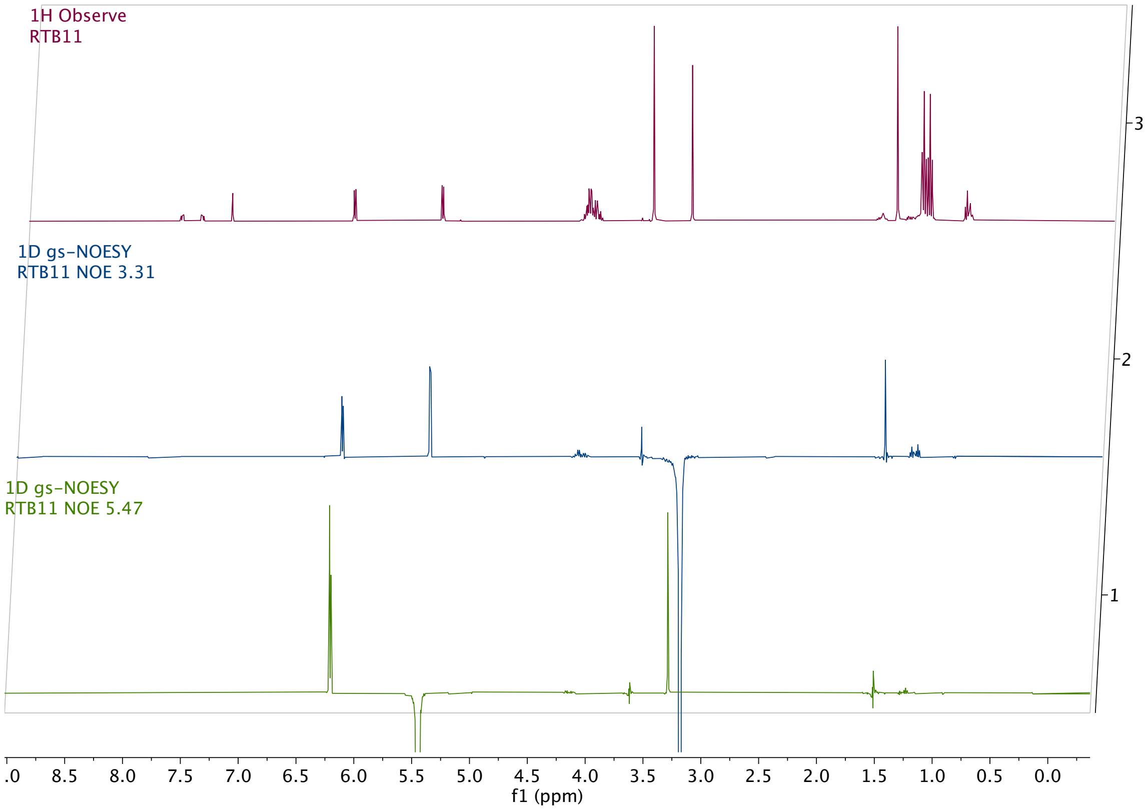
1H Observe
RTB11
1D gs-NOESY
RTB11 NOE 3.31
1D gs-NOESY
RTB11 NOE 5.47
f1 (ppm)

(**4**)

Dimethyl 3-methoxy-5-methyl-4-oxo-8-oxabicyclo[3.2.1]octa-2,6-diene-6,7-dicarboxylate (**4**)

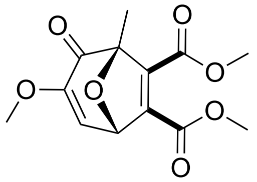

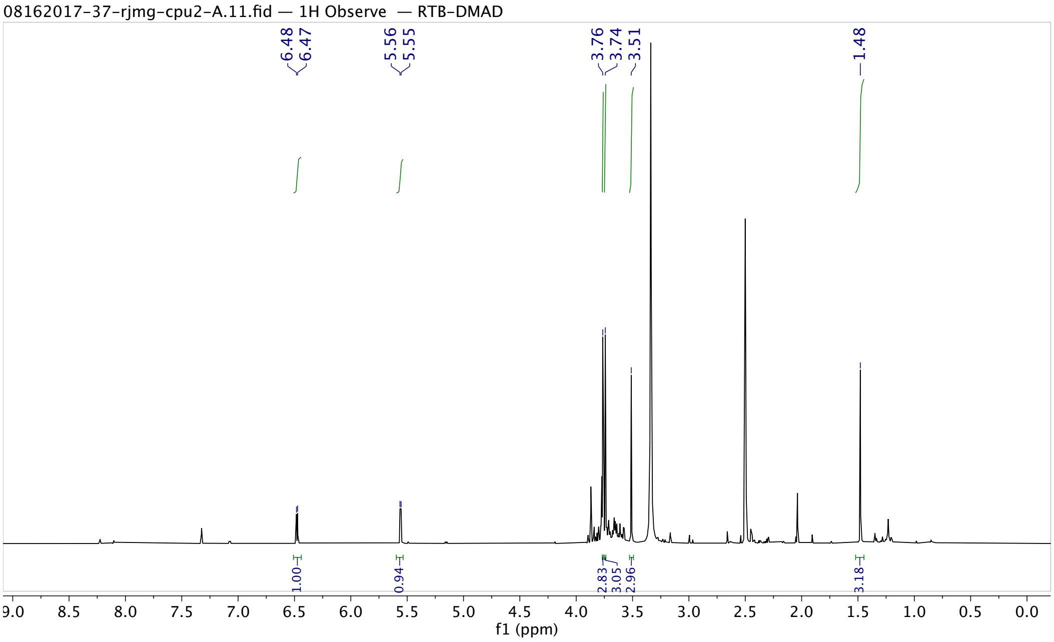


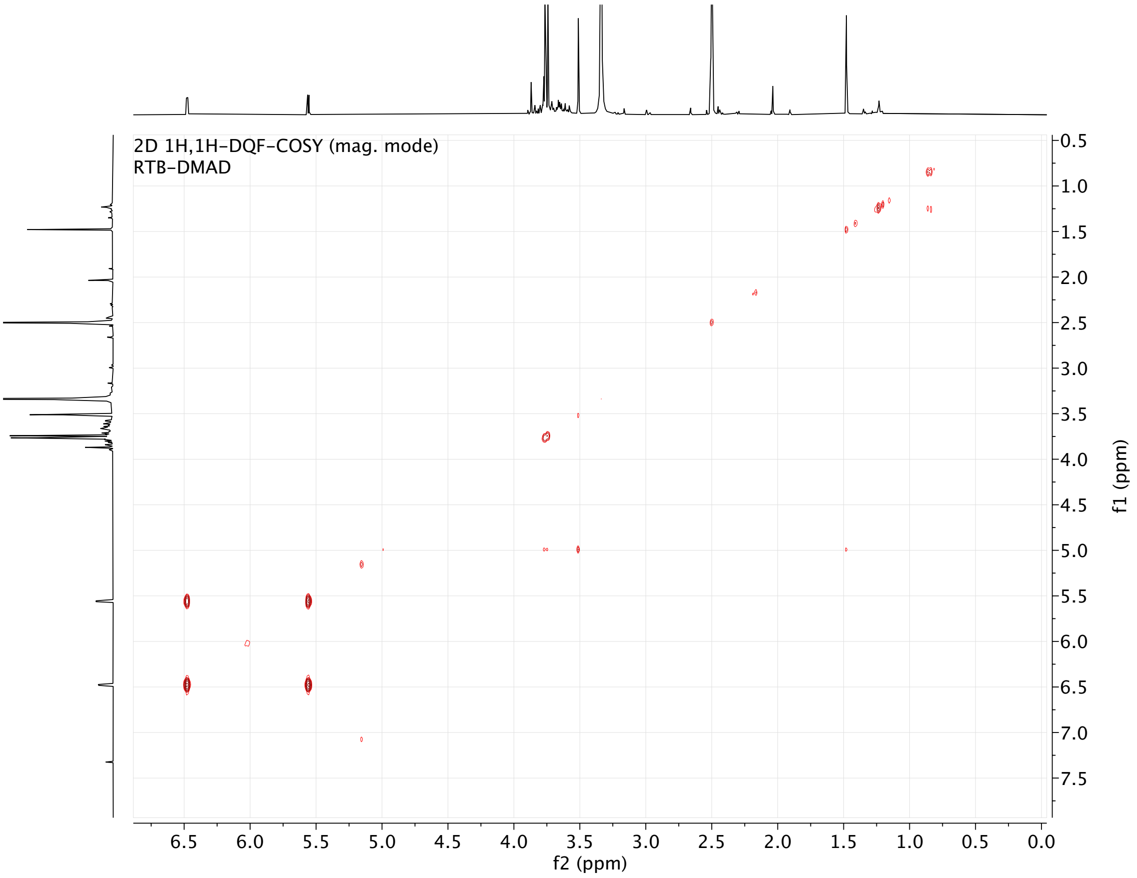

**(4)**

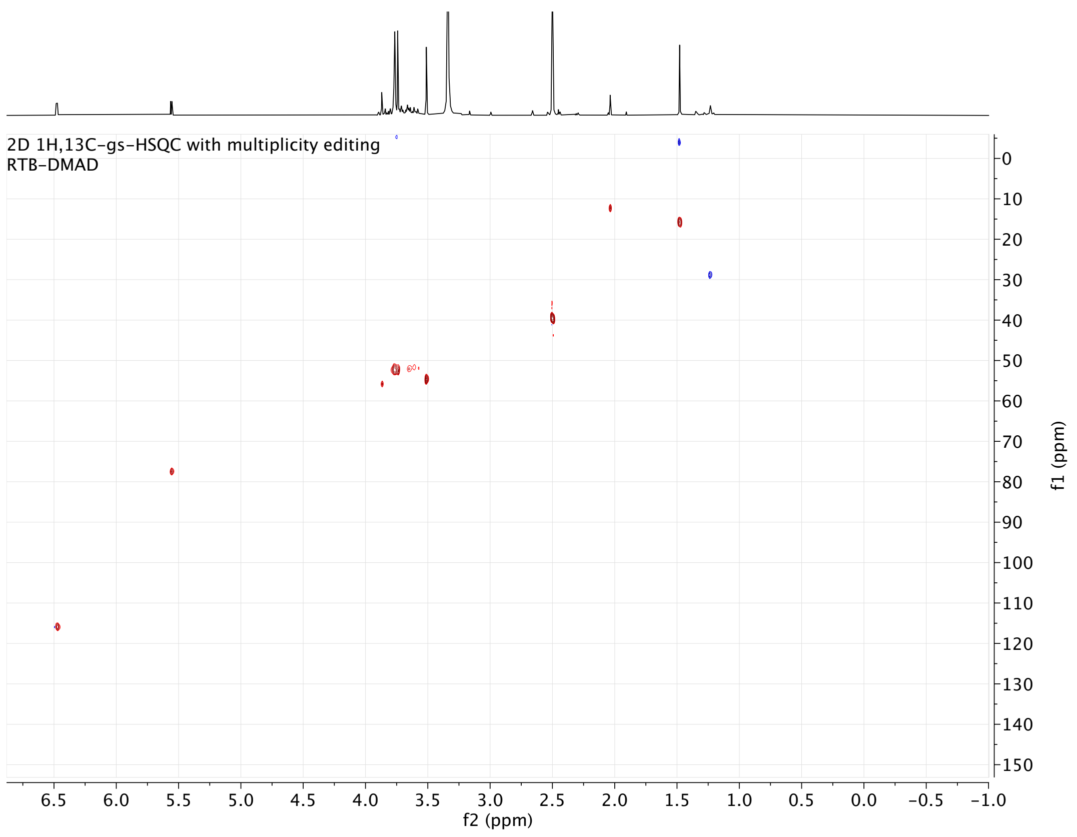

2D 1H,13C-gs-HSQC with multiplicity editing
RTB-DMAD
f1 (ppm)
f2 (ppm)


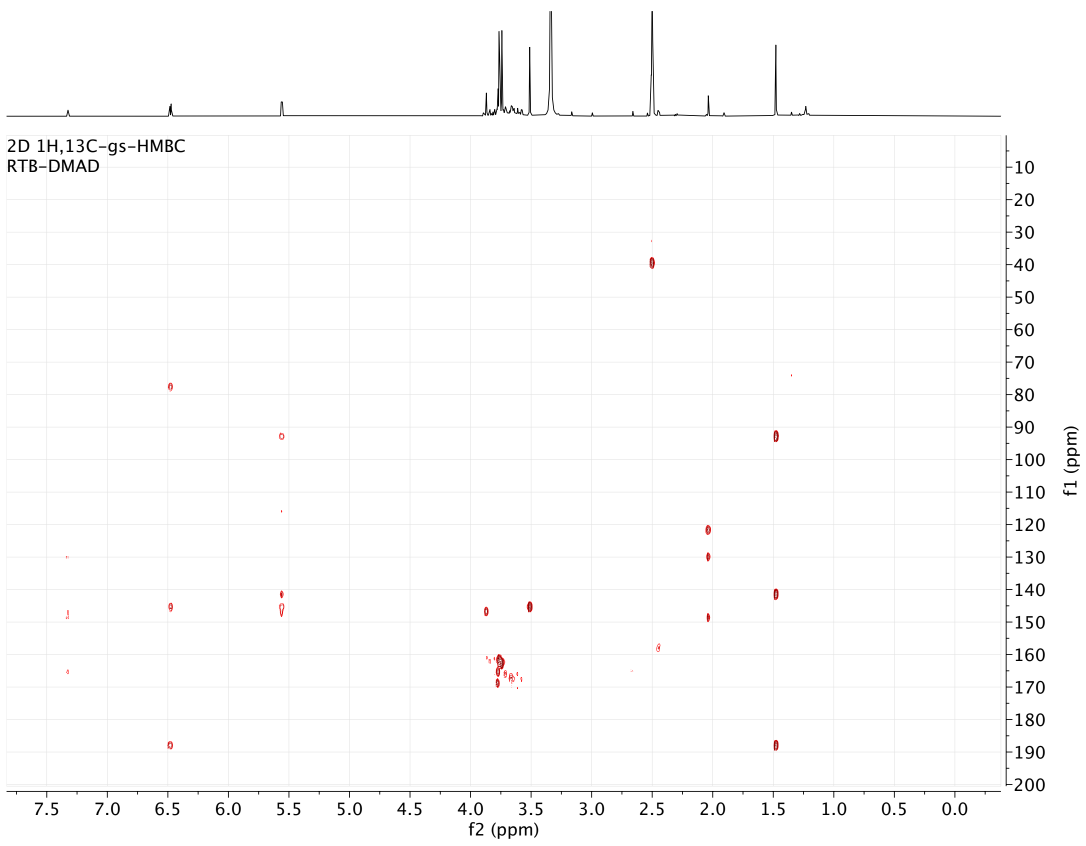

2D 1H,13C-gs-HMBC
RTB-DMAD
f1 (ppm)
f2 (ppm)

**(4)**

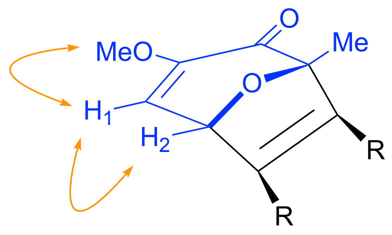

MeO
O
Me
O
H1
H2
R
R


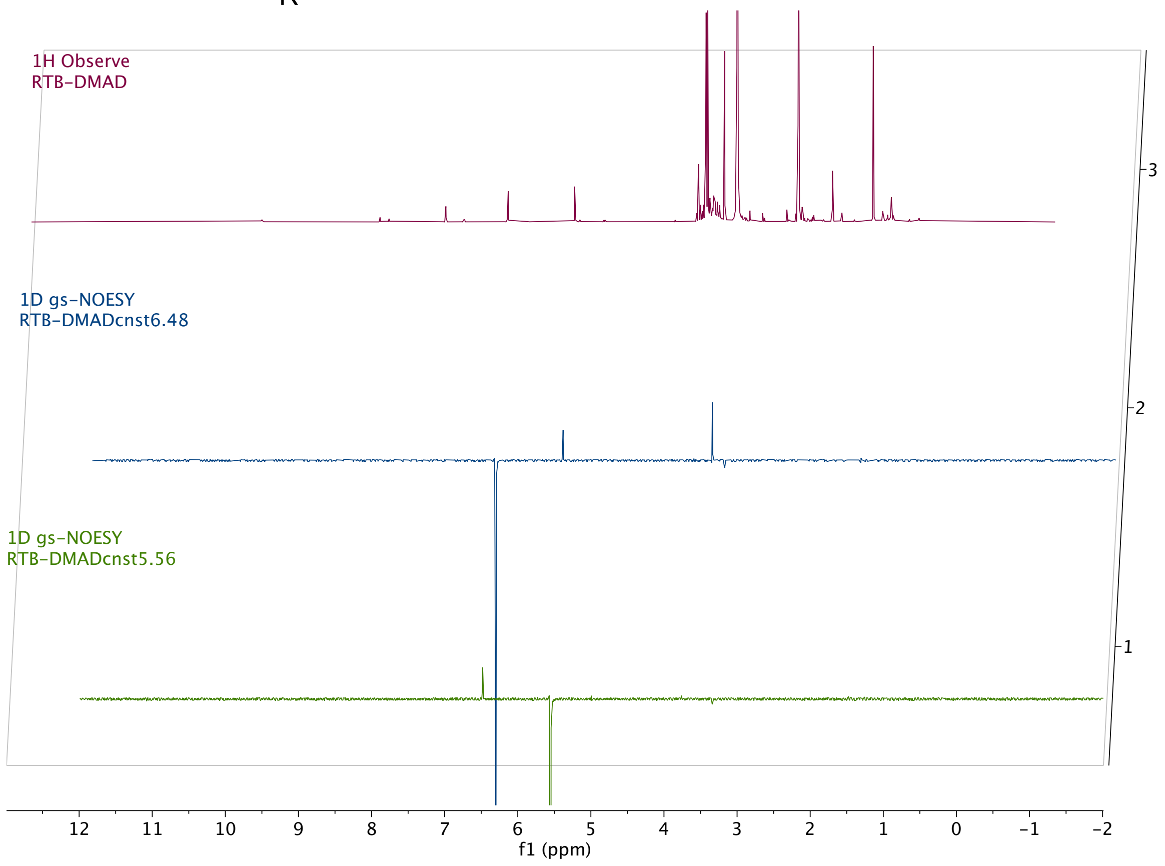

1H Observe
RTB-DMAD
1D gs-NOESY
RTB-DMADcnst6.48
1D gs-NOESY
RTB-DMADcnst5.56
f1 (ppm)

**(5)**

(3-Methoxy-1-methyl-2-oxo-7-phenyl-8-oxabicyclo[3.2.1]oct-3-en-6-yl)methyl acetate (**5**)

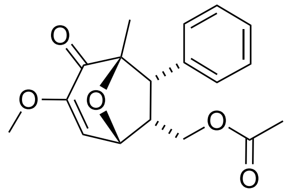

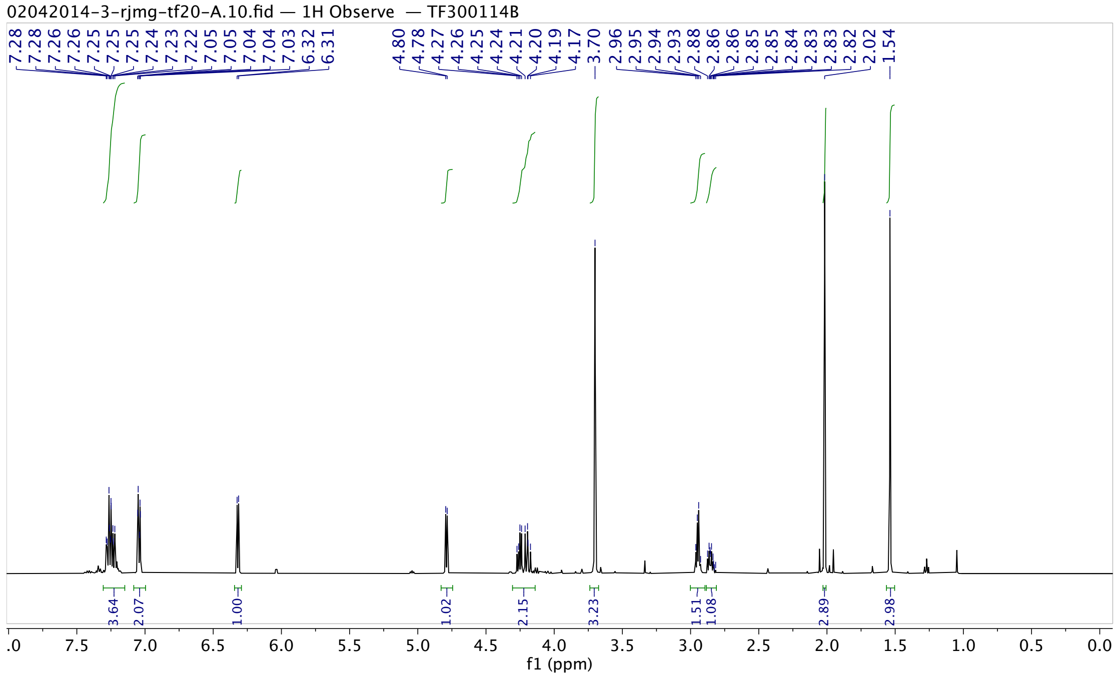


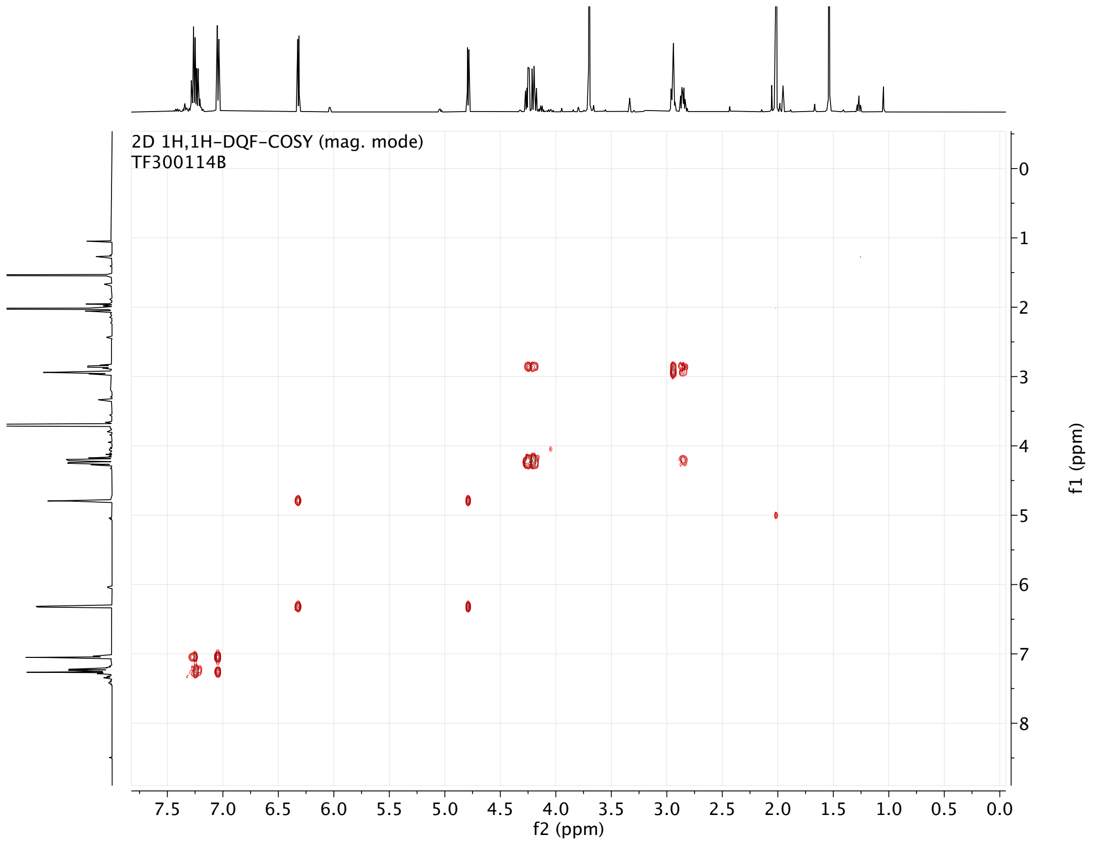

**(5)**

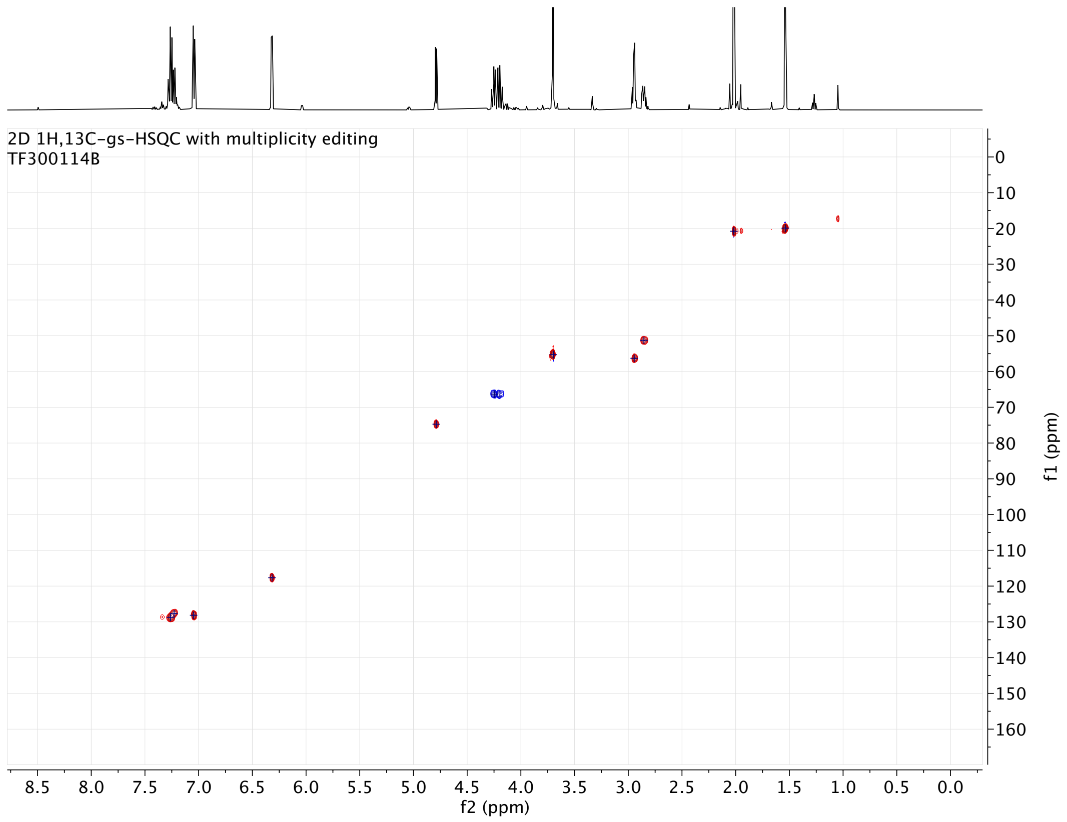

2D 1H,13C-gs-HSQC with multiplicity editing
TF300114B
f1 (ppm)
f2 (ppm)


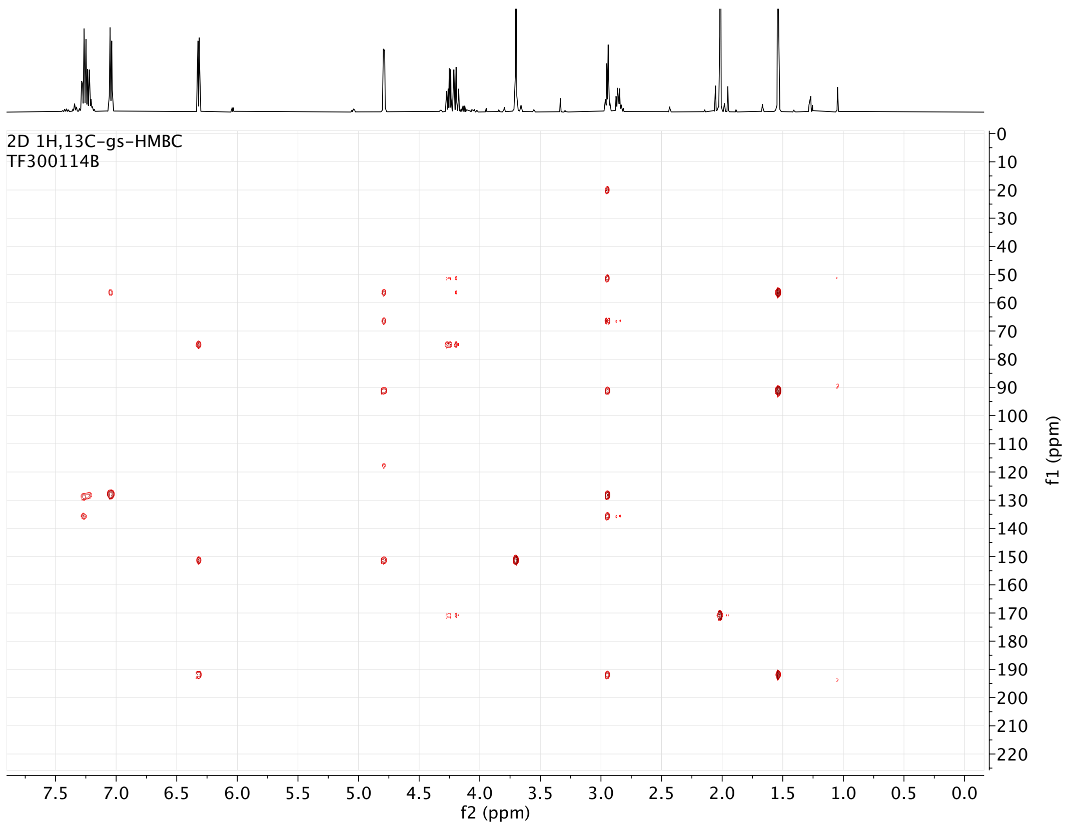

2D 1H,13C-gs-HMBC
TF300114B
f1 (ppm)
f2 (ppm)

**(5)**

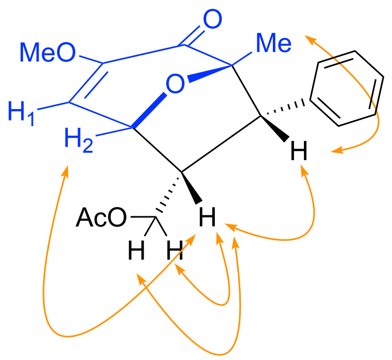
MeO
O
Me
H1
O
H2
H
AcO
H
H
H

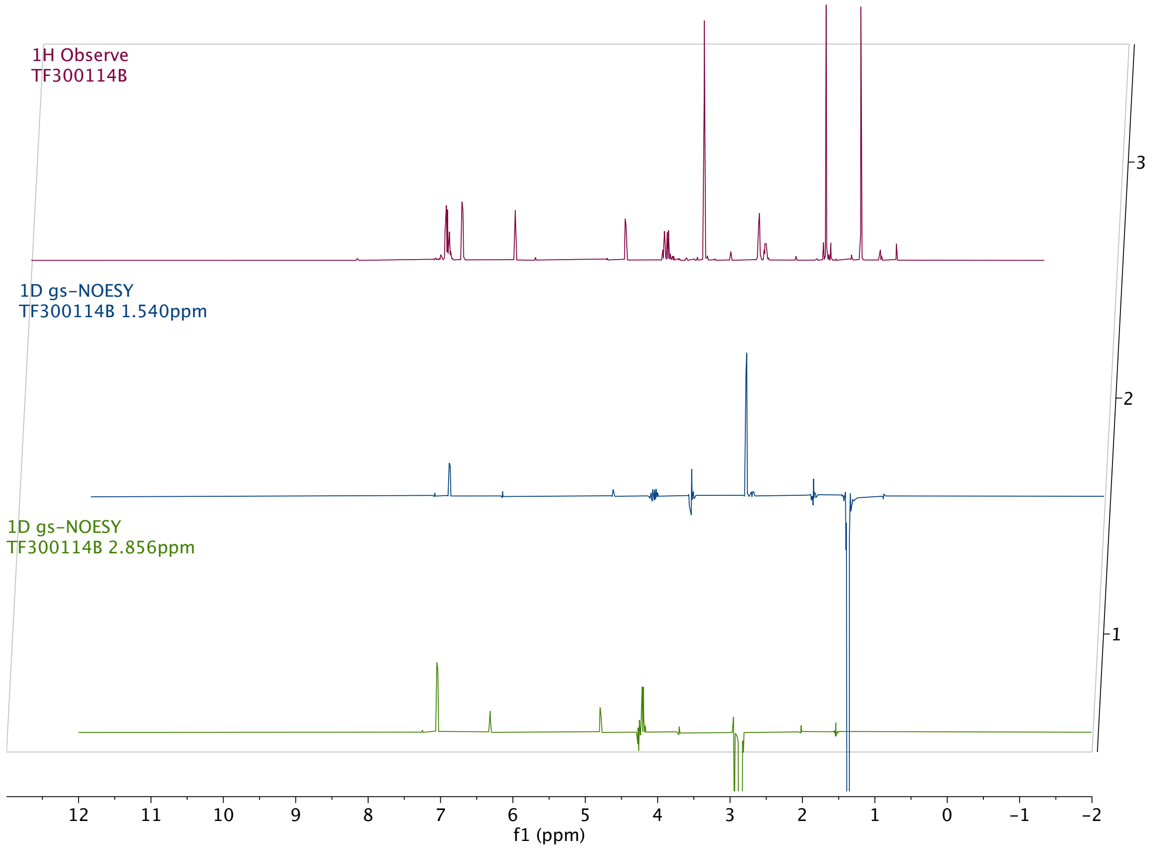
1H Observe
TF300114B
1D gs-NOESY
TF300114B 1.540ppm
1D gs-NOESY
TF300114B 2.856ppm
3
2
1
12
11
10
9
8
7
6
5
4
3
2
1
0
−1
−2
f1 (ppm)

(**6**)

(1*R*,2*S*,6*S*,7*R*)-6,9-Dimethoxy-1,2-dimethyl-3,11-dioxatricyclo[5.3.1.12,6]dodeca-4,8-diene-10,12-dione (**6**)

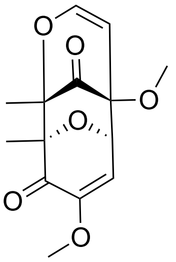

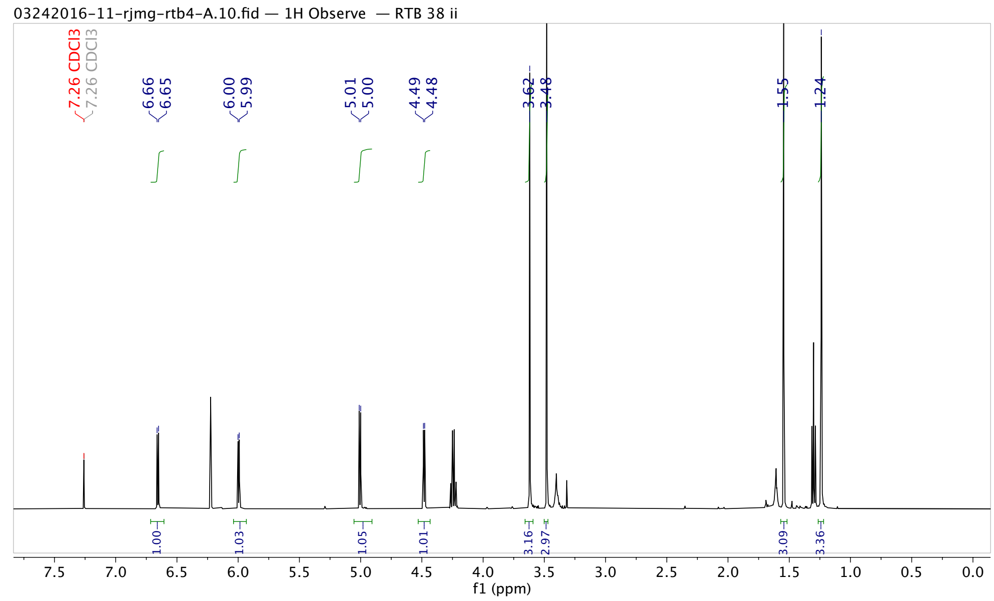


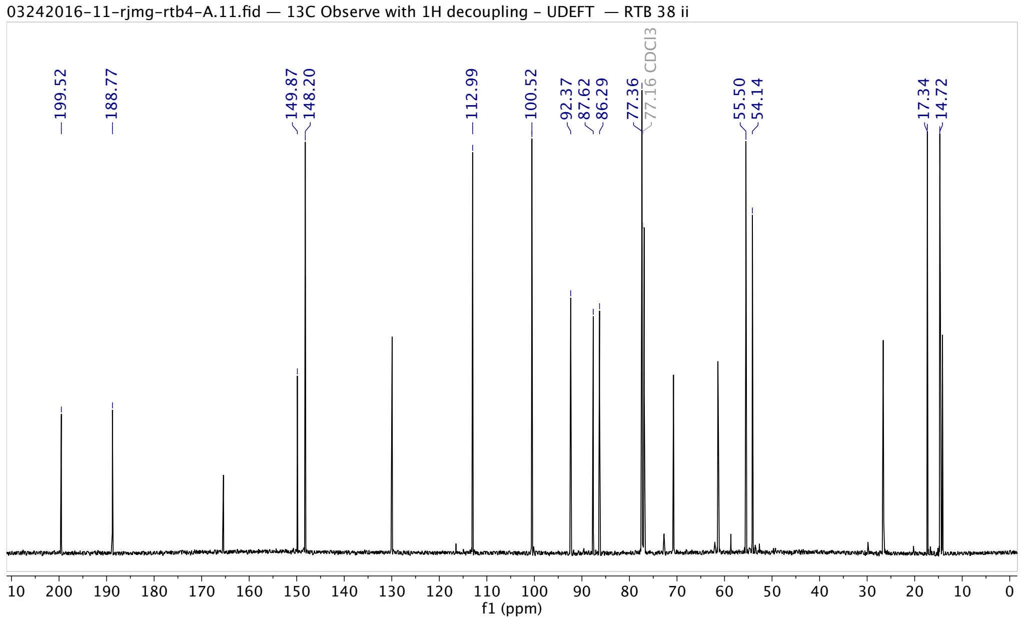

**(6)**

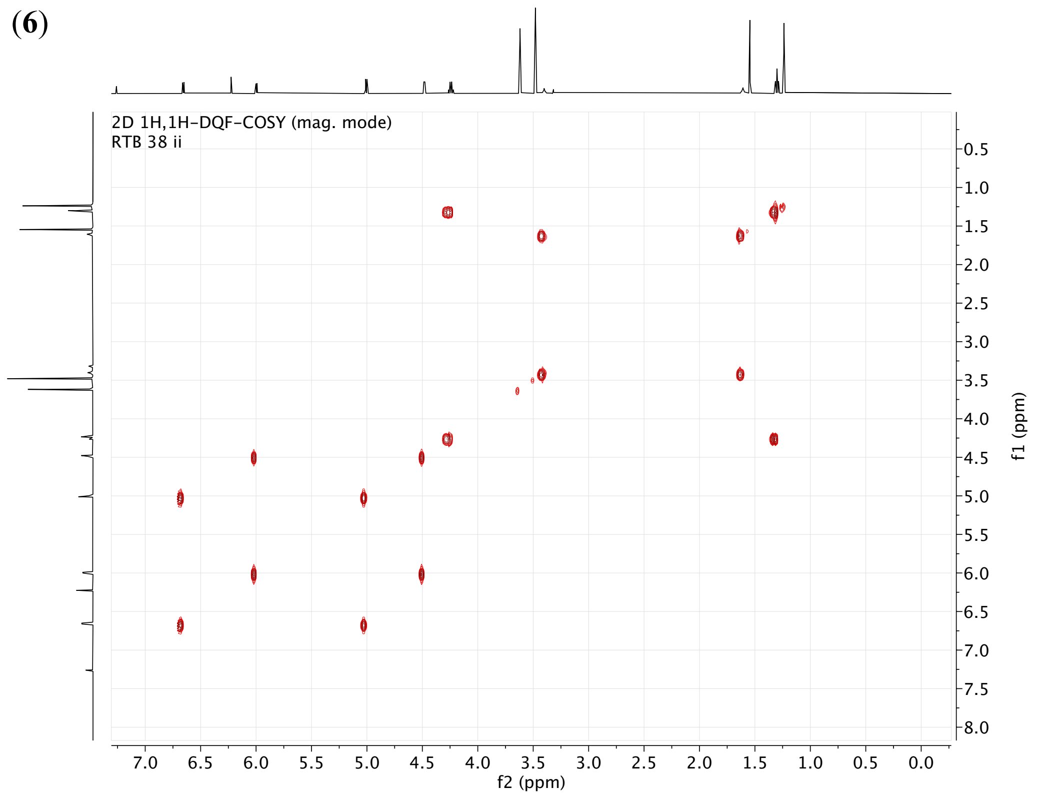
2D 1H,1H-DQF-COSY (mag. mode)
RTB 38 ii
f2 (ppm)
f1 (ppm)

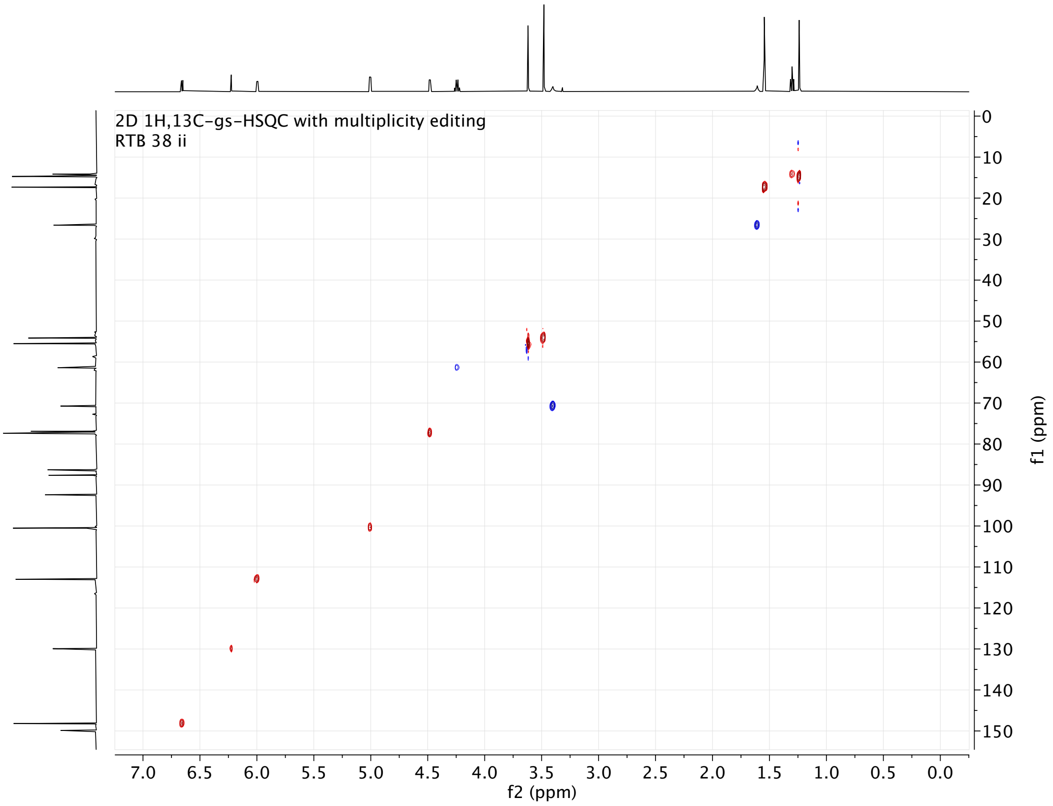
2D 1H,13C-gs-HSQC with multiplicity editing
RTB 38 ii
f2 (ppm)
f1 (ppm)

**(6 & 7)**

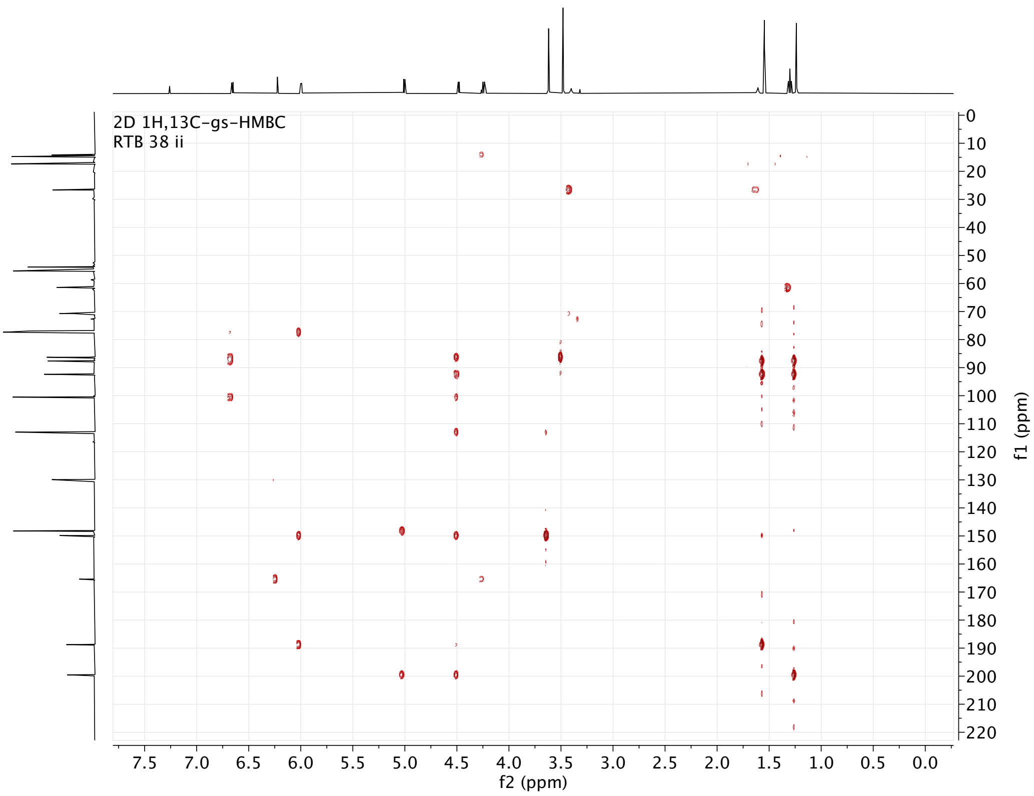


3-Methoxy-1-methyl-6-phenyl-8-oxabicyclo[3.2.1]oct-3-en-2-one (**7**)

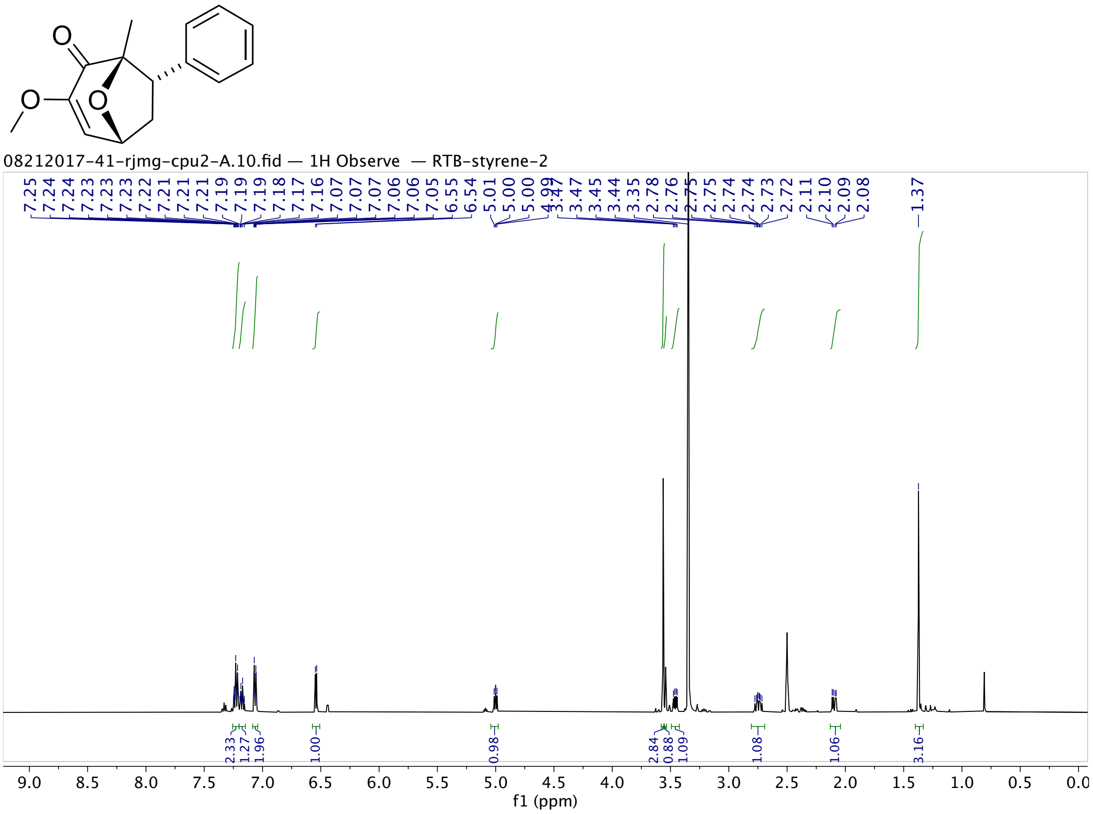

(7)

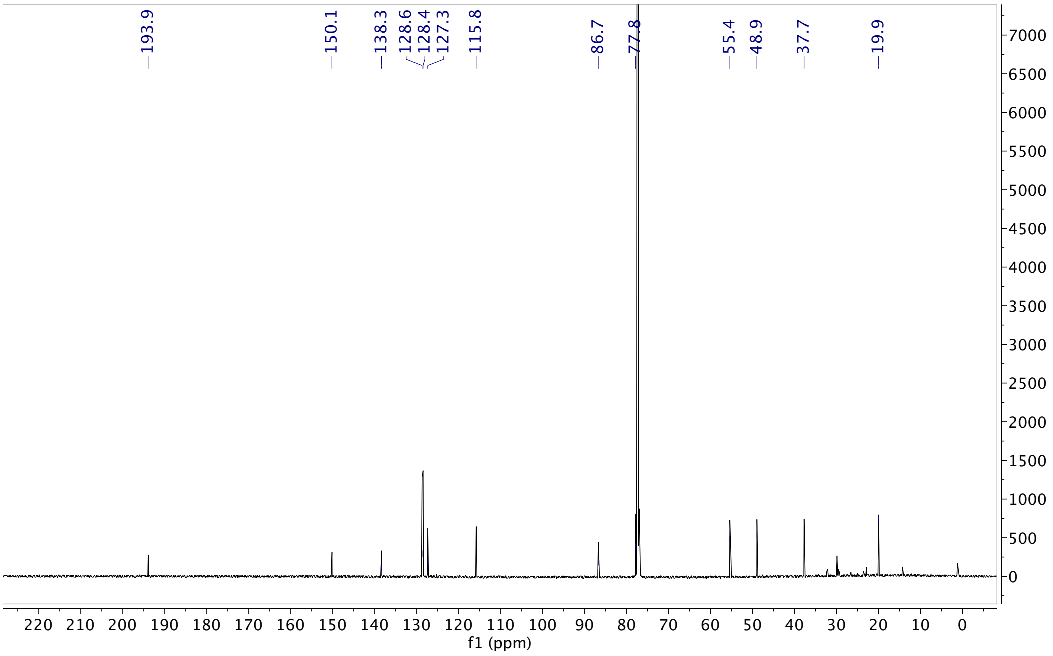
193.9
150.1
138.3
128.6
128.4
127.3
115.8
86.7
77.8
55.4
48.9
37.7
19.9
f1 (ppm)

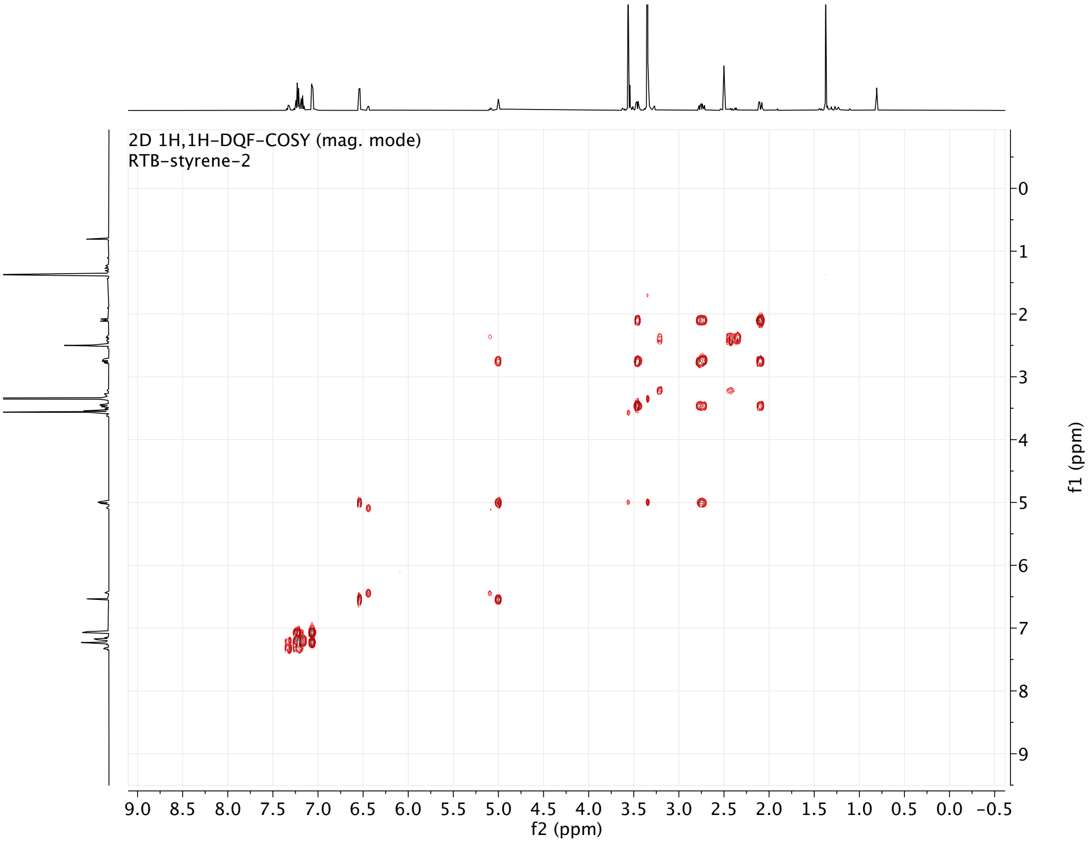
2D 1H,1H-DQF-COSY (mag. mode)
RTB-styrene-2
f2 (ppm)
f1 (ppm)

(7)

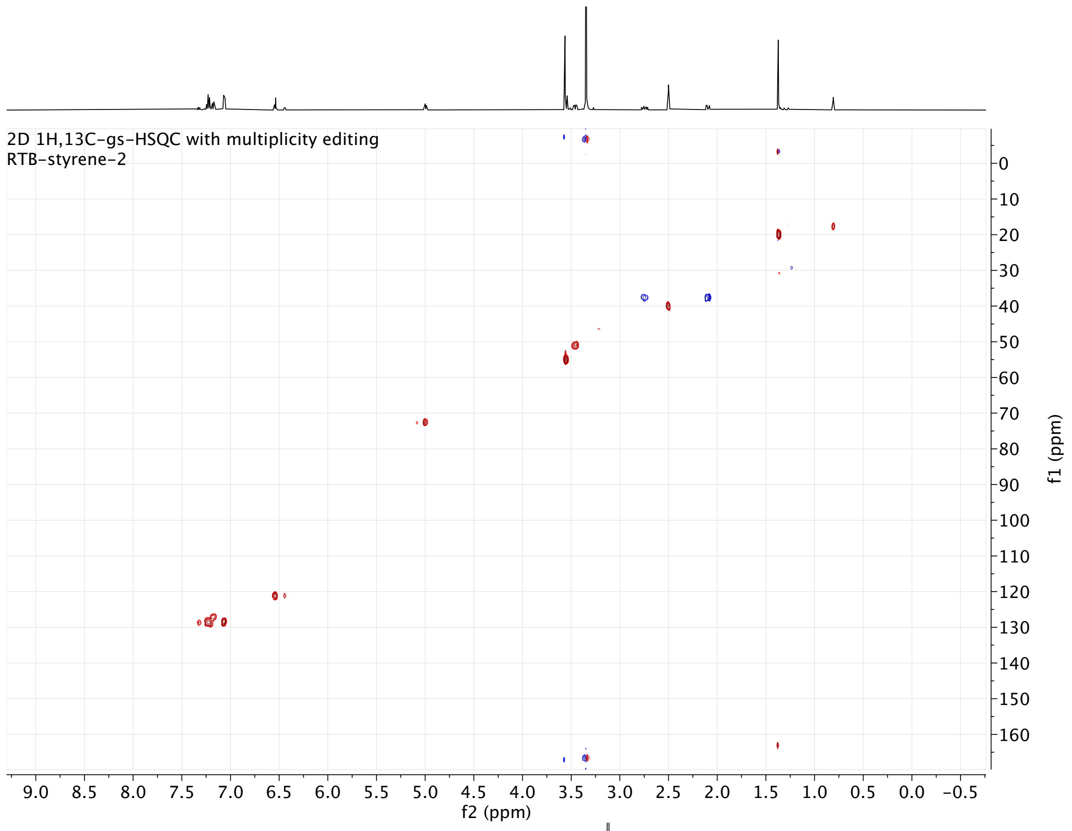
2D 1H,13C-gs-HSQC with multiplicity editing
RTB-styrene-2
f1 (ppm)
f2 (ppm)

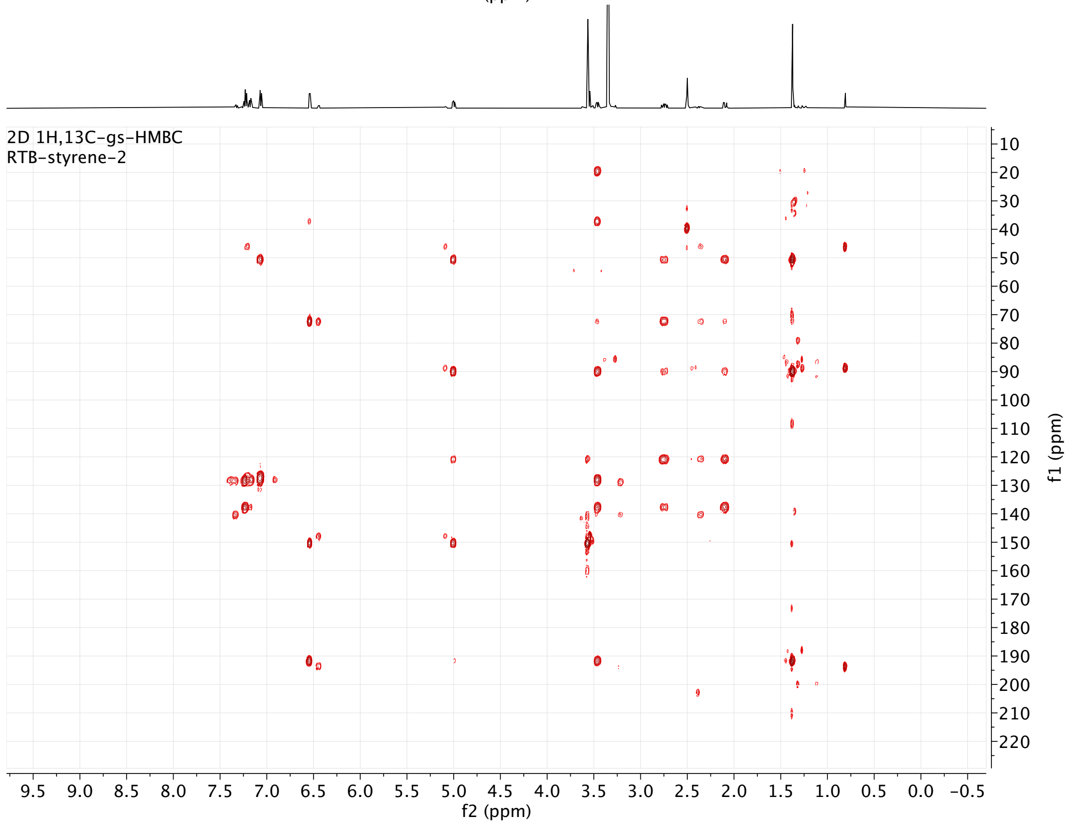
2D 1H,13C-gs-HMBC
RTB-styrene-2
f1 (ppm)
f2 (ppm)

(7)

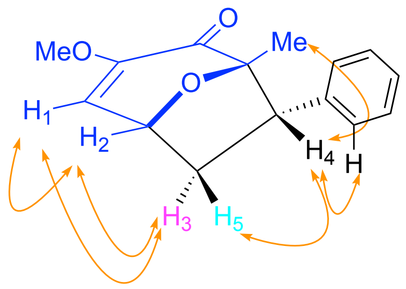


H1 6.54 ppm
H2 5.00 - NOE 6.54, 2.75
H3 2.10 -NOE Ph, 6.54, 5.00, 2.75
H4 3.46 - NOE Ph, Me, 2.75
H5 2.75 - NOE 5.00, 3.46. 2.10

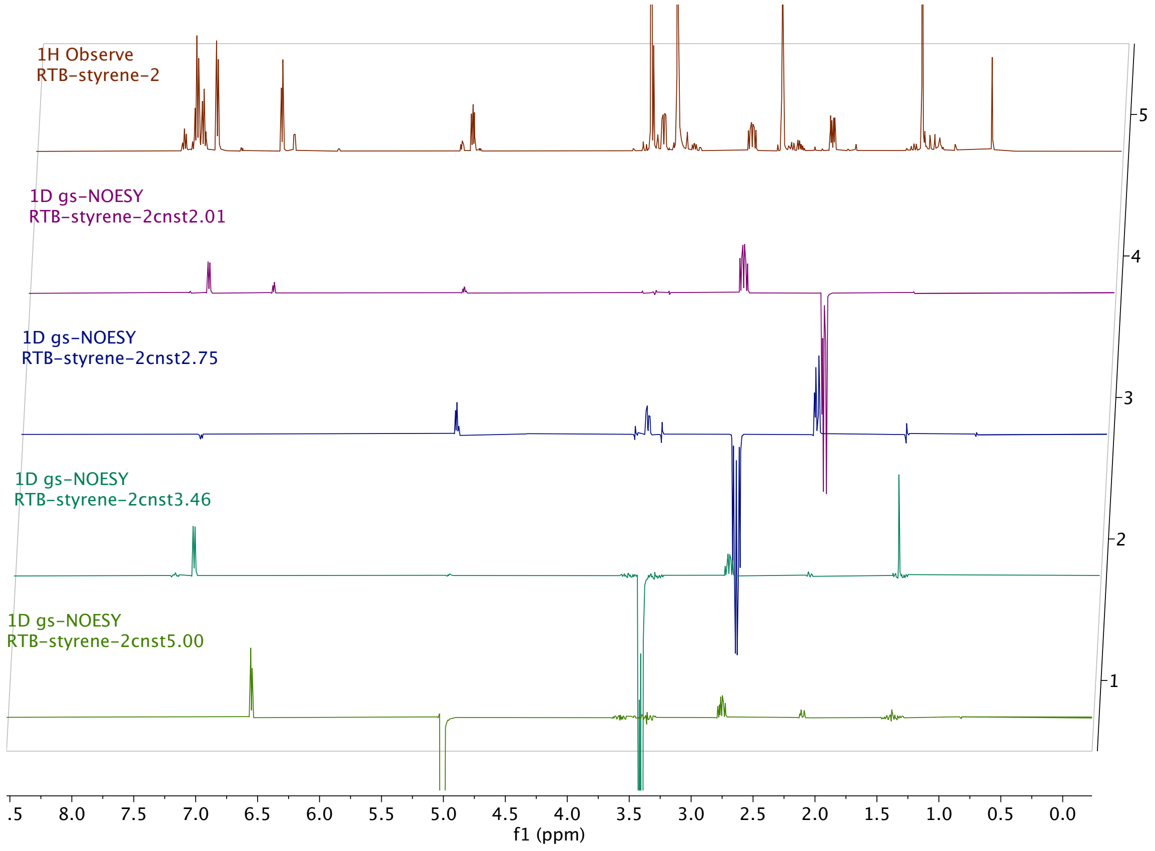

(**8**)

3-Methoxy-1-methyl-2-oxo-7-phenyl-8-oxabicyclo[3.2.1]oct-3-ene-6-carboxylic acid (**8**)

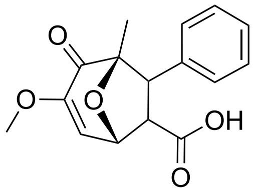


05032016-10-rjmg-rtb4-A.10.fid — 1H Observe — RTB 85 ii

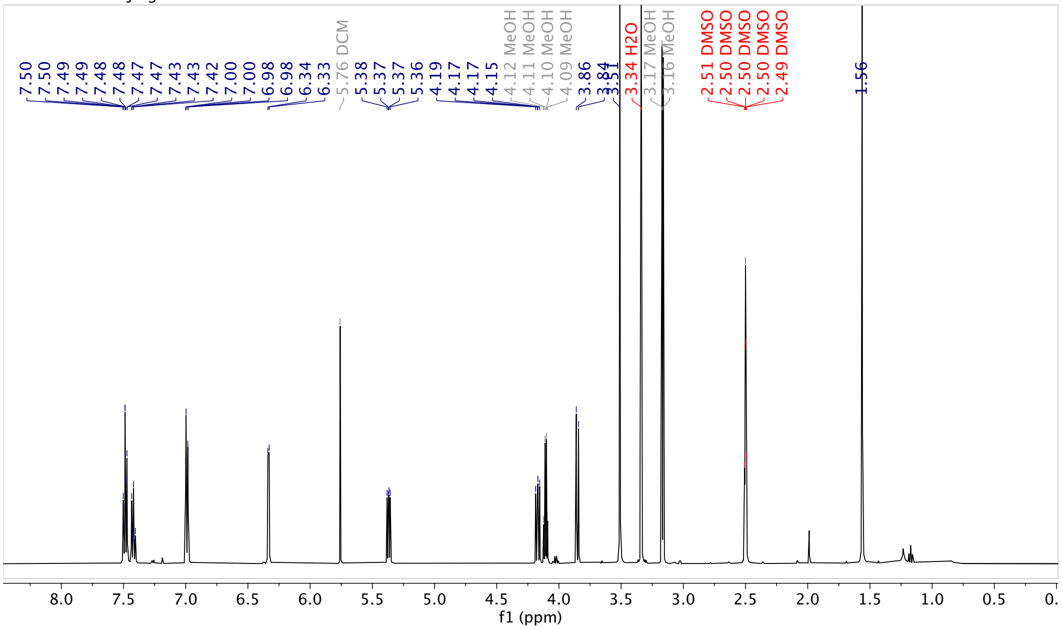


05032016-10-rjmg-rtb4-A.11.fid — 13C Observe with 1H decoupling - UDEFT — RTB 85 ii

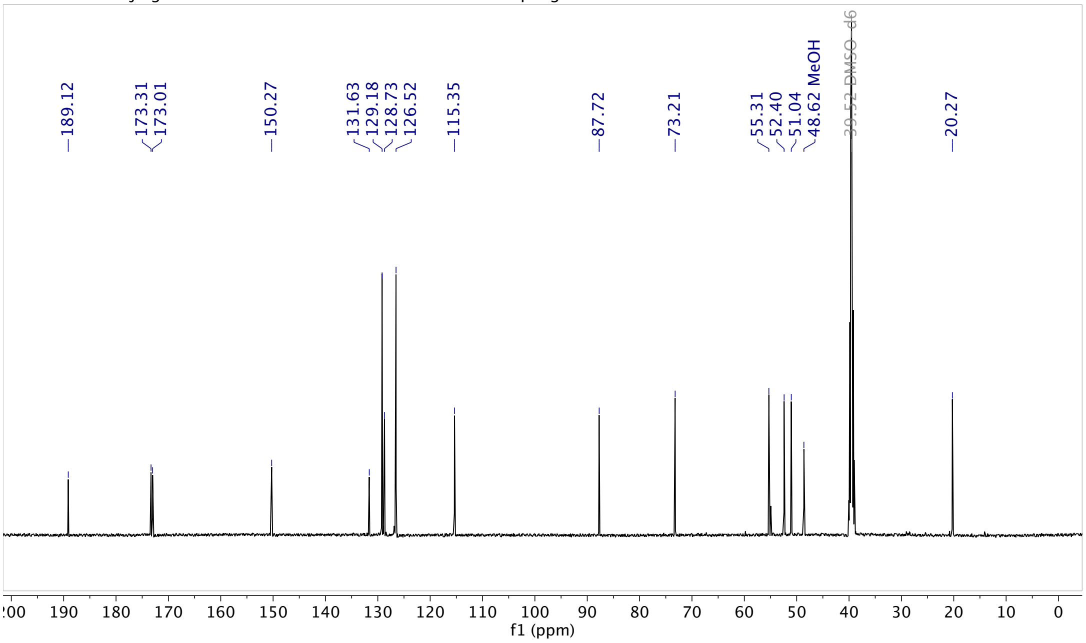

**(8)**

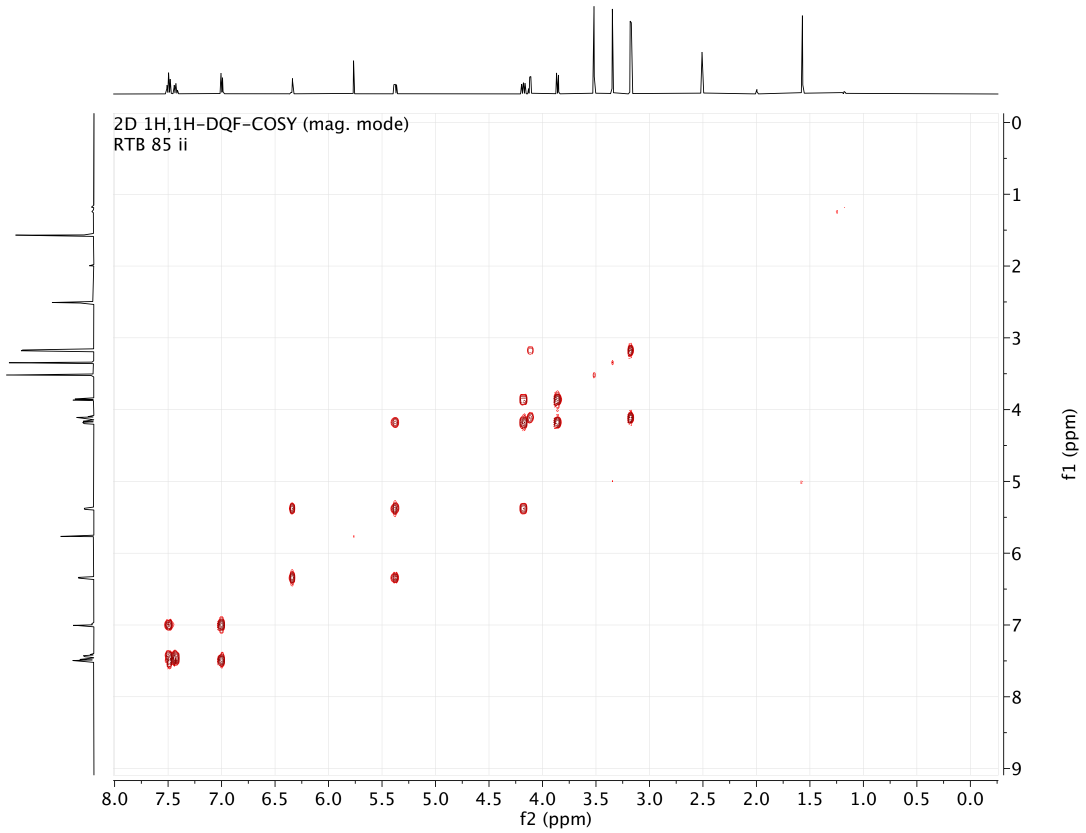
2D 1H,1H-DQF-COSY (mag. mode)
RTB 85 ii
f2 (ppm)
f1 (ppm)

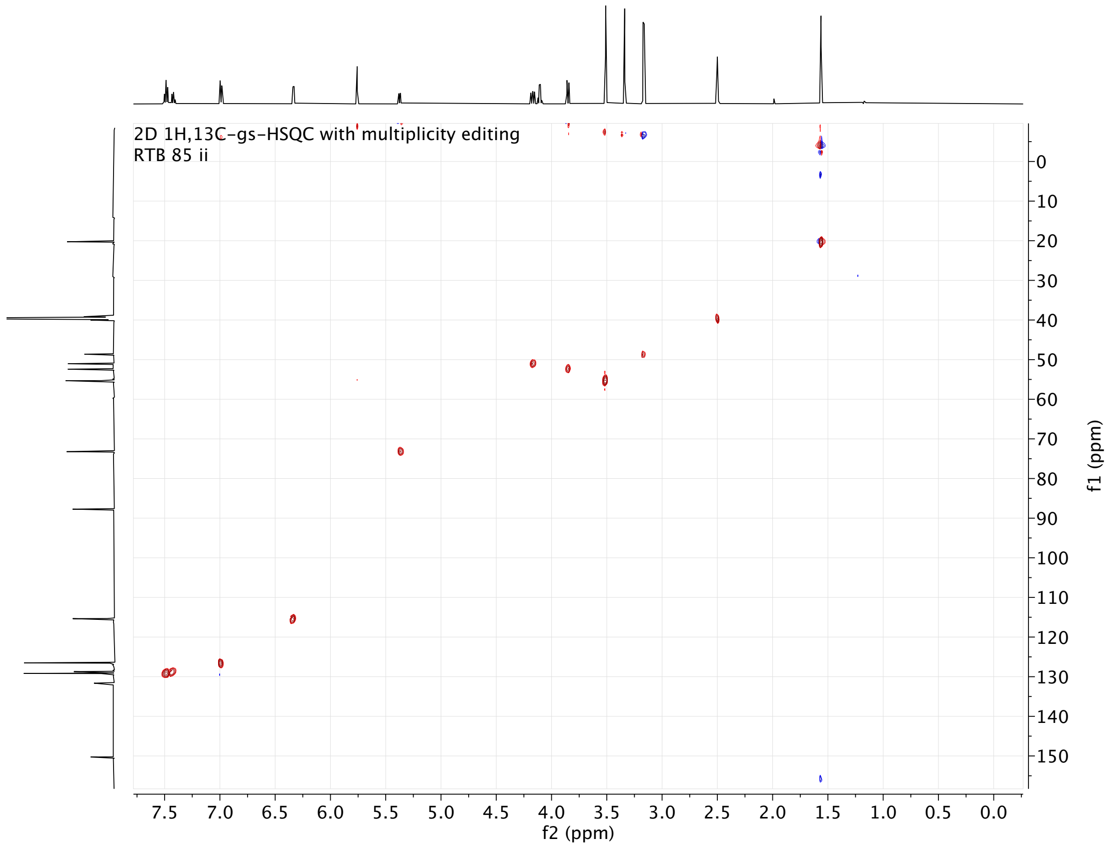
2D 1H,13C-gs-HSQC with multiplicity editing
RTB 85 ii
f2 (ppm)
f1 (ppm)

**(8 & 9a)**

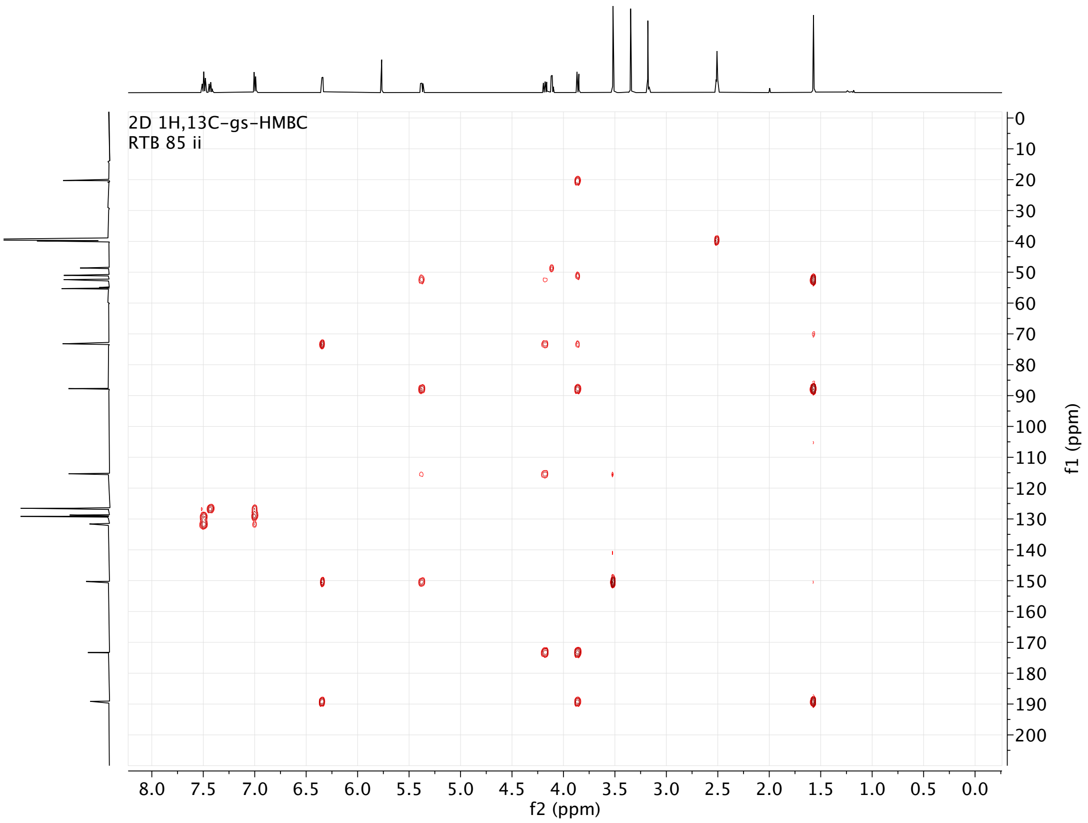


8-Methoxy-6-methyl-5a,6,10,10a-tetrahydro-5*H*-6,10-epoxycyclohepta[*b*]naphthalene-5,7,11-trione (**9a**)

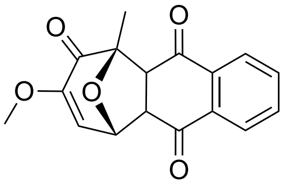

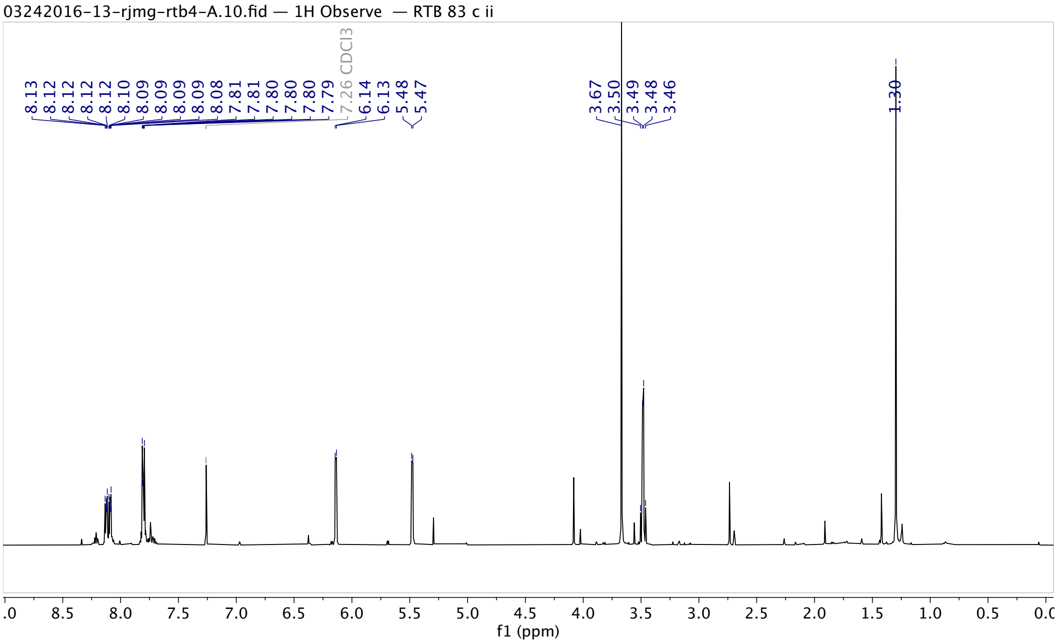

**(9a)**

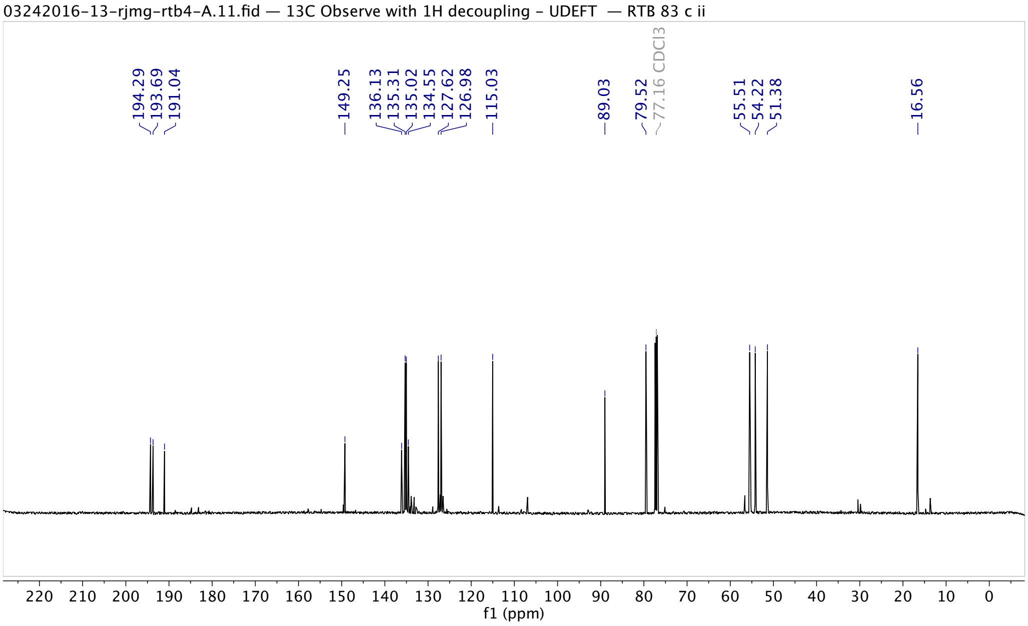

03242016-13-rjmg-rtb4-A.11.fid — 13C Observe with 1H decoupling - UDEFT — RTB 83 c ii
194.29
193.69
191.04
149.25
136.13
135.31
135.02
134.55
127.62
126.98
115.03
89.03
79.52
77.16 CDCl3
55.51
54.22
51.38
16.56
220 210 200 190 180 170 160 150 140 130 120 110 100 90 80 70 60 50 40 30 20 10 0
f1 (ppm)


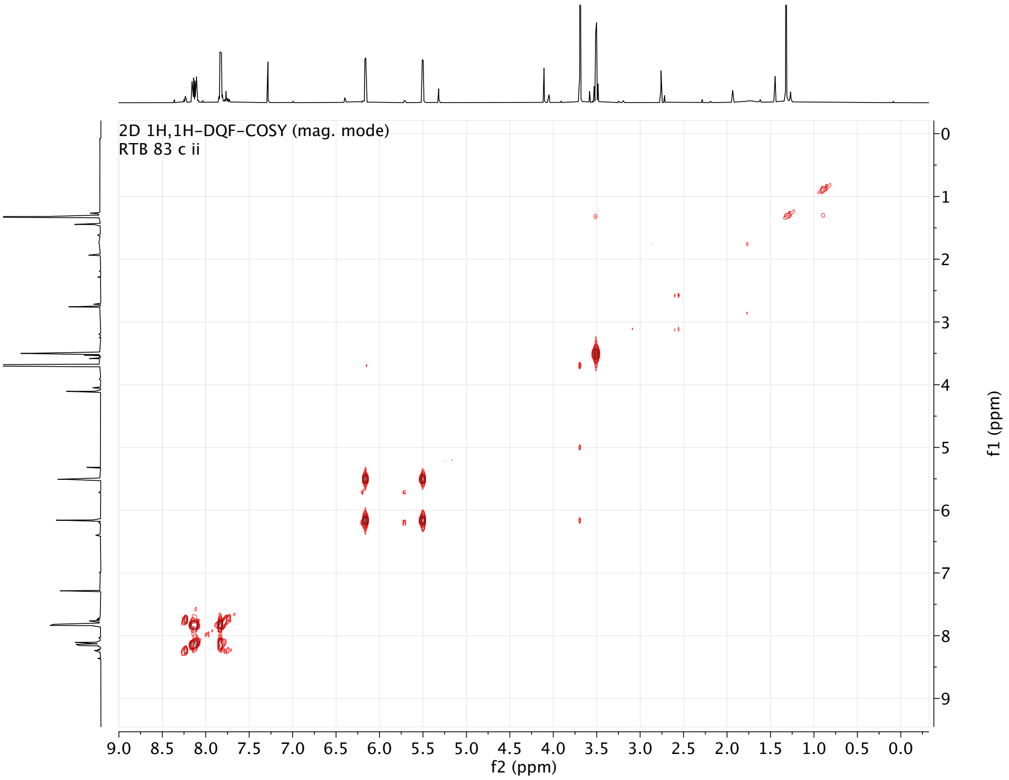

2D 1H,1H-DQF-COSY (mag. mode)
RTB 83 c ii
9.0 8.5 8.0 7.5 7.0 6.5 6.0 5.5 5.0 4.5 4.0 3.5 3.0 2.5 2.0 1.5 1.0 0.5 0.0
f2 (ppm)
0 1 2 3 4 5 6 7 8 9
f1 (ppm)

**(9a)**

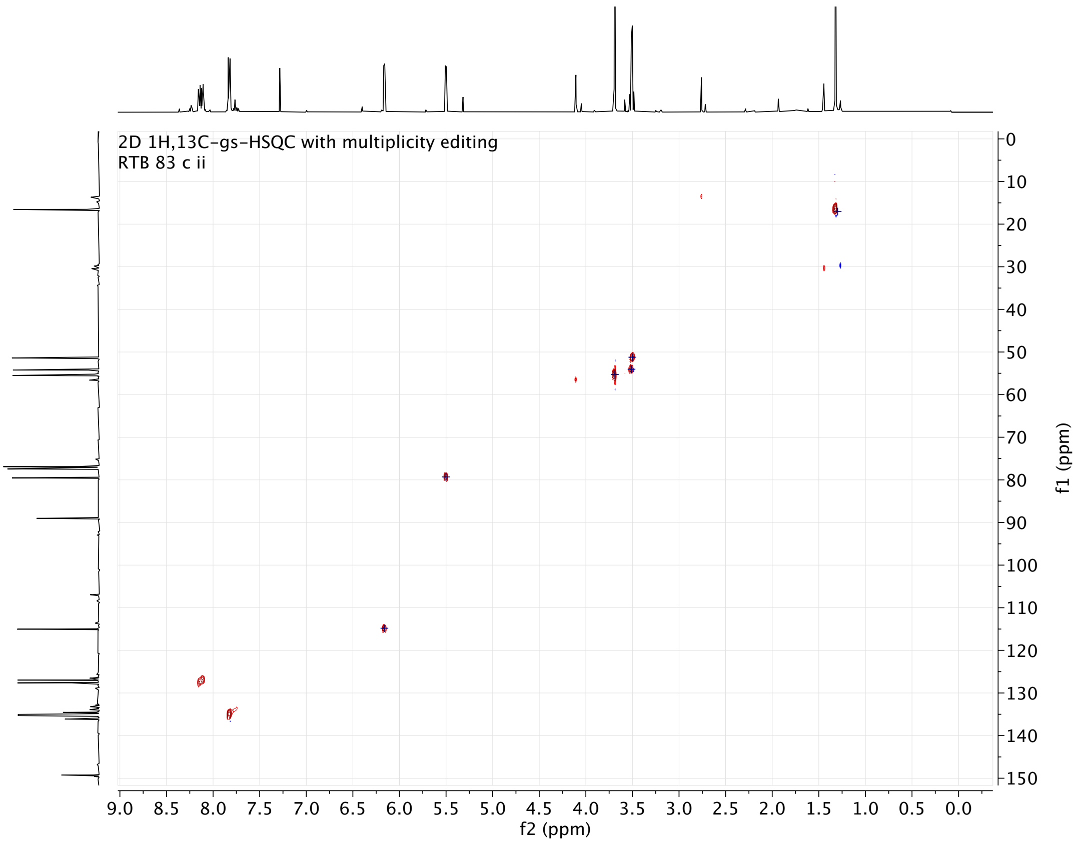

2D 1H,13C-gs-HSQC with multiplicity editing
RTB 83 c ii
f2 (ppm)
f1 (ppm)


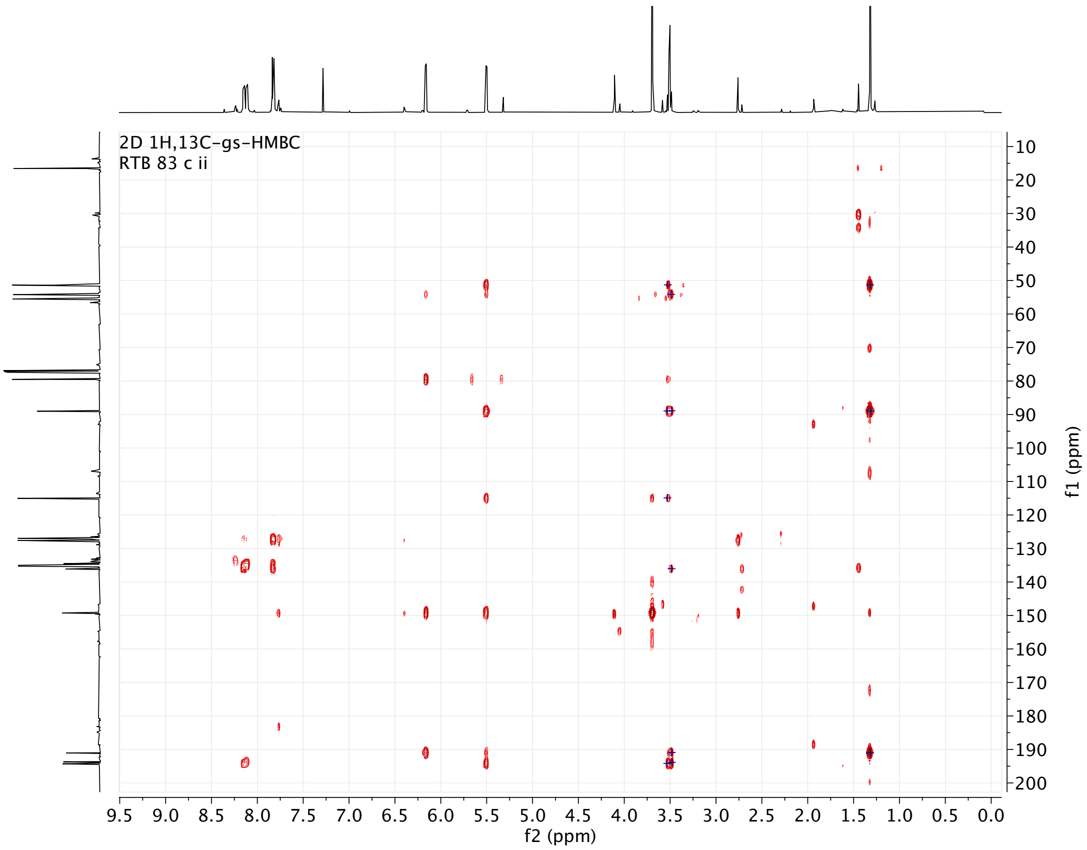

2D 1H,13C-gs-HMBC
RTB 83 c ii
f2 (ppm)
f1 (ppm)

**(9b)**

1-Acetyl-2-methoxyanthracene-9,10-dione (**9b**)

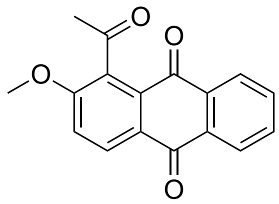

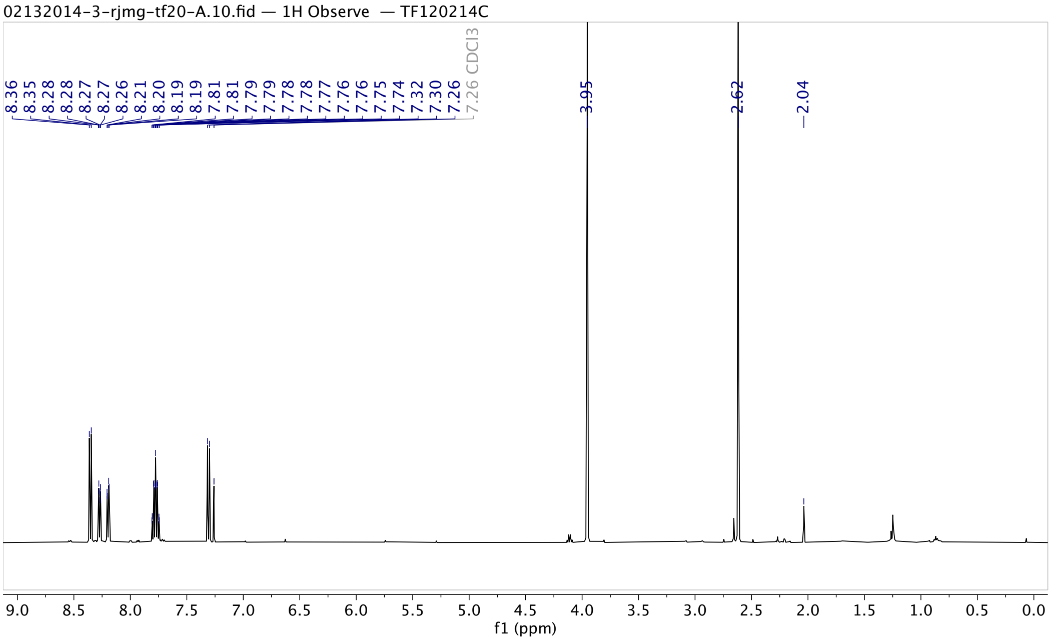


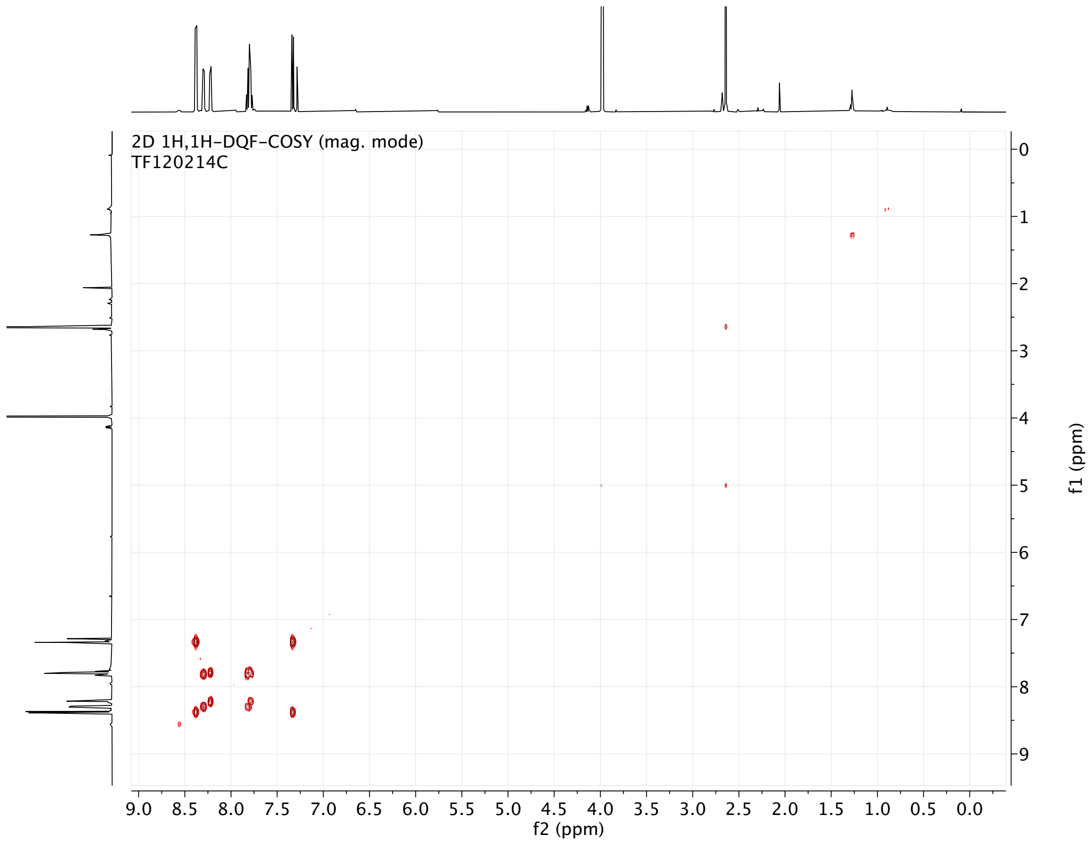

**(9b)**

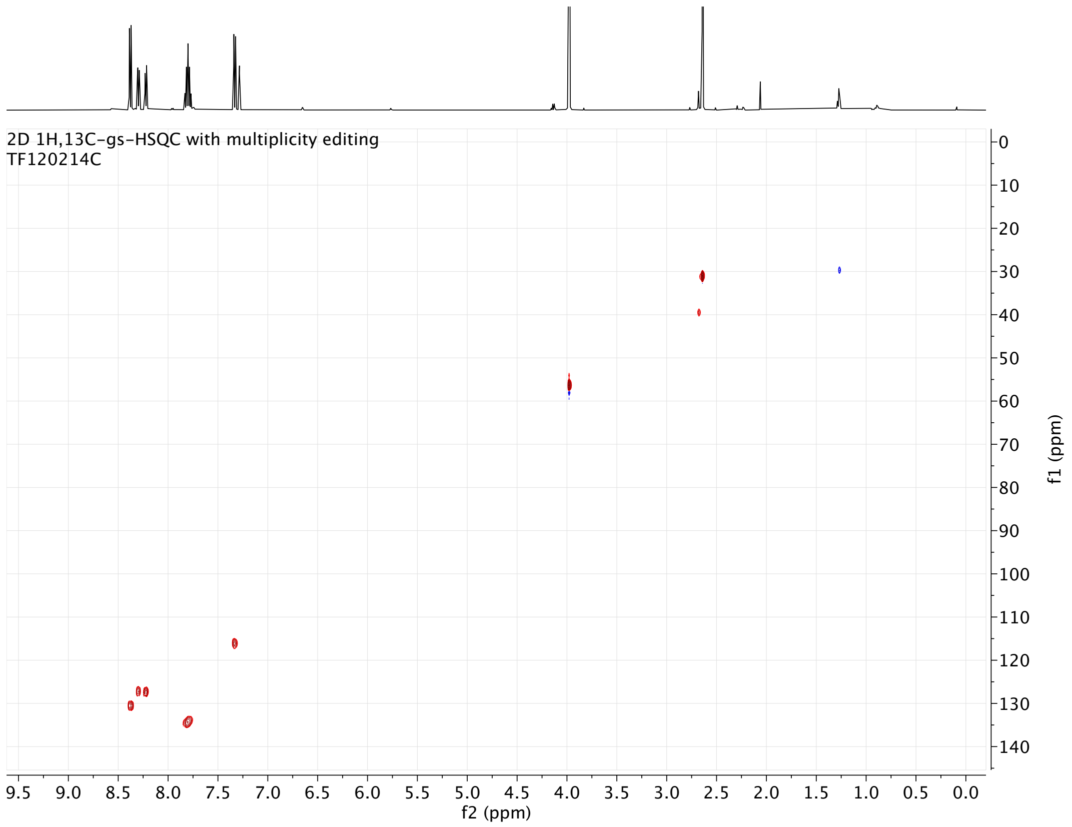
2D 1H,13C-gs-HSQC with multiplicity editing
TF120214C
f2 (ppm)
f1 (ppm)

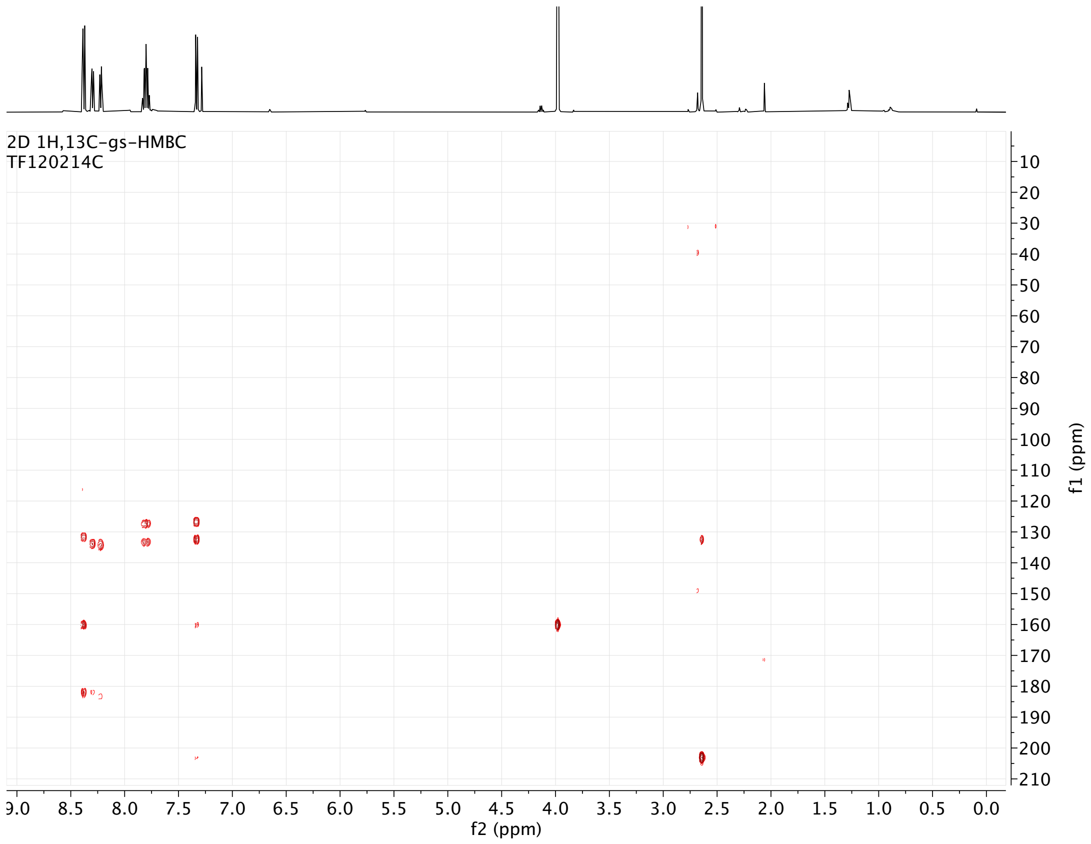
2D 1H,13C-gs-HMBC
TF120214C
f2 (ppm)
f1 (ppm)

**(10)**

6-Methoxy-4-methyl-3a,4,8,8a-tetrahydro-4,8-epoxycyclohepta[c]pyrrole-1,3,5(2H)-trione (**10**)

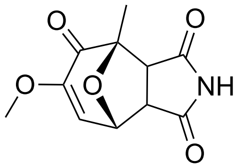

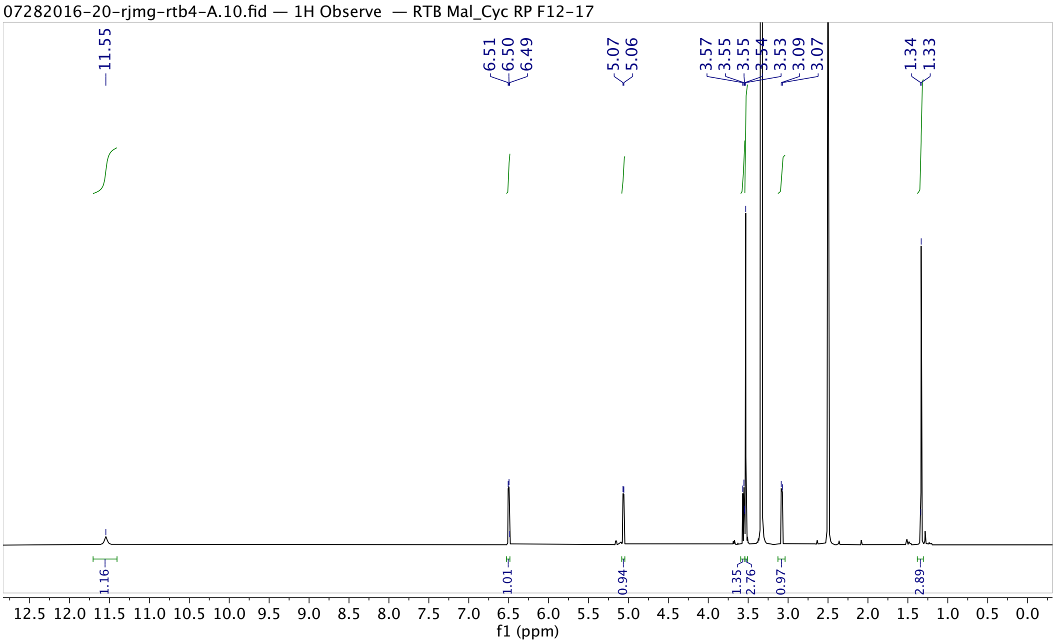


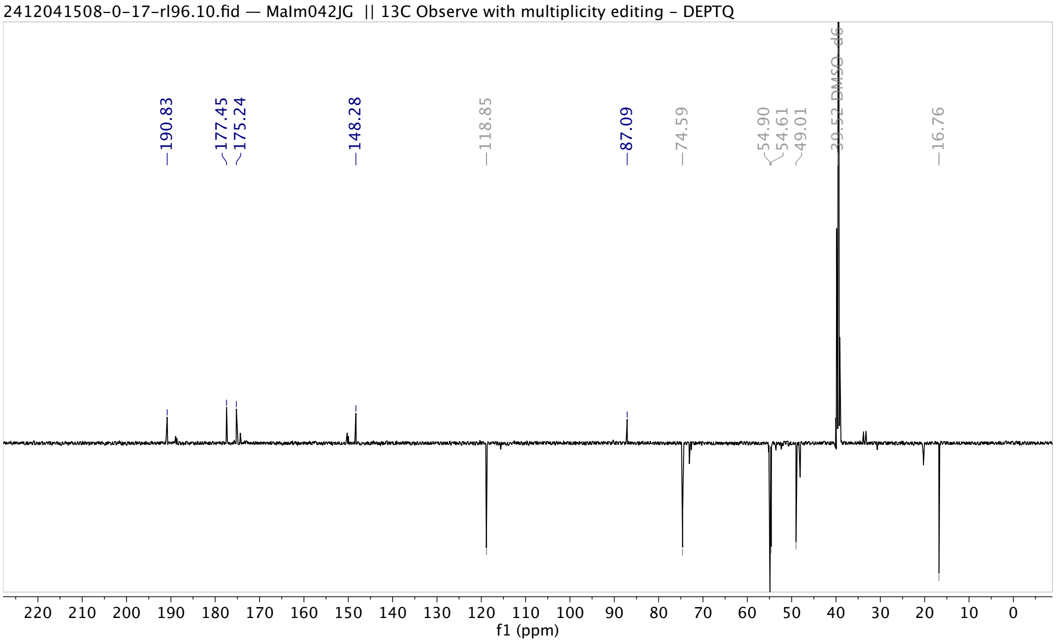

**(10)**

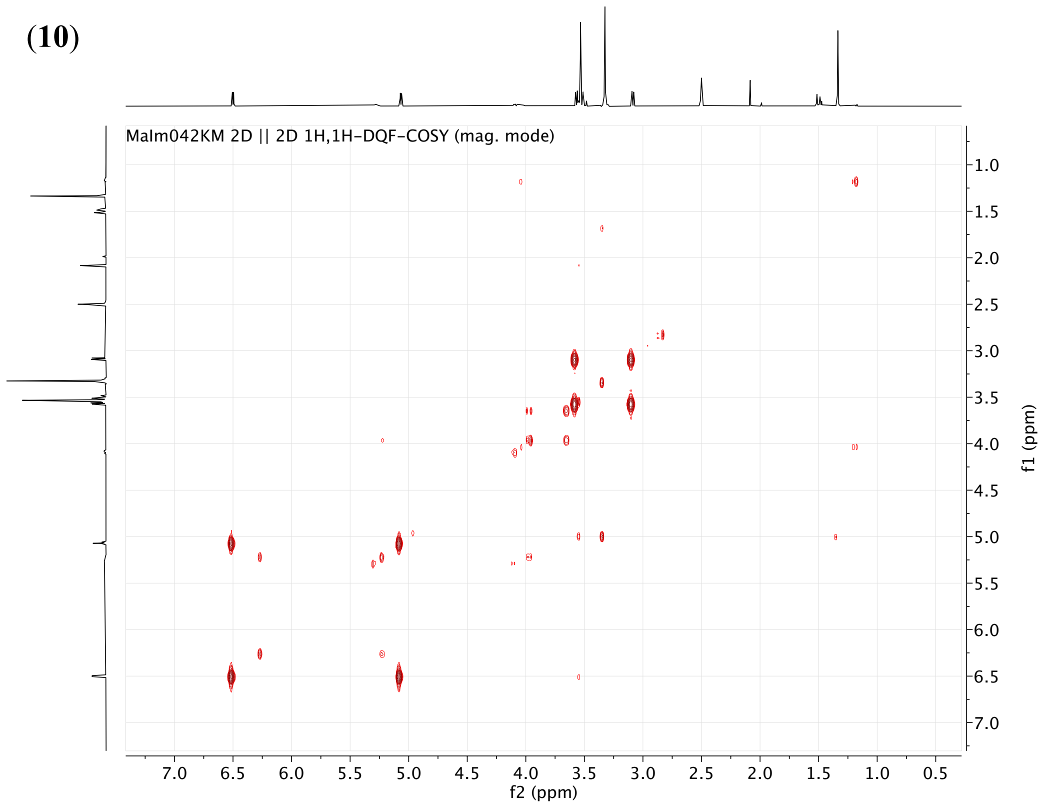
MaIm042KM 2D || 2D 1H,1H-DQF-COSY (mag. mode)
f1 (ppm)
f2 (ppm)

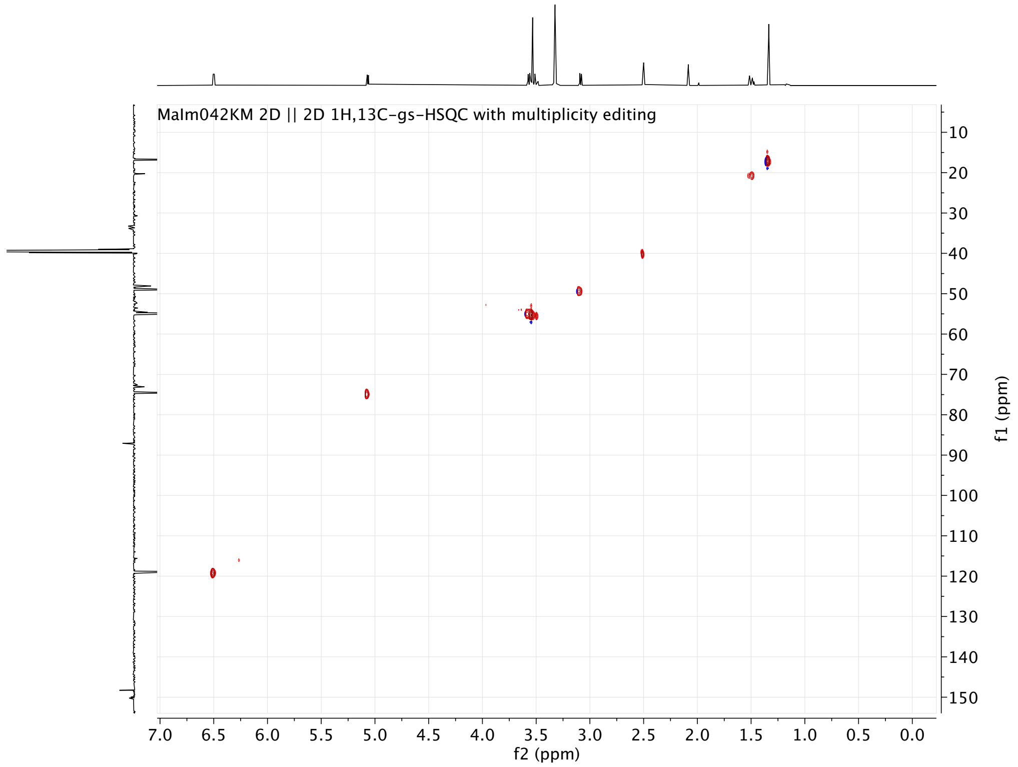
MaIm042KM 2D || 2D 1H,13C-gs-HSQC with multiplicity editing
f1 (ppm)
f2 (ppm)

(**10**)

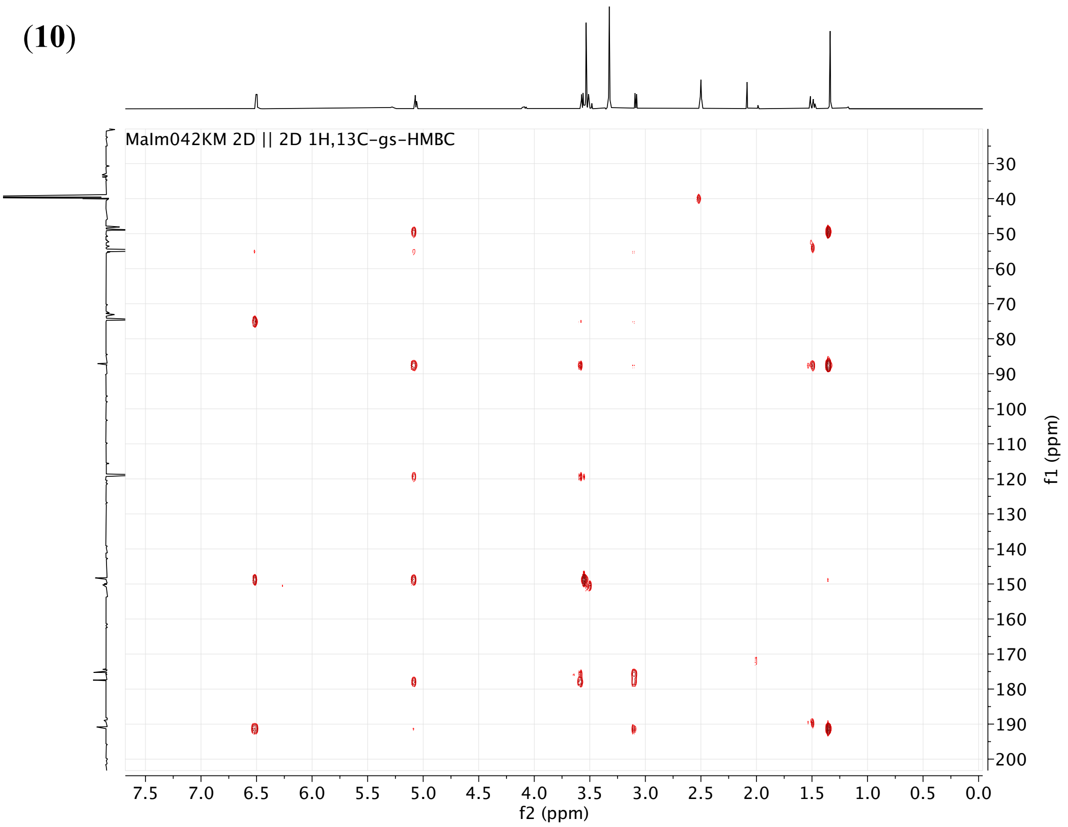


(**11**)

6-Methoxy-4-methyl-2-phenyl-3a,4,8,8a-tetrahydro-4,8-epoxycyclohepta[*c*]pyrrole-1,3,5(2*H*)-trione (**11**)

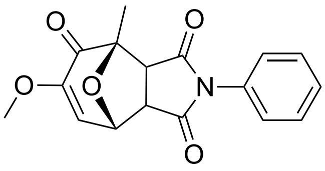

**(11)**

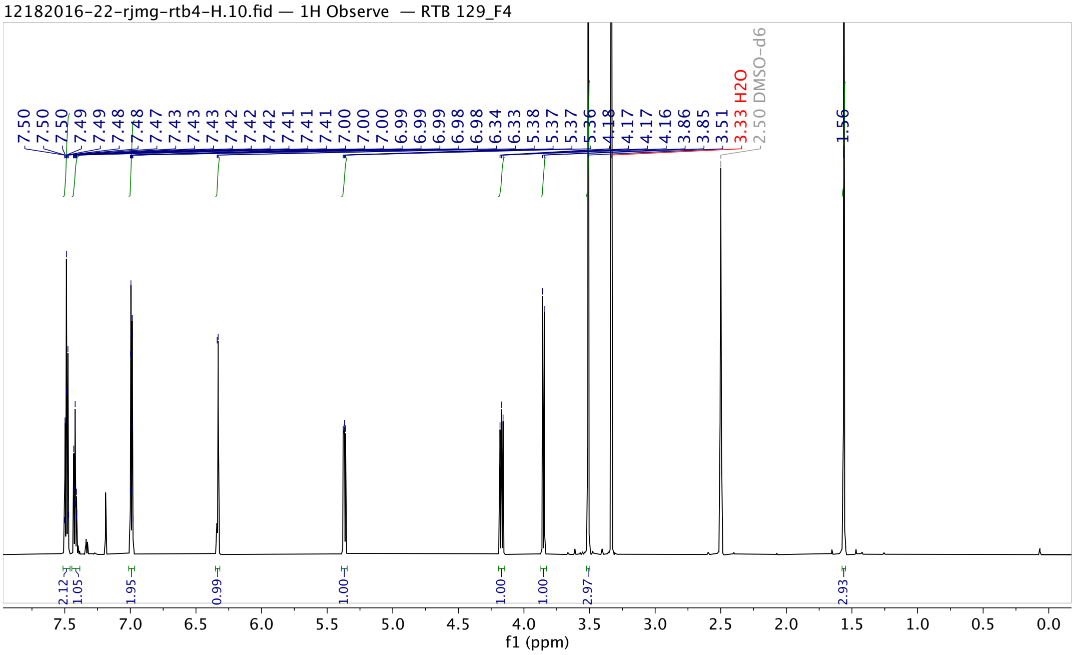

12182016-22-rjmg-rtb4-H.10.fid — 1H Observe — RTB 129_F4
7.50
7.50
7.50
7.49
7.49
7.48
7.48
7.47
7.43
7.43
7.43
7.42
7.42
7.42
7.41
7.41
7.41
7.00
7.00
7.00
6.99
6.99
6.99
6.98
6.98
6.34
6.33
5.38
5.37
5.37
5.36
4.18
4.17
4.17
4.16
3.86
3.85
3.51
3.33 H2O
2.50 DMSO-d6
1.56
2.12
1.05
1.95
0.99
1.00
1.00
1.00
2.97
2.93
7.5
7.0
6.5
6.0
5.5
5.0
4.5
4.0
3.5
3.0
2.5
2.0
1.5
1.0
0.5
0.0
f1 (ppm)


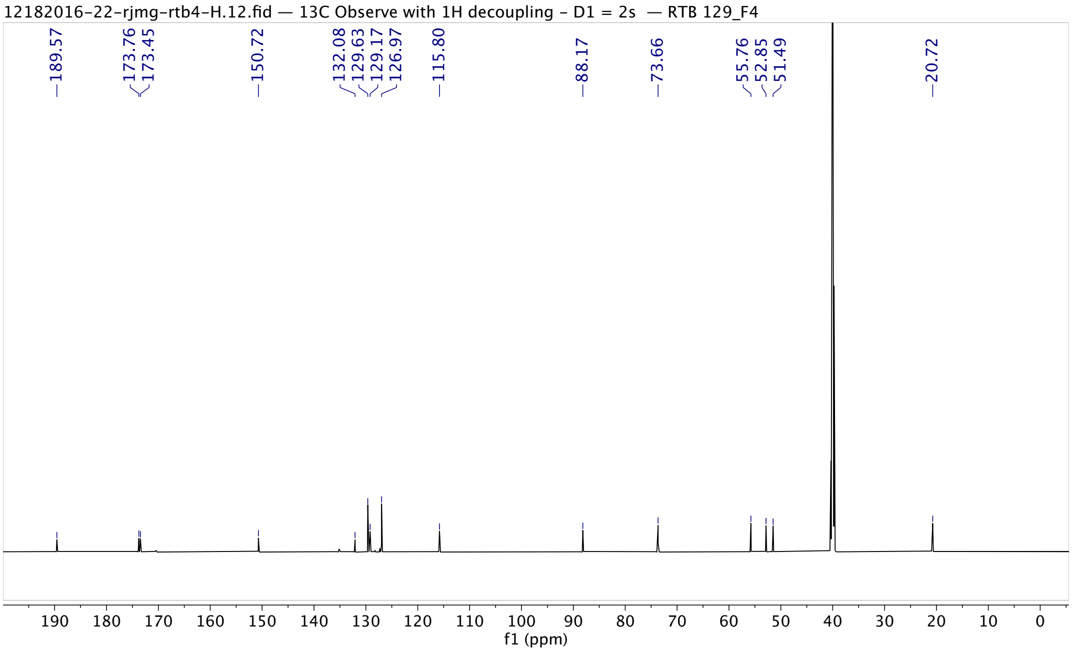

12182016-22-rjmg-rtb4-H.12.fid — 13C Observe with 1H decoupling - D1 = 2s — RTB 129_F4
189.57
173.76
173.45
150.72
132.08
129.63
129.17
126.97
115.80
88.17
73.66
55.76
52.85
51.49
20.72
190
180
170
160
150
140
130
120
110
100
90
80
70
60
50
40
30
20
10
0
f1 (ppm)

(11)

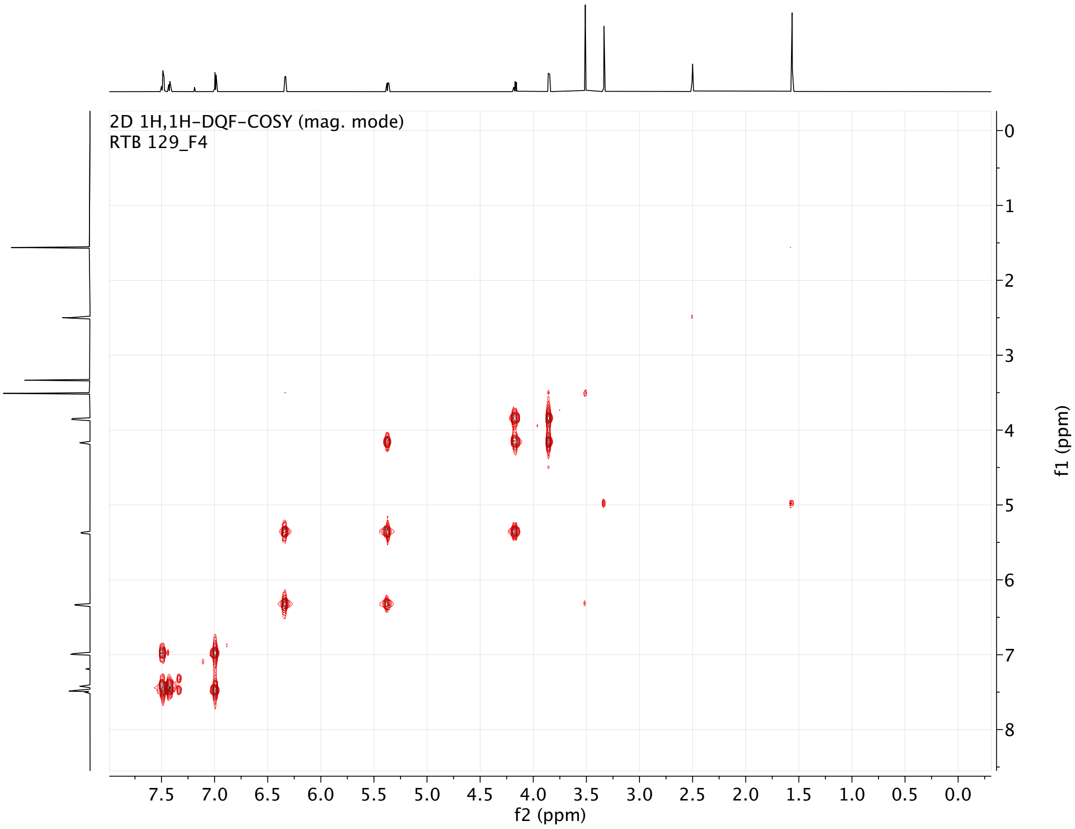
2D 1H,1H–DQF–COSY (mag. mode)
RTB 129_F4
f1 (ppm)
f2 (ppm)
7.5 7.0 6.5 6.0 5.5 5.0 4.5 4.0 3.5 3.0 2.5 2.0 1.5 1.0 0.5 0.0
0 1 2 3 4 5 6 7 8
